# Supplementary material for: Oncogenic RAS Signaling Promotes Tumor Immunoresistance by Stabilizing PD-L1 mRNA
Source: Immunity. 2017 Dec 19;47(6):1083–1099.e6. doi: 10.1016/j.immuni.2017.11.016 (PMC5746170; doi:10.1016/j.immuni.2017.11.016)
Supplement: Document S1. Figures S1–S7 and Tables S1 and S2 [file mmc1.pdf]

Immunity, Volume *47*

Supplemental Information

Oncogenic RAS Signaling Promotes Tumor Immunoresistance by Stabilizing PD-L1 mRNA

Matthew A. Coelho, Sophie de Carné Trécesson, Sareena Rana, Davide Zecchin, Christopher Moore, Miriam Molina-Arcas, Philip East, Bradley Spencer-Dene, Emma Nye, Karin Barnouin, Ambrosius P. Snijders, Wi S. Lai, Perry J. Blackshear, and Julian Downward

Supplemental Figure Legends

Figure S1, related to Figure 1. Cell-intrinsic Upregulation of PD-L1 through Oncogenic RAS Signalling

(**A**) qPCR analysis of PD-L1 mRNA expression at 6 h and 24 h after addition of 4-OHT or EtOH vehicle in ER-HRAS^{G12V} MCF10A cells. Mean ± SEM of two independent experiments. *****P*<0.0001; unpaired, two-tailed Student's *t*-tests.

(**B**) Flow cytometry analysis of PD-L1 surface protein expression at 24 h and four days after addition of 4-OHT or EtOH vehicle in ER-HRAS^{G12V} MCF10A cells and four days in ER-HRAS^{G12V} HKE-3 cells.

(**C**) Flow cytometry analysis of PD-L1 surface protein four days after addition of 4-OHT or EtOH vehicle to parental MCF10A and HKE-3 cells.

(**D**) Western blotting analysis of ER-KRAS^{G12V} type II pneumocytes treated with 4-OHT, MEK inhibitor or PI3K inhibitor in starvation medium for 24 h.

(**E**) qPCR analysis of PD-L1 expression in H23 and H1792 cells 24 h after addition of MEK inhibitor or PI3K inhibitor or the combination. Mean ± SEM of three (H23) or two (H1792) independent experiments. ***P*<0.005, **P*<0.05; unpaired, two-tailed Student's *t*-tests.

(**F**) qPCR analysis of PD-L1 expression in H358 and H23 cells 24 h after addition of the ERK1/2 inhibitor SCH772984 (500 nM). Mean ± SD of biological duplicates. ***P*<0.01; unpaired, two-tailed Student's *t*-tests.

(**G**) qPCR analysis of *PD-L1* expression in H1792 cells treated with PMA for 3 h following a 30 min pre-treatment with DMSO or MEK inhibitor. Data represent the mean ± SEM of two independent experiments. *****P*<0.0001; unpaired, two-tailed Student's *t*-test.

(**H**) Surface expression of PD-L1 was measured by flow cytometry. MFI values are adjusted for the isotype control in each condition. Mean ± SEM of biological duplicates.

(**I**) qPCR analysis of transcripts encoding antigen processing and presentation machinery in ER-KRAS^{G12V} type II pneumocytes simulated with 4-OHT for 24

h in starvation medium. Data represent the mean ± SEM of four independent experiments. **P<0.01; paired, two-tailed Student's *t*-test.

MFI, Mean Fluorescence Intensity. 4-OHT, 100 nM. IFN-γ, 20 ng/ml. MEK inhibitor GSK1120212, 25 nM. PI3K inhibitor GDC-0941, 500 nM. PMA, 200 nM.

Figure S2, relating to Figure 2. RAS Signalling Increases PD-L1 mRNA Stability through AU-rich Elements in the 3'UTR

(**A**) Normalised luciferase signal from the indicated human *CD274* promoter region reporter constructs in H358 cells treated for 6.5 h with medium only, PMA (200 nM) or IFN-γ (20 ng/ml). Numbering corresponds to the GRCh38 assembly. Data are representative of two independent experiments. **P<0.005, two-way ANOVA.

(**B**) Stability of murine PD-L1 mRNA measured by qPCR after the addition of actinomycin D (5 µg/ml) and DMSO or PI3K inhibitor GDC-0941 (500 nM). KPB6 cells were pre-treated with DMSO or PI3K inhibitor for 30 min before actinomycin D addition. Data represent the mean ± SEM and are normalised to the 0 h time point when actinomycin D was added, and are representative of two independent experiments.

(**C**) Stability of murine Tusc2 mRNA (left panel) and Ptgs2 mRNA (right panel) measured by qPCR after the addition of actinomycin D (5 µg/ml) and DMSO or MEK inhibitor GSK1120212 (25 nM). KPB6 cells were pre-treated with DMSO or MEK inhibitor for 30 min before actinomycin D addition. Data represent the mean ± SEM and are normalised to the 0 h time point when actinomycin D was added.

Figure S3, relating to Figure 3. AU-rich element Binding Proteins TTP and KSRP are Negative Regulators of PD-L1 Expression

(**A-C**) qPCR analysis of knockdown efficiency 48 h after siRNA transfections, relative to siScrambled control. Data represent the mean ± SD of triplicates.

(**D**) qPCR analysis of PD-L1 expression 24 h after transfection with the indicated constructs. Data represent the mean ± SEM of two independent experiments. *****P*<0.0001; ***P*<0.01; unpaired, two-tailed Student's *t*-test.

(**E**) qPCR analysis of PD-L1 expression in H23 cells 48 h after transfection with siRNAs targeting AU-rich binding proteins (AU-BPs), relative to siScrambled (siSc) control. Data represent the mean ± SEM of two independent experiments.

(**F**) qPCR analysis of knock-down efficiency in H23 cells 48 h after siRNA transfections, relative to siScrambled control. Data represent the mean ± SEM of two independent experiments.

(**G**) Western blotting analysis of TTP expression in H23, A427 and H358 cells 48 h after siRNA transfection with siRNA pools against TTP relative to siScrambled. Overexpression of Myc-TTP serves as a positive control for immunodetection.

(**H**) qPCR analysis of PD-L1 and TTP expression in H23 and H358 cells 48 h after siRNA transfection with siRNA pools or single siRNAs against TTP relative to siScrambled. Data represent the mean ± SEM of biological duplicates.

Figure S4, relating to Figure 4. RAS Regulates PD-L1 Expression through TTP

(**A**) qPCR analysis of PD-L1 and KSRP expression in H358 cells following siRNA mediated knock-down of KSRP (24 h) followed by MEK inhibition (24 h) with GSK1120212 (25 nM). Data represent the mean ± SEM of two independent experiments. ****P<0.0001; ***P<0.001; **P<0.005; *P<0.05; n.s; not significant.

(**B**) qPCR analysis of PD-L1 mRNA from RNA immunoprecipitates using IgG control or anti-TTP antibody, or anti-KSRP antibody, using KPB6 cells pre-treated with DMSO or MEK inhibitor GSK1120212 for 5.5 h (25 nM). Data represent the mean ± SD of biological triplicate IPs.

(**C**) qPCR analysis of Gapdh mRNA (control, lacking AU-rich elements) from RNA immunoprecipitates using IgG control or anti-TTP antibody, or anti-KSRP antibody, using KPB6 cells pre-treated with DMSO or MEK inhibitor GSK1120212 for 5.5 h (25 nM). Data represent the mean ± SD of biological triplicate IPs.

(**D**) qPCR analysis of PD-L1 expression in H358 cells 24 after transfection with empty, wild-type KSRP or phospho-mutant KSRP^{S193A} constructs. Data represent the mean ± SEM of two independent experiments. ****P<0.0001; ***P<0.001; NS, not significant.

Unless otherwise stated, data were compared using unpaired, two-tailed Student's *t*-tests.

Figure S5, relating to Figure 5. RAS-ROS-p38 Signalling Controls TTP Activity

(**A**) MS/MS spectra for phosphopeptides STphSLVEGR (S52) and QSIphSFSGLPSGR (S178). -98 indicates the loss of H_3PO_4.

(**B**) Flow cytometry analysis of PD-L1 surface protein, and intracellular ROS measured by staining with H_2DCFDA, in MCF10A ER-HRASG12V cells treated with 4-OHT or vehicle ± NAC (10 mM) for 24 h. The same dataset is represented as a dot-plot and a histogram and data are representative of two independent experiments.

(**C**) Western blotting analysis of MCF10A cells harbouring an inducible version of the kinase domain of MEKK3 (ER-ΔMEKK3), 24 h after the addition of 4-OHT (100 nM) or vehicle.

(**D**) qPCR analysis of PD-L1 mRNA expression 24 h after treatment with NAC (10 mM), reduced glutathione (1 mM) or MK2 inhibitor III (1 μM). Data represent the mean ± SEM of two independent experiments. ***P*< 0.01, ****P*<0.005, *****P*<0.001; two-tailed Student's *t*-tests comparing to DMSO control condition.

(**E**) Western blotting analysis of CT26 TTP KO cells harbouring doxycycline-inducible WT or phospho-mutant Myc-TTP constructs, treated with doxycycline or vehicle for 24 h. Arrow indicates Myc-TTP.

Figure S6, relating to Figure 6. RAS Pathway Activation is Associated with PD-L1 Upregulation in Human Cancers

(**A**) Heat maps showing LUAD and COAD samples from the TCGA dataset clustered into RAS high or low pathway activity groups using RNA sequencing expression data and published RAS activity gene expression signatures (Loboda et al., 2010; Sweet-Cordero et al., 2005). *KRAS* mutation status (codons 12, 13 and 61) is shown for each sample.

(**B**) qPCR analysis of PD-L1 and IFNGR1 expression in ER-KRAS^{G12V} type II pneumocytes 24 h after treatment with vehicle, 4-OHT (100 nM), or 4-OHT with IFN-γ blocking antibody (10 µg/ml) or with ruxolitinib (500 nM). Mean ± SEM of two independent experiments. The panel on the right shows a qPCR analysis of PD-L1 expression in ER-KRAS^{G12V} type II pneumocytes following a 30 h treatment with IFN-γ (20 ng/ml) and IFN-γ with IFN-γ blocking antibody (10 µg/ml) or with ruxolitinib (500 nM) to verify blocking of IFN-γ – IFGR1 signalling.

(**C**) TTP mRNA expression in human patient lung and colon normal tissue versus adenocarcinoma, from publically available datasets (Selamat et al., 2012; Skrzypczak et al., 2010). Wilcoxon signed-rank test.

(**D**) qPCR analysis of PD-L1 and TTP expression in FACS purified CD45$^-$CD31$^-$DAPI$^-$EpCAM$^+$ cells derived from lung tumours or matched normal adjacent lung tissue from *Kras*$^{LSL\text{-}G12D/+}$; *Trp53*$^{F/F}$ mice. Each point represents data from an individual mouse and is normalised to the matched normal lung tissue. ***P<0.0005; unpaired, two-tailed Student's *t*-tests.

(**E**) Flow cytometry analysis of PD-L1 expression on CD45-CD31-DAPI- cells derived from macroscopically dissected lung tumours or normal adjacent lung tissue from *Kras*$^{LSL\text{-}G12D/+}$; *Trp53*$^{F/F}$ mice. Each point represents data from an individual mouse and is normalised to the matched normal lung tissue. Data are pooled from two independent experiments. MFI; Mean Fluorescence Intensity. *P<0.05; unpaired, two-tailed Student's *t*-test.

Figure S7, relating to Figure 7. Restoration of Tumour Cell TTP Expression Enhances Anti-tumour Immunity

(**A**) Stability of murine PD-L1 mRNA measured by qPCR analysis. CT26 TTP (tetON) cells were pretreated with doxycycline (Dox.; 1 µg/ml) or vehicle for 16 h and then MEK inhibitor (GSK1120212, trametinib; 25 nM) for an additional 30 min before actinomycin D (ActD; 10 µg/ml) was added. Data are normalised to time 0 h when ActD was added and represent the mean ± SEM of two independent experiments.

(**B**) Western blotting analysis of stable MC38 cell lines expressing Myc-tagged, mouse TTP under a tetracycline-inducible promoter (TTP tet-ON). Cells were treated with doxycycline (Dox., 0.1 µg/ml or 1 µg/ml) or vehicle in starvation medium for 24 h before analysis. Arrow indicates Myc-TTP.

(**C**) qPCR analysis of PD-L1 expression from stable MC38 (TTP tet-ON) cell lines treated with doxycycline (Dox., 1 µg/ml) or vehicle in starvation medium for 40 h before analysis. Data represent the mean ± SEM of biological duplicates.

(**D**) Confluency was measured using IncuCyte for CT26 stable derivative cell lines treated with the indicated concentrations of doxycycline or vehicle at t = 0 h. Data represent the mean ± SD of biological triplicates and are representative of two independent experiments.

(**E**) Confluency was measured using IncuCyte for MC38 stable derivative cell lines treated with the indicated concentrations of doxycycline or vehicle at t = 0 h. Data represent the mean ± SD of biological triplicates.

(**F**) Representative histograms from flow cytometry analysis of PD-L1 surface expression in TTP (tetON) CT26 stable cells lines expressing endogenous PD-L1, PD-L1 + wild-type 3'UTR cDNA, or PD-L1 Δ3'UTR cDNA, after treatment with doxycycline (Dox., 1 µg/ml) or vehicle for 72 h. Data are representative of two independent experiments and also form part of Figure 7B.

(**G**) Tumour growth curves for the indicated CT26-derived cell lines shown in Figure S7C, subcutaneously transplanted into BALB/c mice (*n* = 5 WT 3'UTR + Dox and Δ3'UTR + H_2O; *n* = 4 WT 3'UTR + H_2O and Δ3'UTR + Dox; *n* = 3

empty + H_2O and empty + Dox). Vehicle or doxycycline (Dox., 50 mg/kg) was administered by oral gavage and commenced from day three after tumour cell injection. Mean ± SEM. ****P<0.0001, ***P<0.001, *P<0.05; two-way ANOVA.
(**H**) Representative histological analysis of CD3+ cells in subcutaneous tumours at the end-point from the experiment described in Figure 7C, and quantified in Figure 7G. Scale bar is 500 μm.
(**I**) Proposed molecular model. Oncogenic RAS activity leads to hyperphosphorylation, whereas PP2A activity promotes hypophosphorylation of TTP (Bourcier et al., 2011; Deleault et al., 2008; Essafi-Benkhadir et al., 2007; Hardle et al., 2015; Rahman et al., 2015; Sun et al., 2007), constituting a rapid switch controlling TTP activity. Low TTP expression and activity in tumour cells represents a permissive context for PD-L1 expression and immune evasion.

Table S1, relating to Figure 5 and Figure S5. TTP phosphopeptides. Identified mouse TTP phosphopeptides from MS analyses pooled from two independent biological experiments. Identifications are 1 % FDR controlled. PEP indicates the probability that the identification is incorrect. Phosphosite assignment probabilities are indicated in parenthesis.

PEP	Score	Position	Modified sequence	Phospho (STY) Probabilities	Number of Phospho (STY)
0.01167	100	52	_STS(ph)LVEGR_	STS(1)LVEGR	1
1.41E-10	70	80	_PGPELS(ph)PSPT(ph)SPTATPTTSSR_	PGPELS(0.998)PS(0.006)PT(0.477)S(0.43)PT(0.068)AT(0.011)PT(0.002)T(0.002)S(0.002)S(0.003)R	2
1.41E-10	69	82	_PGPELSPS(ph)PTSPTATPTTSSR_	PGPELS(0.036)PS(0.911)PT(0.04)S(0.009)PT(0.002)AT(0.001)PTTSSR	1
1.07E-52	186	85	_PGPELSPSPTS(ph)PTATPTTSSR_	PGPELSPSPT(0.128)S(0.857)PT(0.013)ATPTTSSR	1
0.0404	74	105	_TYS(ph)ESGRCR_	TYS(1)ESGRCR	1
6.90E-08	130	178	_QSIS(ph)FSGLPSGR_	QSIS(1)FSGLPSGR	1
1.50E-22	96	189	_RSS(ph)PPPPGFS(ph)GPSLSSCSFSPSSSPPPPGDLPLSPSAFSAAPGTPVTR_	RS(0.17)S(0.767)PPPPGFS(0.195)GPS(0.146)LS(0.131)S(0.124)CS(0.111)FS(0.1)PS(0.09)S(0.085)S(0.081)PPPPGDLPLSPSAFSAAPGTPVTR	2
1.50E-22	96	196	_RSSPPPPGFS(ph)GPS(ph)LSSCSFSPSSSPPPPGDLPLSPSAFSAAPGTPVTR_	RS(0.051)S(0.25)PPPPGFS(0.367)GPS(0.173)LS(0.169)S(0.167)CS(0.166)FS(0.165)PS(0.165)S(0.164)S(0.164)PPPPGDLPLSPSAFSAAPGTPVTR	2
1.50E-22	96	199	_RSS(ph)PPPPGFS(ph)GPS(ph)LSSCSFSPSSSPPPPGDLPLSPSAFSAAPGTPVTR_	RS(0.072)S(0.368)PPPPGFS(0.279)GPS(0.303)LS(0.3)S(0.297)CS(0.29)FS(0.282)PS(0.274)S(0.27)S(0.265)PPPPGDLPLSPSAFSAAPGTPVTR	2
9.68E-15	69	201	_RSSPPPPGFS(ph)GPS(ph)LSSCSFSPSSSPPPPGDLPLSPSAFSAAPGTPVTR_	RS(0.013)S(0.177)PPPPGFS(0.202)GPS(0.201)LS(0.201)S(0.201)CS(0.201)FS(0.201)PS(0.201)S(0.201)S(0.201)PPPPGDLPLSPSAFSAAPGTPVTR	2
9.68E-15	69	202	_RSSPPPPGFS(ph)GPS(ph)LSSCSFSPSSSPPPPGDLPLSPSAFSAAPGTPVTR_	RS(0.013)S(0.177)PPPPGFS(0.202)GPS(0.201)LS(0.201)S(0.201)CS(0.201)FS(0.201)PS(0.201)S(0.201)S(0.201)PPPPGDLPLSPSAFSAAPGTPVTR	2
9.68E-15	69	204	_RSSPPPPGFS(ph)GPS(ph)LSSCSFSPSSSPPPPGDLPLSPSAFSAAPGTPVTR_	RS(0.013)S(0.177)PPPPGFS(0.202)GPS(0.201)LS(0.201)S(0.201)CS(0.201)FS(0.201)PS(0.201)S(0.201)S(0.201)PPPPGDLPLSPSAFSAAPGTPVTR	2
9.68E-15	69	206	_RSSPPPPGFS(ph)GPS(ph)LSSCSFSPSSSPPPPGDLPLSPSAFSAAPGTPVTR_	RS(0.013)S(0.177)PPPPGFS(0.202)GPS(0.201)LS(0.201)S(0.201)CS(0.201)FS(0.201)PS(0.201)S(0.201)S(0.201)PPPPGDLPLSPSAFSAAPGTPVTR	2
9.68E-15	69	208	_RSSPPPPGFS(ph)GPS(ph)LSSCSFSPSSSPPPPGDLPLSPSAFSAAPGTPVTR_	RS(0.013)S(0.177)PPPPGFS(0.202)GPS(0.201)LS(0.201)S(0.201)CS(0.201)FS(0.201)PS(0.201)S(0.201)S(0.201)PPPPGDLPLSPSAFSAAPGTPVTR	2
9.68E-15	69	209	_RSSPPPPGFS(ph)GPS(ph)LSSCSFSPSSSPPPPGDLPLSPSAFSAAPGTPVTR_	RS(0.013)S(0.177)PPPPGFS(0.202)GPS(0.201)LS(0.201)S(0.201)CS(0.201)FS(0.201)PS(0.201)S(0.201)S(0.201)PPPPGDLPLSPSAFSAAPGTPVTR	2
9.68E-15	69	210	_RSSPPPPGFS(ph)GPS(ph)LSSCSFSPSSSPPPPGDLPLSPSAFSAAPGTPVTR_	RS(0.013)S(0.177)PPPPGFS(0.202)GPS(0.201)LS(0.201)S(0.201)CS(0.201)FS(0.201)PS(0.201)S(0.201)S(0.201)PPPPGDLPLSPSAFSAAPGTPVTR	2
0.00722	63	248	_S(ph)TTPSTIWGPLGGLAR_	S(0.333)T(0.333)T(0.333)PSTIWGPLGGLAR	1
0.00722	63	249	_S(ph)TTPSTIWGPLGGLAR_	S(0.333)T(0.333)T(0.333)PSTIWGPLGGLAR	1
1.93E-20	158	250	_STT(ph)PSTIWGPLGGLAR_	ST(0.003)T(0.997)PSTIWGPLGGLAR	1
6.07E-19	89	264	_S(ph)PSAHSLGSDPDDYASSGSSLGGSDSPVFEAGVFGPPQTPAPPR_	S(0.446)PS(0.412)AHS(0.087)LGS(0.022)DPDDY(0.004)AS(0.005)S(0.005)GS(0.005)S(0.005)LGGS(0.005)DS(0.005)PVFEAGVFGPPQTPAPPR	1
6.07E-19	89	266	_S(ph)PSAHSLGSDPDDYASSGSSLGGSDSPVFEAGVFGPPQTPAPPR_	S(0.41)PS(0.41)AHS(0.165)LGS(0.006)DPDDY(0.001)AS(0.001)S(0.001)GS(0.001)S(0.001)LGGS(0.001)DS(0.001)PVFEAGVFGPPQTPAPPR	1
5.37E-14	83	269	_SPSAHS(ph)LGSDPDDYASSGSSLGGSDSPVFEAGVFGPPQTPAPPR_	S(0.272)PS(0.272)AHS(0.447)LGS(0.004)DPDDY(0.001)AS(0.001)S(0.001)GS(0.001)S(0.001)LGGS(0.001)DS(0.001)PVFEAGVFGPPQTPAPPR	1
1.50E-07	59	272	_SPSAHSLGS(ph)DPDDYASSGSSLGGSDSPVFEAGVFGPPQTPAPPR_	S(0.019)PS(0.019)AHS(0.078)LGS(0.343)DPDDY(0.073)AS(0.078)S(0.078)GS(0.078)S(0.078)LGGS(0.078)DS(0.078)PVFEAGVFGPPQTPAPPR	1
0.0015	41	279	_SPSAHSLGS(ph)DPDDYASSGSSLGGSDSPVFEAGVFGPPQTPAPPR_	S(0.044)PS(0.044)AHS(0.044)LGS(0.109)DPDDY(0.103)AS(0.109)S(0.109)GS(0.109)S(0.109)LGGS(0.109)DS(0.109)PVFEAGVFGPPQTPAPPR	1
0.0015	41	280	_SPSAHSLGS(ph)DPDDYASSGSSLGGSDSPVFEAGVFGPPQTPAPPR_	S(0.044)PS(0.044)AHS(0.044)LGS(0.109)DPDDY(0.103)AS(0.109)S(0.109)GS(0.109)S(0.109)LGGS(0.109)DS(0.109)PVFEAGVFGPPQTPAPPR	1
0.0015	41	282	_SPSAHSLGS(ph)DPDDYASSGSSLGGSDSPVFEAGVFGPPQTPAPPR_	S(0.044)PS(0.044)AHS(0.044)LGS(0.109)DPDDY(0.103)AS(0.109)S(0.109)GS(0.109)S(0.109)LGGS(0.109)DS(0.109)PVFEAGVFGPPQTPAPPR	1
0.0015	41	283	_SPSAHSLGS(ph)DPDDYASSGSSLGGSDSPVFEAGVFGPPQTPAPPR_	S(0.044)PS(0.044)AHS(0.044)LGS(0.109)DPDDY(0.103)AS(0.109)S(0.109)GS(0.109)S(0.109)LGGS(0.109)DS(0.109)PVFEAGVFGPPQTPAPPR	1
0.0015	41	287	_SPSAHSLGS(ph)DPDDYASSGSSLGGSDSPVFEAGVFGPPQTPAPPR_	S(0.044)PS(0.044)AHS(0.044)LGS(0.109)DPDDY(0.103)AS(0.109)S(0.109)GS(0.109)S(0.109)LGGS(0.109)DS(0.109)PVFEAGVFGPPQTPAPPR	1
0.0015	41	289	_SPSAHSLGS(ph)DPDDYASSGSSLGGSDSPVFEAGVFGPPQTPAPPR_	S(0.044)PS(0.044)AHS(0.044)LGS(0.109)DPDDY(0.103)AS(0.109)S(0.109)GS(0.109)S(0.109)LGGS(0.109)DS(0.109)PVFEAGVFGPPQTPAPPR	1

Table S2, relating to STAR Methods, Method Details. Cell lines and growth conditions.

Cell line	Normal medium	Starvation medium
H358	RPMI + 10 % FCS	N/A
A427	RPMI + 10 % FCS	N/A
H1792	RPMI + 10 % FCS	N/A
KPB6	DMEM + 10 % FCS	N/A
Type II pneumocytes	DCCM-1 + 10 % FCS	+ 0.5 % FCS for qPCR and FACS<br>+ 0 % FCS for mRNA half-life analysis
SW837	DMEM + 10 % FCS	N/A
H23	RPMI + 10 % FCS	N/A
293FT	DMEM + 10 % FCS	N/A
TTP KO and TTP WT MEFs	DMEM + 10 % FCS	+ 0.5 % FCS
MCF10A	F12:DMEM mix (1:1) and 5 % horse serum, 20 ng/ml EGF, 10 μg/ml insulin, 100 ng/ml cholera toxin, 0.5 μg/ml hydrocortisone	+ 5 % horse serum
CT26	RPMI + 10 % FCS	N/A
A549	DMEM + 10 % FCS	N/A
MC38	RPMI + 10 % FCS	+ 0.5 % FCS

N/A, not applicable.

Figure S1

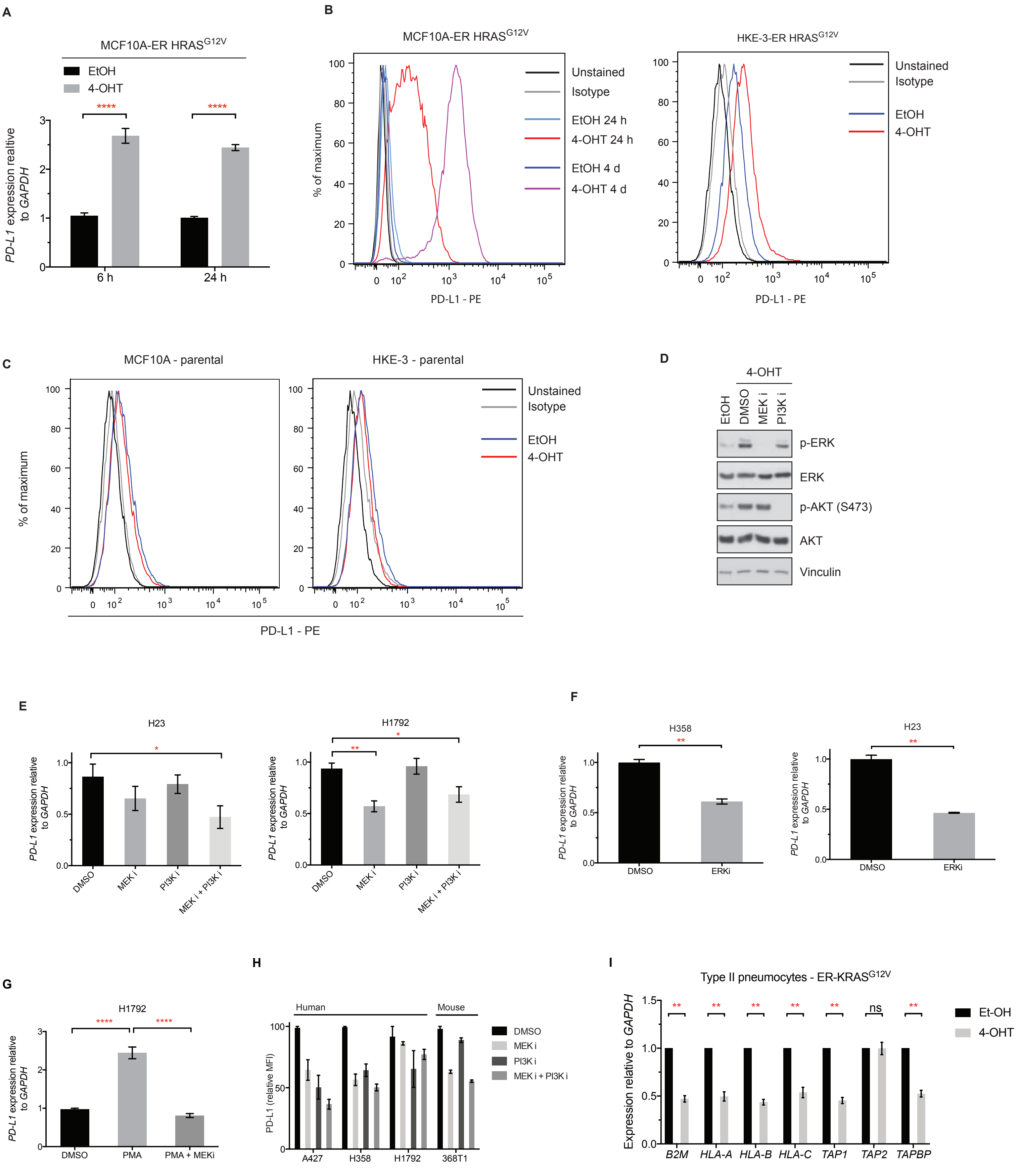
A
MCF10A-ER HRASG12V
EtOH
4-OHT
****
****
PD-L1 expression realtive to GAPDH
6 h
24 h
B
MCF10A-ER HRASG12V
Unstained
Isotype
EtOH 24 h
4-OHT 24 h
EtOH 4 d
4-OHT 4 d
% of maximum
PD-L1 - PE
HKE-3-ER HRASG12V
Unstained
Isotype
EtOH
4-OHT
PD-L1 - PE
C
MCF10A - parental
HKE-3 - parental
Unstained
Isotype
EtOH
4-OHT
% of maximum
PD-L1 - PE
D
4-OHT
EtOH
DMSO
MEK i
PI3K i
p-ERK
ERK
p-AKT (S473)
AKT
Vinculin
E
H23
H1792
PD-L1 expression relative to GAPDH
DMSO
MEK i
PI3K i
MEK i + PI3K i
F
H358
H23
DMSO
ERKi
G
H1792
DMSO
PMA
PMA + MEKi
H
Human
Mouse
PD-L1 (relative MFI)
DMSO
MEK i
PI3K i
MEK i + PI3K i
A427
H358
H1792
368T1
I
Type II pneumocytes - ER-KRASG12V
Expression relative to GAPDH
Et-OH
4-OHT
ns
B2M
HLA-A
HLA-B
HLA-C
TAP1
TAP2
TAPBP

Figure S2

A

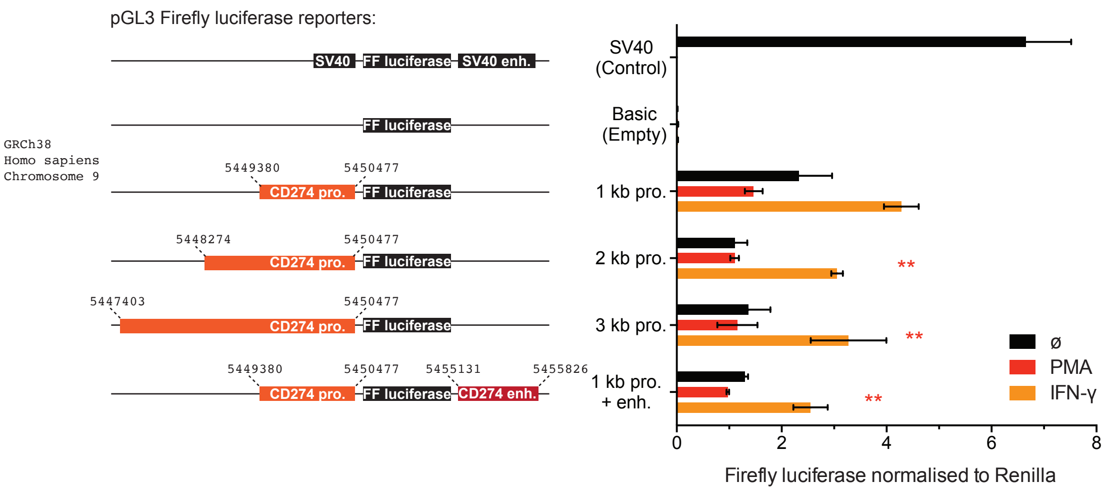

B

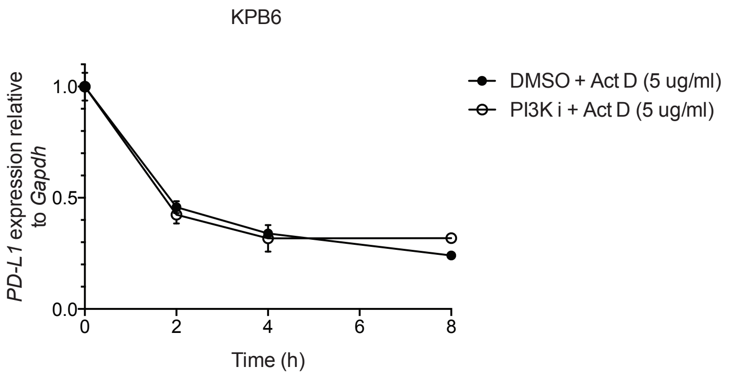

C

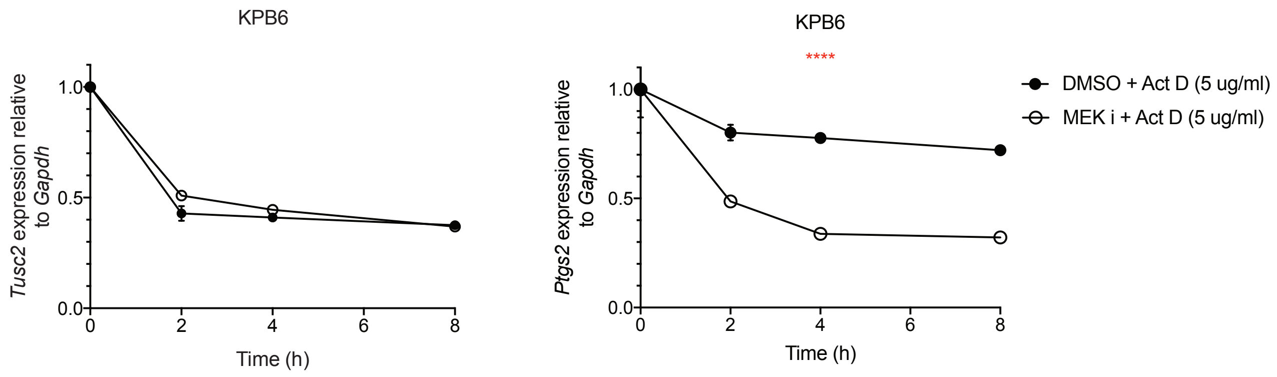

Figure S3

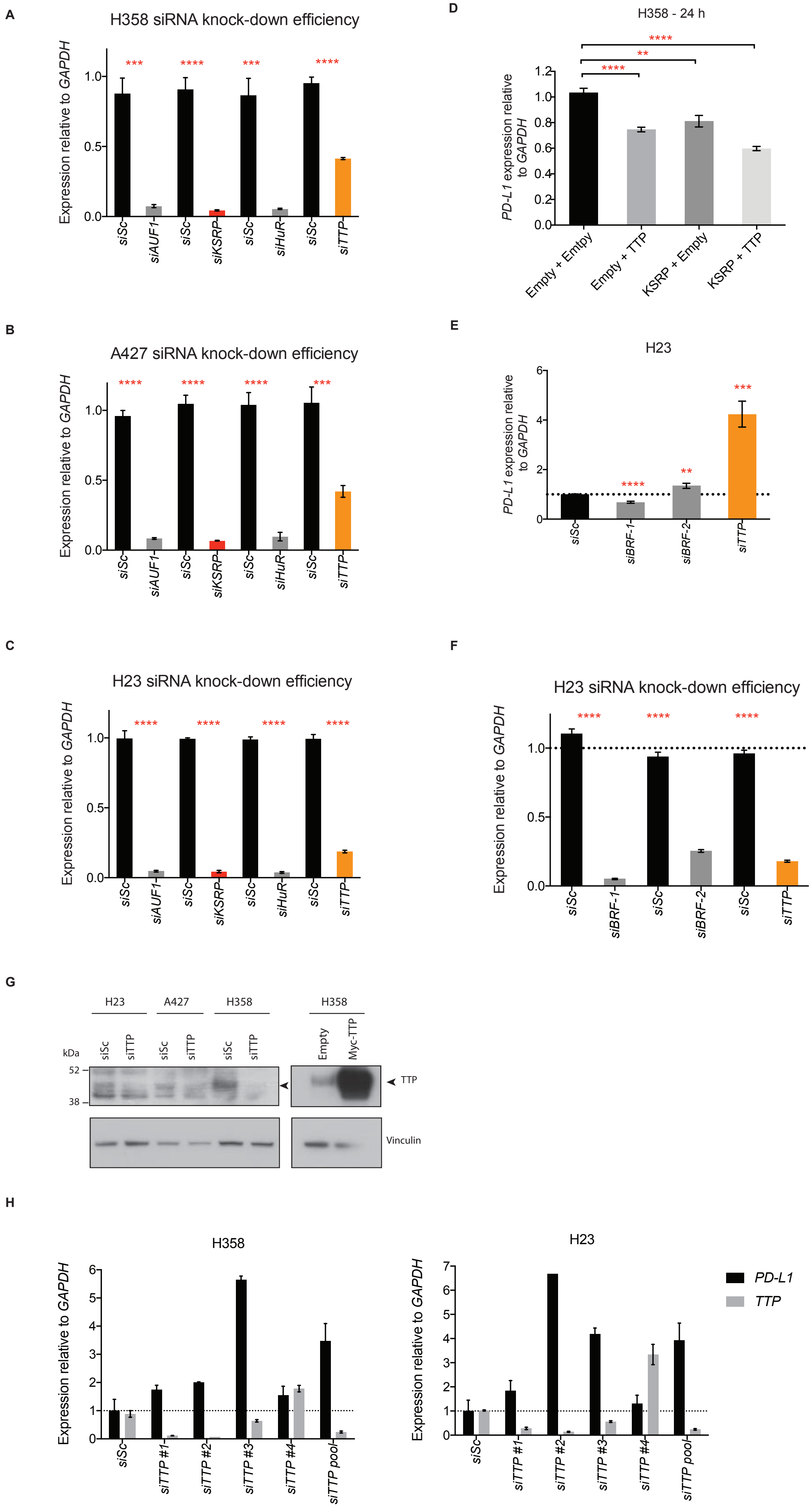
A
H358 siRNA knock-down efficiency
Expression relative to GAPDH
siSc
siAUF1
siKSRP
siHuR
siTTP
B
A427 siRNA knock-down efficiency
C
H23 siRNA knock-down efficiency
D
H358 - 24 h
PD-L1 expression relative to GAPDH
Empty + Empty
Empty + TTP
KSRP + Empty
KSRP + TTP
E
H23
siBRF-1
siBRF-2
F
H23 siRNA knock-down efficiency
G
H23
A427
H358
kDa
52
38
Empty
Myc-TTP
TTP
Vinculin
H
H358
H23
siTTP #1
siTTP #2
siTTP #3
siTTP #4
siTTP pool
PD-L1
TTP

Figure S4

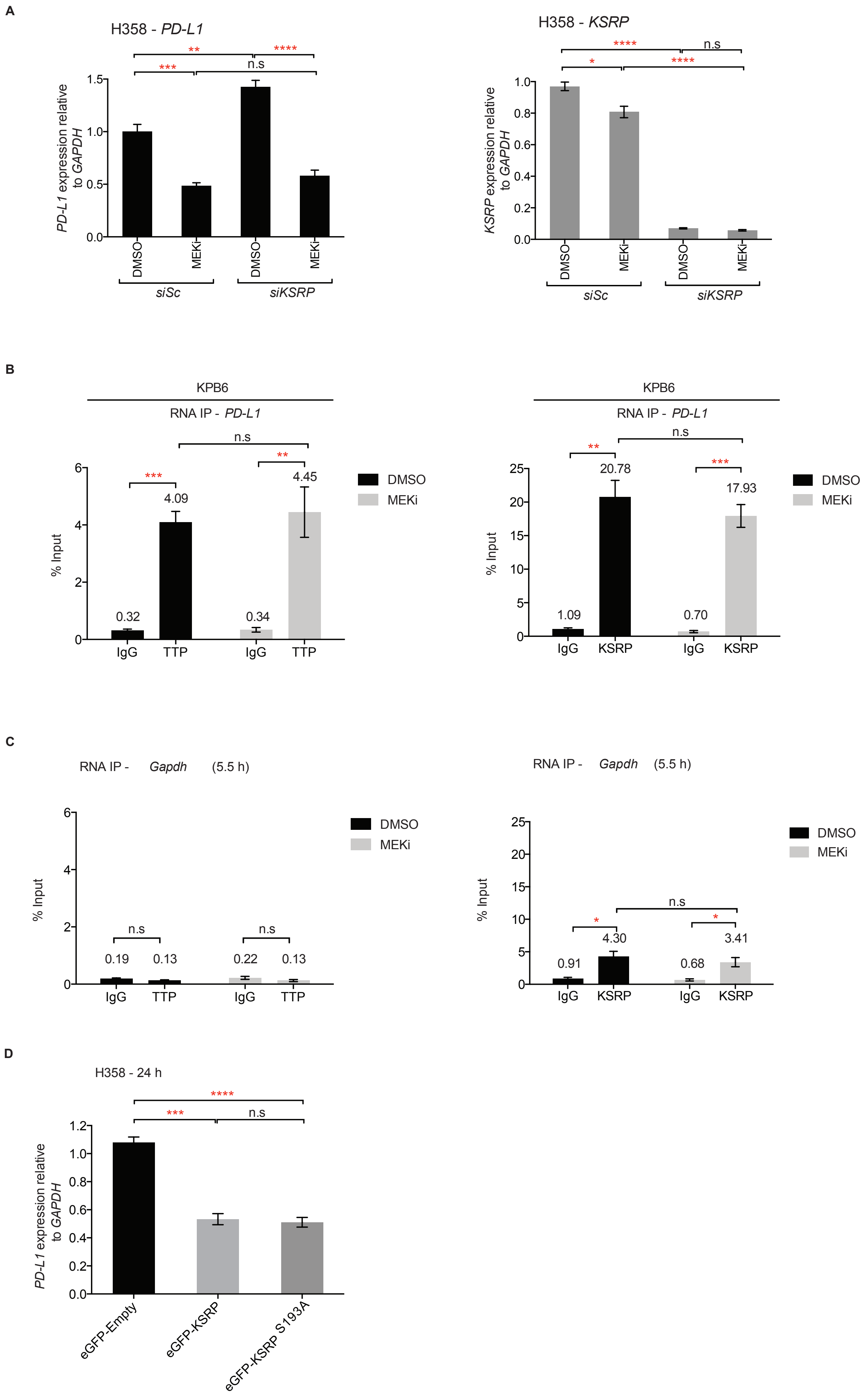

A
H358 - PD-L1
PD-L1 expression relative to GAPDH
1.5
1.0
0.5
0.0
**
****
***
n.s
DMSO
MEKi
DMSO
MEKi
siSc
siKSRP
H358 - KSRP
KSRP expression relative to GAPDH
1.0
0.8
0.6
0.4
0.2
0.0
****
n.s
*
****
DMSO
MEKi
DMSO
MEKi
siSc
siKSRP
B
KPB6
RNA IP - PD-L1
% Input
6
4
2
0
n.s
**
***
4.09
4.45
0.32
0.34
IgG
TTP
IgG
TTP
DMSO
MEKi
KPB6
RNA IP - PD-L1
% Input
25
20
15
10
5
0
n.s
**
***
20.78
17.93
1.09
0.70
IgG
KSRP
IgG
KSRP
DMSO
MEKi
C
RNA IP - Gapdh (5.5 h)
% Input
6
4
2
0
n.s
n.s
0.19
0.13
0.22
0.13
IgG
TTP
IgG
TTP
DMSO
MEKi
RNA IP - Gapdh (5.5 h)
% Input
25
20
15
10
5
0
n.s
*
*
4.30
3.41
0.91
0.68
IgG
KSRP
IgG
KSRP
DMSO
MEKi
D
H358 - 24 h
PD-L1 expression relative to GAPDH
1.2
1.0
0.8
0.6
0.4
0.2
0.0
****
***
n.s
eGFP-Empty
eGFP-KSRP
eGFP-KSRP S193A

Figure S5

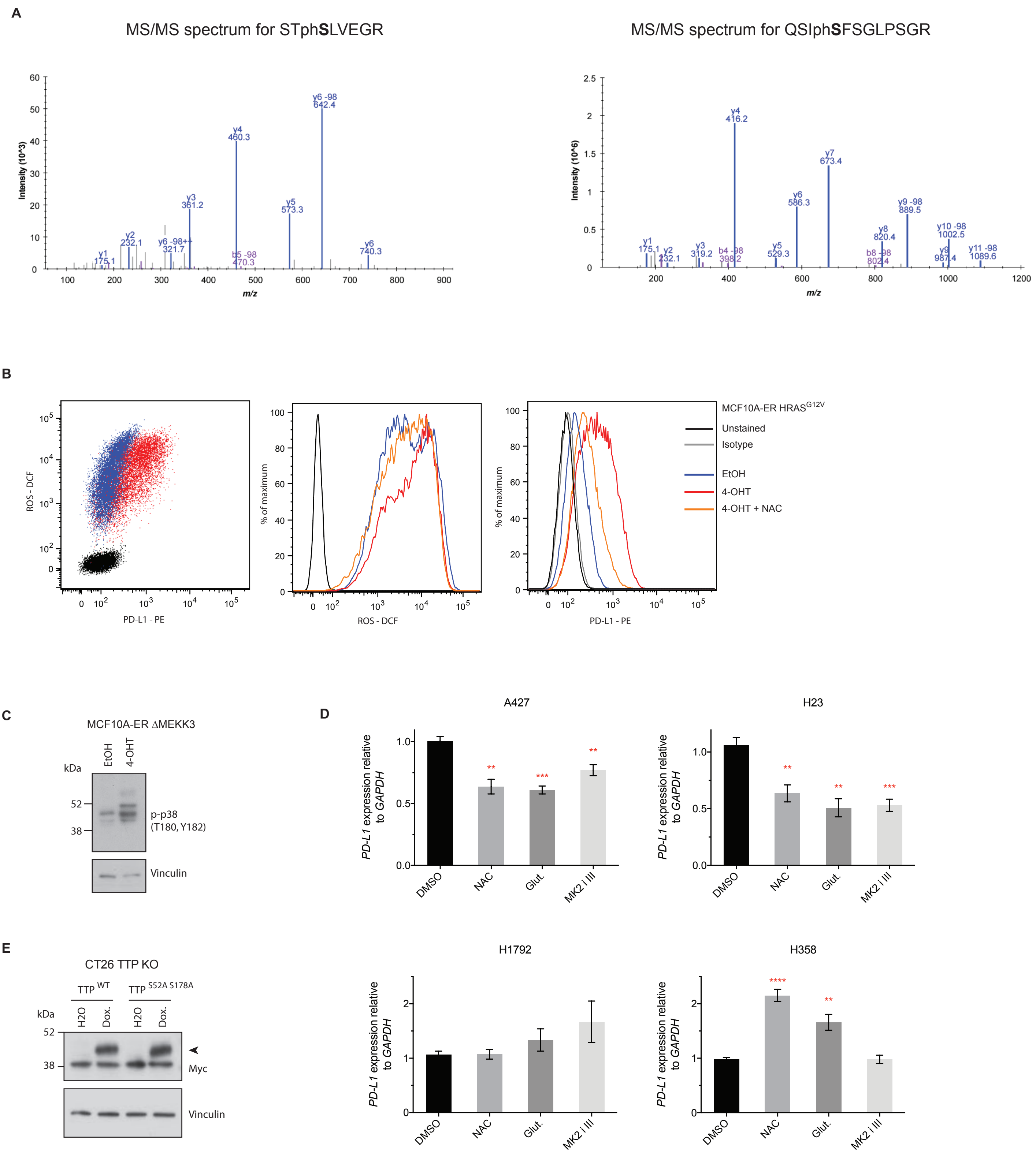
A
MS/MS spectrum for STph**S**LVEGR
MS/MS spectrum for QSIph**S**FSGLPSGR
Intensity (10^3)
Intensity (10^6)
m/z
B
ROS - DCF
PD-L1 - PE
% of maximum
MCF10A-ER HRAS G12V
Unstained
Isotype
EtOH
4-OHT
4-OHT + NAC
C
MCF10A-ER ΔMEKK3
EtOH
4-OHT
kDa
p-p38 (T180, Y182)
Vinculin
D
A427
H23
H1792
H358
PD-L1 expression relative to GAPDH
DMSO
NAC
Glut.
MK2 i III
E
CT26 TTP KO
TTP WT
TTP S52A S178A
H2O
Dox.
Myc
Vinculin

Figure S6

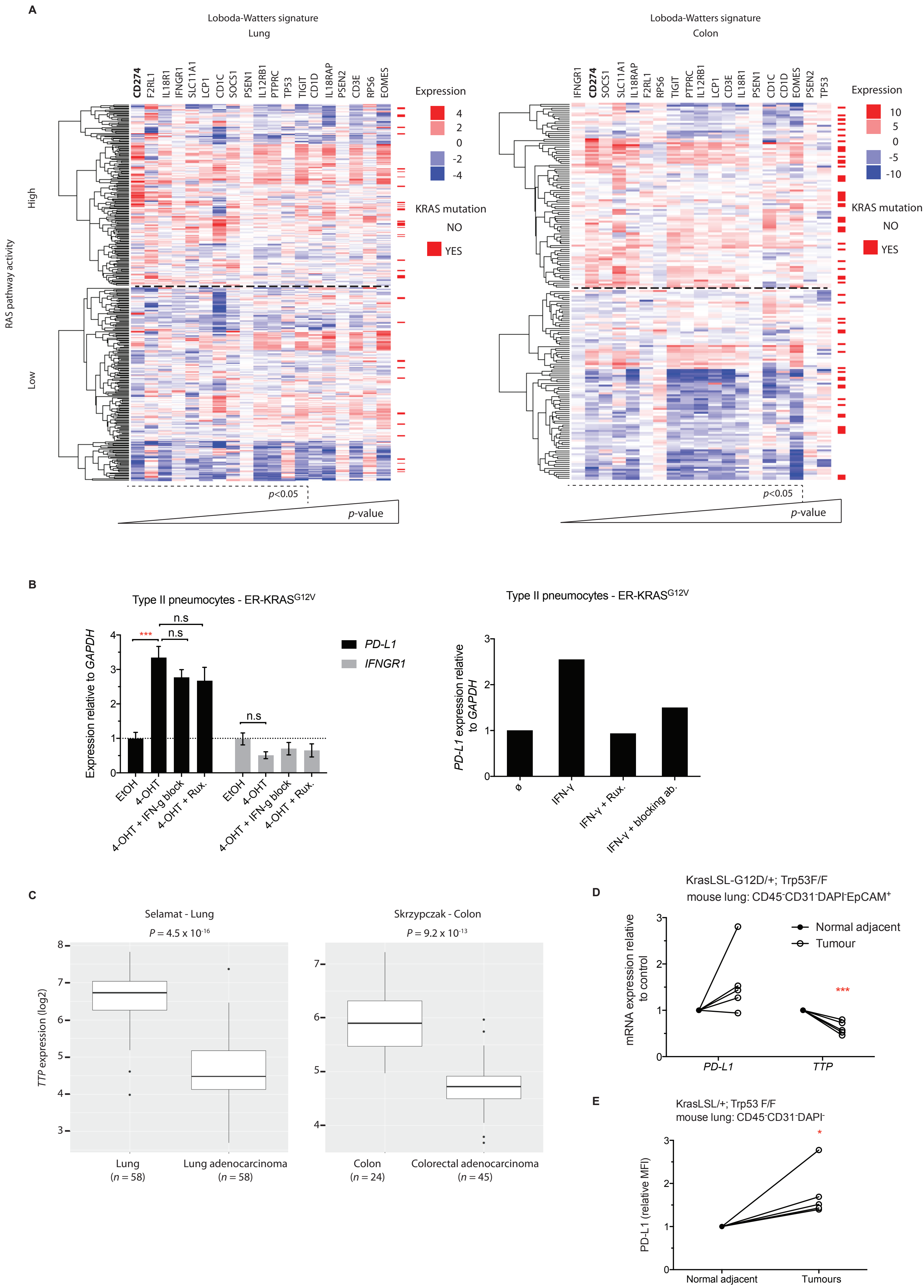
A
Loboda-Watters signature
Lung
CD274
F2RL1
IL18R1
IFNGR1
SLC11A1
LCP1
CD1C
SOCS1
PSEN1
IL12RB1
PTPRC
TP53
TIGIT
CD1D
IL18RAP
PSEN2
CD3E
RPS6
EOMES
Expression
4
2
0
-2
-4
KRAS mutation
NO
YES
RAS pathway activity
High
Low
p<0.05
p-value
Loboda-Watters signature
Colon
IFNGR1
CD274
SOCS1
SLC11A1
IL18RAP
F2RL1
RPS6
TIGIT
PTPRC
IL12RB1
LCP1
CD3E
IL18R1
PSEN1
CD1C
CD1D
EOMES
PSEN2
TP53
Expression
10
5
0
-5
-10
KRAS mutation
NO
YES
p<0.05
p-value
B
Type II pneumocytes - ER-KRASG12V
Expression relative to GAPDH
***
n.s
n.s
n.s
PD-L1
IFNGR1
EtOH
4-OHT
4-OHT + IFN-g block
4-OHT + Rux.
Type II pneumocytes - ER-KRASG12V
PD-L1 expression relative to GAPDH
ø
IFN-γ
IFN-γ + Rux.
IFN-γ + blocking ab.
C
Selamat - Lung
P = 4.5 x 10-16
TTP expression (log2)
Lung
(n = 58)
Lung adenocarcinoma
(n = 58)
Skrzypczak - Colon
P = 9.2 x 10-13
Colon
(n = 24)
Colorectal adenocarcinoma
(n = 45)
D
KrasLSL-G12D/+; Trp53F/F
mouse lung: CD45-CD31-DAPI-EpCAM+
mRNA expression relative to control
Normal adjacent
Tumour
***
PD-L1
TTP
E
KrasLSL/+; Trp53 F/F
mouse lung: CD45-CD31-DAPI-
PD-L1 (relative MFI)
*
Normal adjacent
Tumours

Figure S7

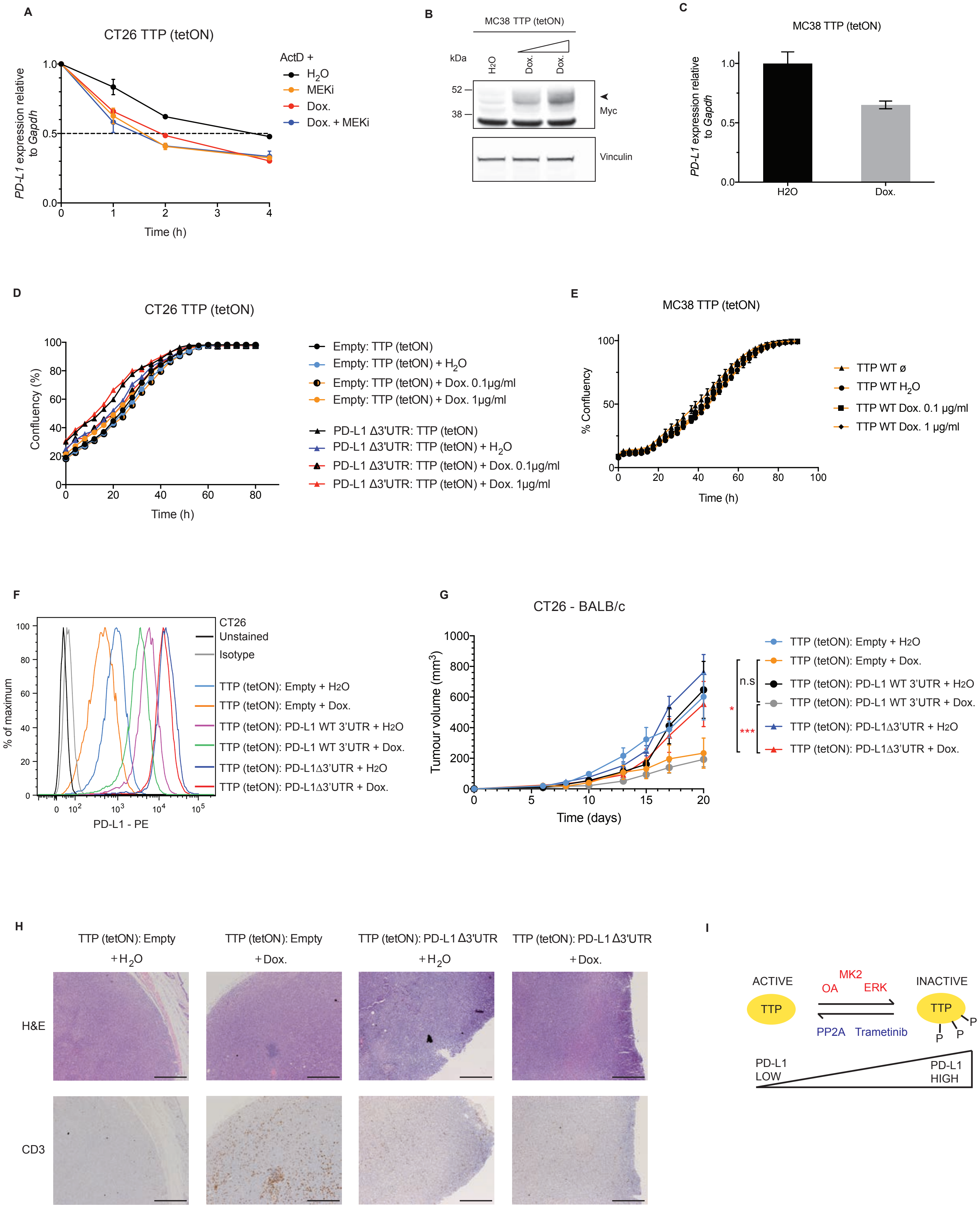
A
CT26 TTP (tetON)
PD-L1 expression relative to Gapdh
Time (h)
ActD +
H2O
MEKi
Dox.
Dox. + MEKi
B
MC38 TTP (tetON)
kDa
H2O
Dox.
Dox.
52
38
Myc
Vinculin
C
MC38 TTP (tetON)
PD-L1 expression relative to Gapdh
H2O
Dox.
D
CT26 TTP (tetON)
Confluency (%)
Time (h)
Empty: TTP (tetON)
Empty: TTP (tetON) + H2O
Empty: TTP (tetON) + Dox. 0.1µg/ml
Empty: TTP (tetON) + Dox. 1µg/ml
PD-L1 Δ3'UTR: TTP (tetON)
PD-L1 Δ3'UTR: TTP (tetON) + H2O
PD-L1 Δ3'UTR: TTP (tetON) + Dox. 0.1µg/ml
PD-L1 Δ3'UTR: TTP (tetON) + Dox. 1µg/ml
E
MC38 TTP (tetON)
% Confluency
Time (h)
TTP WT ø
TTP WT H2O
TTP WT Dox. 0.1 µg/ml
TTP WT Dox. 1 µg/ml
F
% of maximum
PD-L1 - PE
CT26
Unstained
Isotype
TTP (tetON): Empty + H2O
TTP (tetON): Empty + Dox.
TTP (tetON): PD-L1 WT 3'UTR + H2O
TTP (tetON): PD-L1 WT 3'UTR + Dox.
TTP (tetON): PD-L1Δ3'UTR + H2O
TTP (tetON): PD-L1Δ3'UTR + Dox.
G
CT26 - BALB/c
Tumour volume (mm3)
Time (days)
TTP (tetON): Empty + H2O
TTP (tetON): Empty + Dox.
TTP (tetON): PD-L1 WT 3'UTR + H2O
TTP (tetON): PD-L1 WT 3'UTR + Dox.
TTP (tetON): PD-L1Δ3'UTR + H2O
TTP (tetON): PD-L1Δ3'UTR + Dox.
n.s
*
***
H
TTP (tetON): Empty + H2O
TTP (tetON): Empty + Dox.
TTP (tetON): PD-L1 Δ3'UTR + H2O
TTP (tetON): PD-L1 Δ3'UTR + Dox.
H&E
CD3
I
ACTIVE
TTP
MK2
OA
ERK
INACTIVE
TTP
P
P
P
PP2A
Trametinib
PD-L1 LOW
PD-L1 HIGH
