# Supplementary material for: Oncogenic RAS Signaling Promotes Tumor Immunoresistance by Stabilizing PD-L1 mRNA
Source: Immunity. 2017 Dec 19;47(6):1083–1099.e6. doi: 10.1016/j.immuni.2017.11.016 (PMC5746170; doi:10.1016/j.immuni.2017.11.016)
Supplement: Document S2. Article plus Supplemental Information [file mmc2.pdf]

Immunity

Article

Oncogenic RAS Signaling Promotes Tumor Immunoresistance by Stabilizing PD-L1 mRNA

Graphical Abstract

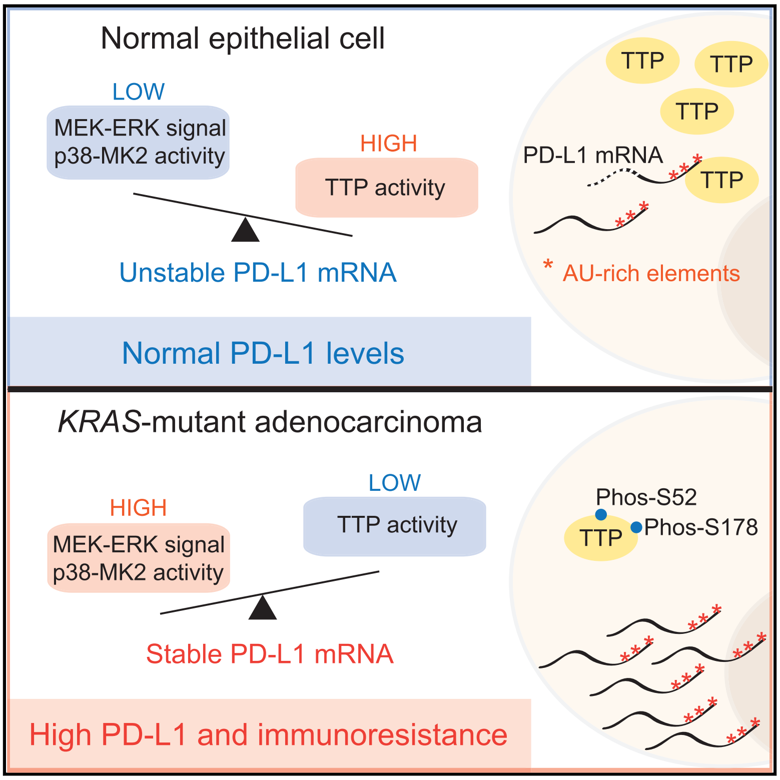

Authors

Matthew A. Coelho,
Sophie de Carné Trécesson,
Sareena Rana, ..., Wi S. Lai,
Perry J. Blackshear, Julian Downward

Correspondence

julian.downward@crick.ac.uk

In Brief

Coelho et al. demonstrate a post-transcriptional mechanism whereby oncogenic RAS signaling increases PD-L1 expression. Mechanistically, PD-L1 mRNA is targeted by TTP through AU-rich elements in the 3′ UTR, making it unstable. Oncogenic RAS signaling reduces TTP activity and stabilizes the PD-L1 transcript. Restoring TTP activity reduces PD-L1 expression and enhances anti-tumor immunity.

Highlights

- Oncogenic RAS signaling via MEK increases PD-L1 expression
- RAS regulates PD-L1 through AU-rich elements (AREs) in the 3′ UTR of PD-L1 mRNA
- The ARE-binding protein tristetraprolin (TTP) negatively regulates PD-L1 expression
- Restoration of tumor cell TTP activity enhances anti-tumor immunity

Coelho et al., 2017, Immunity *47*, 1083–1099
December 19, 2017 © 2017 The Author(s). Published by Elsevier Inc.
https://doi.org/10.1016/j.immuni.2017.11.016

Immunity
Article

CellPress

Oncogenic RAS Signaling Promotes Tumor Immunoresistance by Stabilizing PD-L1 mRNA

Matthew A. Coelho,[1] Sophie de Carné Trécesson,[1] Sareena Rana,[7] Davide Zecchin,[1] Christopher Moore,[1] Miriam Molina-Arcas,[1] Philip East,[2] Bradley Spencer-Dene,[3] Emma Nye,[3] Karin Barnouin,[4] Ambrosius P. Snijders,[4] Wi S. Lai,[5] Perry J. Blackshear,[5,6] and Julian Downward[1,7,8,*]
[1]Oncogene Biology
[2]Computational Biology
[3]Experimental Histopathology
[4]Protein Analysis and Proteomics Laboratories
The Francis Crick Institute, 1 Midland Road, London NW1 1AT, UK
[5]Signal Transduction Laboratory, National Institute of Environmental Health Sciences, Research Triangle Park, NC 27709, USA
[6]Departments of Medicine and Biochemistry, Duke University Medical Center, Durham, NC 27703, USA
[7]Lung Cancer Group, Division of Molecular Pathology, The Institute of Cancer Research, 237 Fulham Road, London SW3 6JB, UK
[8]Lead Contact
*Correspondence: julian.downward@crick.ac.uk
https://doi.org/10.1016/j.immuni.2017.11.016

SUMMARY

The immunosuppressive protein PD-L1 is upregulated in many cancers and contributes to evasion of the host immune system. The relative importance of the tumor microenvironment and cancer cell-intrinsic signaling in the regulation of PD-L1 expression remains unclear. We report that oncogenic RAS signaling can upregulate tumor cell PD-L1 expression through a mechanism involving increases in PD-L1 mRNA stability via modulation of the AU-rich element-binding protein tristetraprolin (TTP). TTP negatively regulates PD-L1 expression through AU-rich elements in the 3′ UTR of PD-L1 mRNA. MEK signaling downstream of RAS leads to phosphorylation and inhibition of TTP by the kinase MK2. In human lung and colorectal tumors, RAS pathway activation is associated with elevated PD-L1 expression. *In vivo*, restoration of TTP expression enhances anti-tumor immunity dependent on degradation of PD-L1 mRNA. We demonstrate that RAS can drive cell-intrinsic PD-L1 expression, thus presenting therapeutic opportunities to reverse the innately immunoresistant phenotype of *RAS* mutant cancers.

INTRODUCTION

Therapeutic antibodies blocking the coinhibitory PD-1 pathway by targeting PD-L1 (programmed death 1 ligand 1, also known as B7-H1 or CD274) or its receptor, PD-1, have caused striking regressions in several malignancies in which RAS mutations are frequent driver events, including non-small cell lung cancer (NSCLC) (Herbst et al., 2014; Topalian et al., 2012) and mismatch-repair-deficient colorectal cancer (Le et al., 2015). PD-L1 is critical for limiting autoimmune-related damage to normal tissues in the context of chronic inflammation but is also aberrantly upregulated on cancer cells in order to evade immune destruction (Pardoll, 2012). As anti-PD-1 pathway immunotherapies are effective in only a minority of cancer patients (Topalian et al., 2012), there is a great need for reliable biomarkers of patient response. To what degree tumor PD-L1 expression is prognostic of patient response to PD-1 pathway blockade remains contentious. Recent clinical trials of the anti-PD-1 antibody nivolumab report that tumor cell PD-L1 expression correlates with response to nivolumab in non-squamous but not the squamous subtype of NSCLC (Borghaei et al., 2015; Brahmer et al., 2015). Notably, non-squamous NSCLC patients with *KRAS* mutations benefited from nivolumab therapy in terms of overall survival, whereas *KRAS* wild-type patients did not (Borghaei et al., 2015). Response rate and progression-free survival was increased in NSCLC patients treated with pembrolizumab in cases where at least 50% of tumor cells were positive for PD-L1 (Garon et al., 2015). In this patient cohort, *KRAS* mutant tumors were more frequently PD-L1 positive than *KRAS* wild-type tumors.

The success of immune-checkpoint blockade is dependent on the immunogenicity of the tumor (Gubin et al., 2014; Linnemann et al., 2015; Rizvi et al., 2015), so one possible confounding factor in the use of tumor PD-L1 as a biomarker for response is the uncoupling of tumor PD-L1 expression from tumor immunogenicity. It is therefore critical to understand the signaling pathways that dictate tumor cell PD-L1 expression. The inflammatory cytokine IFN-γ is the best-characterized stimulus for PD-L1 expression, but several studies suggest that cell-intrinsic oncogenic signaling can also promote PD-L1 expression in cancer cells through epidermal growth factor receptor (EGFR), the transcription factor MYC, and the kinase AKT (Akbay et al., 2013; Casey et al., 2016; Parsa et al., 2007). Studies performed on melanoma (Jiang et al., 2013) and acute myeloid leukemia (Berthon et al., 2010) have indicated that MEK signaling is involved in upregulation of PD-L1 in some tumor cell lines, but the molecular basis of this regulation remains poorly defined.

Separately, genetic rearrangements in the 3′ UTR of *CD274* (encoding PD-L1) have been found in a multitude of different cancers at low frequency and are associated with massively

This is an open access article under the CC BY license (http://creativecommons.org/licenses/by/4.0/).

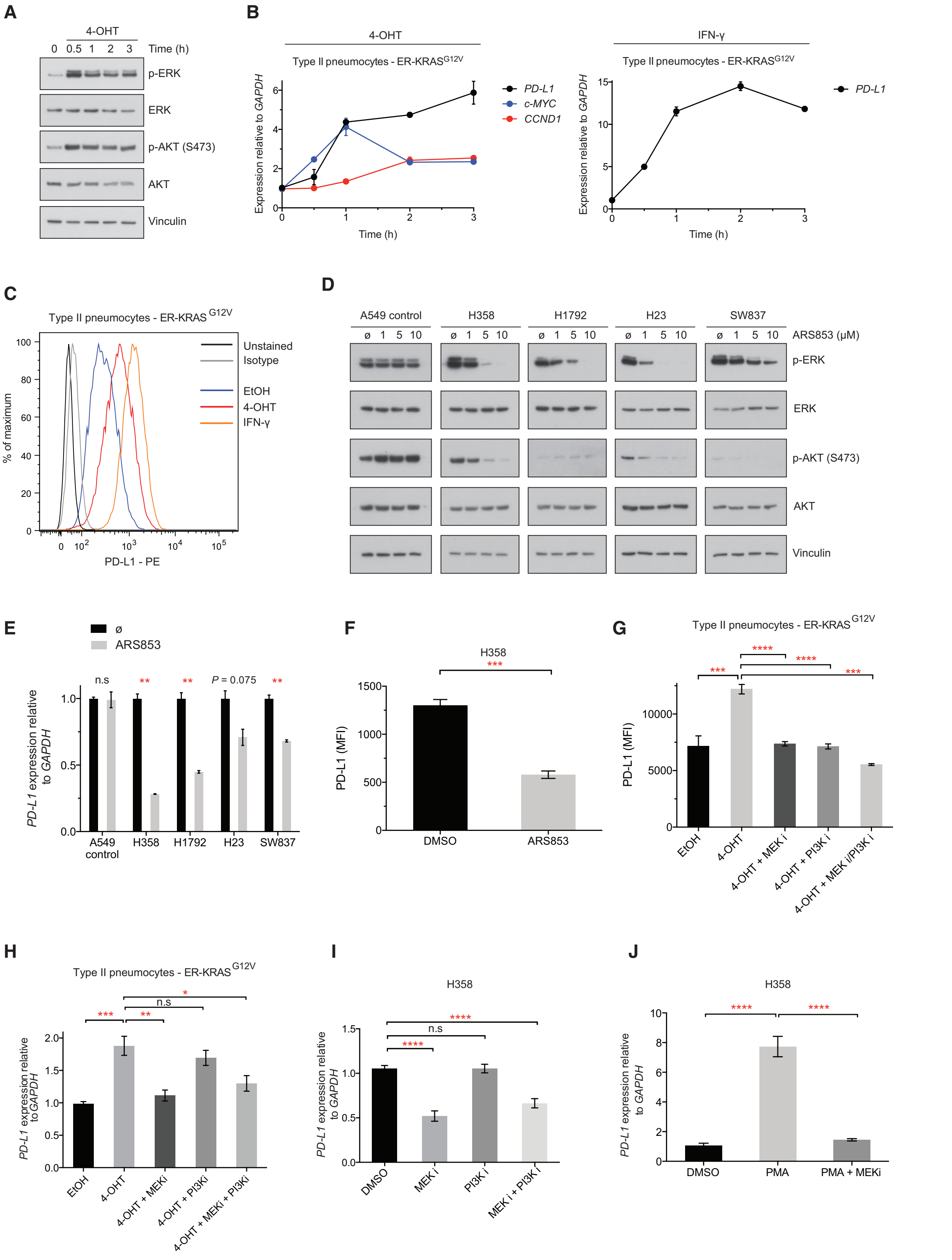

(legend on next page)

increased expression of tumor PD-L1 (Kataoka et al., 2016). These results imply that control of PD-L1 expression through the *CD274* 3′ UTR might contribute to immune escape in human cancers, although the underlying mechanisms of post-transcriptional regulation responsible for this effect are unclear.

In this report, we reveal that tumor cell PD-L1 expression can be driven by oncogenic RAS pathway activation by a mechanism involving post-transcriptional regulation of the stability of PD-L1 mRNA. This provides a direct mechanism whereby RAS signaling in tumor cells can provide protection from attack by the immune system.

RESULTS

Cell-Intrinsic Upregulation of PD-L1 through Oncogenic RAS Signaling

We tested the potential role of oncogenic RAS signaling in the regulation of PD-L1 expression in human epithelial cells using ER-RAS^{G12V} fusion constructs, which allow for the induction of oncogenic RAS activity with 4-hydroxytamoxifen (4-OHT) (Molina-Arcas et al., 2013). As expected, addition of 4-OHT led to the rapid activation of oncogenic KRAS signaling through MEK and PI3K (Figure 1A) and coincided with induction of MYC mRNA and CCND1 mRNA (encoding cyclin D1) in an immortalized human pneumocyte cell line derived from type II cells (Figure 1B; Kemp et al., 2008). PD-L1 mRNA was rapidly increased following stimulation of oncogenic KRAS signaling with 4-OHT, resulting in a 6-fold induction of mRNA expression after 3 hr (Figure 1B). By way of comparison with known regulators, stimulation with IFN-γ led to increases in PD-L1 mRNA in excess of 10-fold after 3 hr and both KRAS activation and IFN-γ stimulation dramatically increased PD-L1 protein expression at the cell surface after 48 hr (Figure 1C). Oncogenic HRAS signaling was also capable of inducing PD-L1 mRNA and protein expression in the immortalized breast epithelial cell line MCF10A and the *KRAS* wild-type colon carcinoma cell line HKE-3 (Figures S1A and S1B), implying that induction of PD-L1 expression by RAS is not a tissue-specific or RAS-isoform-specific phenomenon. The induction of PD-L1 protein was most striking in ER-HRAS^{G12V} MCF10A cells, perhaps reflecting the low basal expression of PD-L1. Chronic RAS activation for 4 days led to more profound increases in PD-L1 protein, whereas shorter-term activation resulted in modest inductions of PD-L1 expression (Figure S1B). Importantly, 4-OHT did not induce PD-L1 expression in parental cell lines lacking ER-RAS constructs (Figure S1C).

Direct inhibition of KRAS signaling with the KRAS^{G12C}-specific inhibitor ARS853 (Lito et al., 2016; Patricelli et al., 2016) in lung and colorectal cancer cell lines harboring KRAS^{G12C} mutations led to reductions in PD-L1 mRNA expression, but not in the KRAS^{G12S} A549 control lung cancer cell line (Figures 1D and 1E). Moreover, ARS853 treatment led to significant reductions in PD-L1 surface protein expression in the *KRAS* mutant lung cancer cell line H358 (Figure 1F). To dissect which downstream effectors of RAS are responsible for regulating PD-L1 expression, we used the specific inhibitors of MEK and pan type I PI3Ks, GSK1120212 (trametinib) and GDC-0941 (pictilisib), respectively (Figure S1D). Notably, MEK and PI3K inhibitors could block RAS-induced expression of PD-L1 protein in ER-KRAS^{G12V} type II pneumocytes, either alone or in combination (Figure 1G). MEK inhibition significantly reversed KRAS-mediated PD-L1 mRNA upregulation (Figure 1H), but PI3K inhibition only reduced PD-L1 protein expression, concordant with evidence for AKT signaling increasing PD-L1 expression predominantly through activating translation of the transcript (Parsa et al., 2007). MEK inhibition, but not PI3K inhibition, reduced PD-L1 mRNA expression in H358 (Figure 1I), H23, and H1792 lung cancer cell lines (Figure S1E). Downstream of MEK, inhibition of ERK1/2 with SCH772984 potently reduced PD-L1 expression in H358 and H23 cells (Figure S1F). Furthermore, PMA, a potent chemical activator of MEK-ERK signaling via protein kinase C stimulation, markedly and rapidly increased PD-L1 expression, an effect that was largely reversed with the inhibition of MEK (Figures 1J and S1G). More extensive analysis of PD-L1 surface expression on multiple *KRAS* mutant lung cancer cell lines, both human and murine, revealed generally consistent PD-L1 downregulation after MEK and PI3K inhibition, suggesting that this regulatory pathway is of broad significance

Figure 1. Cell-Intrinsic Upregulation of PD-L1 through Oncogenic RAS Signaling

(A) Western blotting analysis of ER-KRAS^{G12V} type II pneumocytes treated with 4-OHT in starvation medium. Phospho-ERK and phospho-AKT was measured over time to monitor RAS pathway activation. Data are representative of two independent experiments.

(B) qPCR analysis of ER-KRAS^{G12V} type II pneumocytes treated with 4-OHT or IFN-γ in starvation medium. Mean ± SEM of biological duplicates (n = 2) from the experiment described in (A).

(C) Representative flow cytometry histogram of PD-L1 surface protein expression in ER-KRAS^{G12V} type II pneumocytes treated in starvation medium for 48 hr. Data are representative of two independent experiments.

(D) Western blotting analysis of RAS signaling following 5 hr treatment with the KRAS^{G12C} inhibitor ARS853. Phospho-ERK and phospho-AKT signal reflect RAS pathway activity. Data are representative of two independent experiments.

(E) qPCR analysis following 5 hr treatment with the KRAS^{G12C} inhibitor ARS853 (10 μM). Mean ± SEM of biological duplicates (n = 2) from the experiment described in (D).

(F) Flow cytometry analysis of PD-L1 surface protein expression in H358 cells treated with ARS853 (10 μM) for 48 hr. Mean ± SEM of biological triplicates.

(G) Flow cytometry analysis of PD-L1 surface protein expression in ER-KRAS^{G12V} type II pneumocytes treated in starvation medium for 24 hr. Mean ± SEM of two independent experiments.

(H) qPCR analysis from the experiment described in (G). Mean ± SEM of biological triplicates pooled from two independent experiments.

(I) qPCR analysis of H358 cells treated for 24 hr. Mean ± SEM of two independent experiments.

(J) qPCR analysis of H358 cells treated with PMA for 3 hr following a 30 min pre-treatment with DMSO or MEK inhibitor. Mean ± SD of two independent experiments.

Abbreviations and quantities are as follows: MFI, mean fluorescence intensity; EtOH, ethanol vehicle; 4-OHT, 100 nM; IFN-γ, 20 ng/mL; MEK inhibitor GSK1120212, 25 nM; PI3K inhibitor GDC-0941, 500 nM; PMA, 200 nM. ****$p < 0.0001$, ***$p < 0.001$, **$p < 0.01$, *$p < 0.05$, n.s., not significant. Unpaired, two-tailed Student's t tests. See also Figure S1.

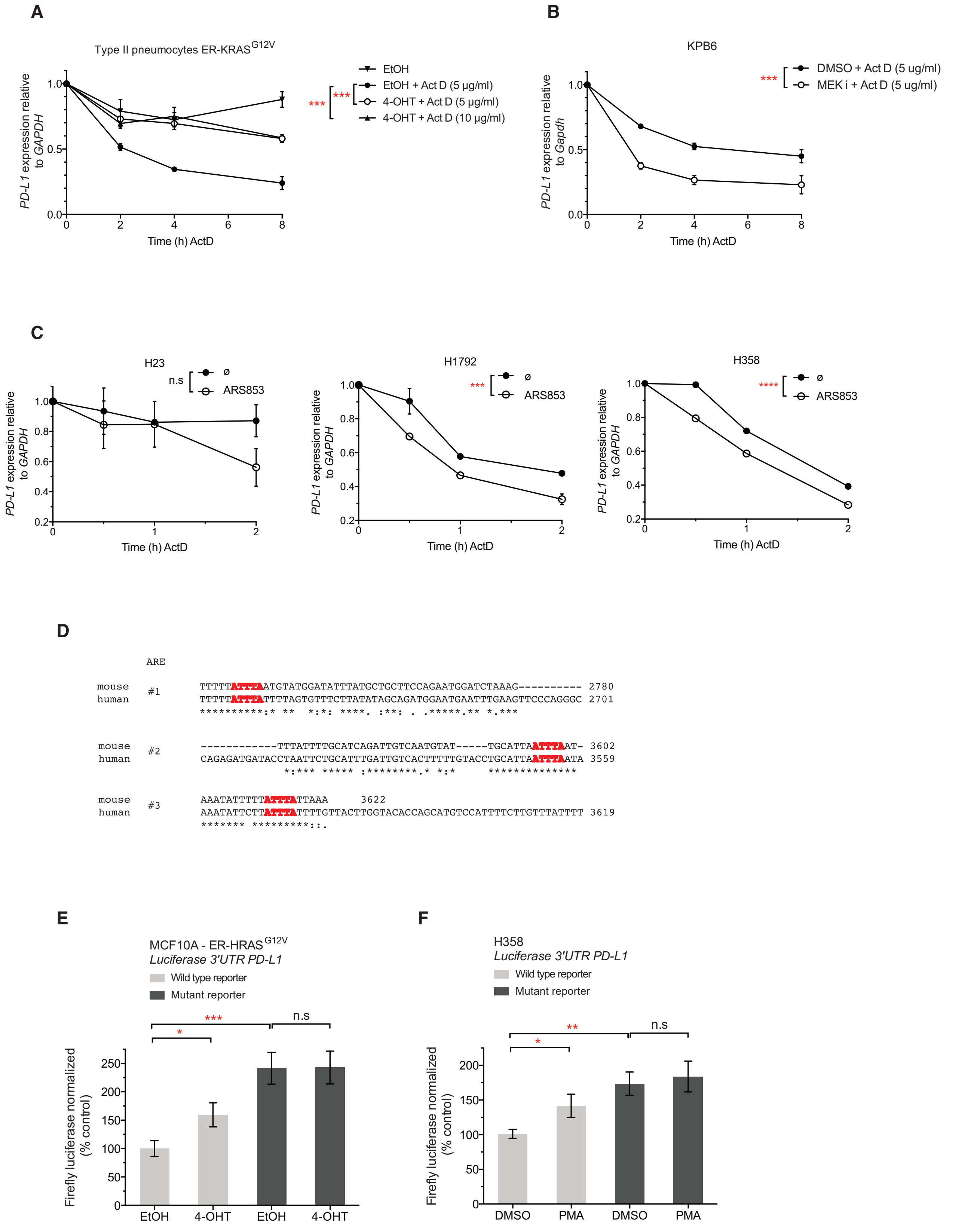

(legend on next page)

(Figure S1H). Taken together, these results suggest that oncogenic RAS signaling through MEK and PI3K is sufficient to drive PD-L1 expression.

Since RAS signaling has been implicated in reducing the expression of genes involved in the presentation of antigens by MHC class I molecules (Ebert et al., 2016; El-Jawhari et al., 2014), we analyzed the expression of antigen processing and antigen presentation machinery following oncogenic RAS activation (Figure S1I). As expected, KRAS G12V signaling led to significant decreases in expression of *TAP1*, *TAPBP*, as well as *HLA-A*, *HLA-B*, *HLA-C*, and *B2M*, suggesting that compromised antigen processing and presentation in concert with increases in PD-L1 expression may contribute to an augmented state of immunoresistance in *RAS* mutant tumor cells.

RAS Signaling Increases PD-L1 mRNA Stability through AU-Rich Elements in the 3′ UTR

To investigate how RAS-MEK signaling regulates PD-L1 expression, we first asked whether RAS regulates PD-L1 via a transcriptional mechanism. We generated a series of luciferase reporter constructs containing promoter fragments cloned from the human *CD274* locus (Figure S2A). In all cases, the physiological stimulus IFN-γ, but not PMA, induced expression of the promoter reporter constructs in H358 cells, a cell line in which endogenous PD-L1 mRNA expression is robustly induced with PMA (Figure 1J). Incorporation of putative enhancer elements (Sumimoto et al., 2016) into the *CD274* promoter reporter constructs also failed to confer sensitivity to MAPK activation (Figure S2A), as did including predicted regulatory regions spanning the 5′ of exon 1 (data not shown). Furthermore, none of the reporters showed evidence of decreased expression when H358 cells were treated with MEK inhibitor (data not shown).

Therefore, we investigated possible mechanisms of post-transcriptional regulation of PD-L1 expression by RAS. We induced oncogenic KRAS signaling with 4-OHT in ER-KRAS^{G12V} type II pneumocytes and concomitantly blocked transcription with actinomycin D. Surprisingly, we found human PD-L1 mRNA to have a short half-life, which was significantly stabilized by the induction of oncogenic KRAS signaling (Figure 2A). Moreover, murine PD-L1 mRNA also had a comparably short half-life, and the stability of the transcript in a *Kras* mutant, p53-deleted murine lung tumor cell line (KPB6), could be reduced further still when MEK was inhibited (Figure 2B), implicating KRAS-MEK signaling in the stabilization of the labile PD-L1 transcript. Consistently, direct inhibition of oncogenic KRAS signaling with ARS853 also caused reductions in PD-L1 mRNA half-life in H23, H1792, and H358 cells (Figure 2C). However, inhibition of PI3K alone did not result in altered PD-L1 mRNA stability in KPB6 cells (Figure S2B).

Common genetic elements conferring mRNA instability include miRNA binding sites and AU-rich elements (AREs) in the 3′ UTR of the transcript. The core motif for AREs is an ATTTA pentamer sequence, but functional AREs are often found in an AU-rich context, conforming to the WWATTTAWW nonamer consensus (where W denotes an A or T) (Zubiaga et al., 1995) constituting the binding site for several AU-rich element binding proteins (AUBPs), which can subsequently recruit mRNA decay machinery (Lykke-Andersen and Wagner, 2005). For example, a canonical ARE-regulated transcript is *TNF*, which contains nine pentamer sequences in the human transcript and eight pentamers in the murine transcript. Upon inspection of the 3′ UTR of PD-L1 mRNA, we noted a high number of ARE pentamers. Specifically, out of 14 ATTTA pentamer sequences in the human transcript and 11 in the murine transcript, there were 3 conserved AREs conforming to the nonamer consensus (Figure 2D).

We tested the influence of MEK inhibition on the half-life of another unstable transcript, Tusc2 mRNA (tumor suppressor candidate 2, or Fus1), which does not contain AU-rich elements in the 3′ UTR but is targeted by multiple miRNAs (Du et al., 2009). Although Tusc2 mRNA had a similar half-life to PD-L1 mRNA, MEK inhibition did not influence the stability of the Tusc2 transcript (Figure S2C), indicating that the observed post-transcriptional regulation of PD-L1 by MEK may relate to AU-rich elements in the 3′ UTR. Indeed, a transcript containing functional AU-rich elements, Ptgs2 mRNA (Cha et al., 2011), displayed a significant reduction in mRNA half-life in response to MEK inhibition (Figure S2C), reminiscent of PD-L1 mRNA.

To directly analyze the functional importance of these AREs, we constructed a luciferase reporter containing a fragment of the 3′ UTR of human *CD274* containing the last six ATTTA pentamers, including the three conserved nonamer sequences. Mutation of ATTTA pentamers to ATGTA has been shown to increase the expression of ARE-containing mRNAs (Rajagopalan et al., 1995; Yang et al., 2004). Consistent with this, mutating the six ATTTA pentamer sequences to ATGTA increased expression of the PD-L1 3′ UTR luciferase reporter in ER-HRAS^{G12V} MCF10A and H358 cells, suggesting that these AREs are functionally relevant for controlling the expression of PD-L1 (Figures 2E and 2F). Stimulation with 4-OHT in

Figure 2. RAS Signaling Increases PD-L1 mRNA Stability through AU-Rich Elements in the 3′ UTR

(A) qPCR analysis of PD-L1 mRNA stability in ER-KRAS^{G12V} type II pneumocytes after the concomitant addition of actinomycin D (5 μg/mL or 10 μg/mL) and 4-OHT or vehicle added at time = 0 hr in starvation medium. Mean ± SEM of two independent experiments. ***$p < 0.0005$; two-way ANOVA.

(B) qPCR analysis of PD-L1 mRNA stability in KPB6 cells after the addition of actinomycin D (5 μg/mL) and DMSO or MEK inhibitor. Cells were pre-treated with DMSO or MEK inhibitor for 30 min before actinomycin D addition. Mean ± SEM of two independent experiments. ***$p < 0.0005$; two-way ANOVA.

(C) qPCR analysis of PD-L1 mRNA stability after the addition of actinomycin D (5 μg/mL) and DMSO or ARS853. Cells were pre-treated with DMSO or ARS853 for 35 min before actinomycin D addition. Mean ± SEM of two independent experiments. ***$p < 0.0005$; two-way ANOVA.

(D) Sequence alignment of conserved AU-rich element ATTTA pentamer sequences (highlighted in red) in the mouse and human *CD274* 3′ UTR.

(E) Normalized luciferase signal in ER-HRAS^{G12V} MCF10A cells from wild-type (ATTTA x 6) or mutant (ATGTA x 6) PD-L1 3′ UTR reporters, 24 hr after treatment in starvation medium. Mean ± SEM of three independent experiments.

(F) Normalized luciferase signal in H358 cells from wild-type (ATTTA x 6) or mutant (ATGTA x 6) PD-L1 3′ UTR reporters, 6 hr after treatment. Mean ± SEM of three independent experiments.

Abbreviations and quantities: 4-OHT, 100 nM; MEK inhibitor GSK1120212, 25 nM; PMA, 200 nM. ***$p < 0.0005$, **$p < 0.005$, *$p < 0.05$, n.s., not significant. Unpaired, two-tailed Student's t tests. See also Figure S2.

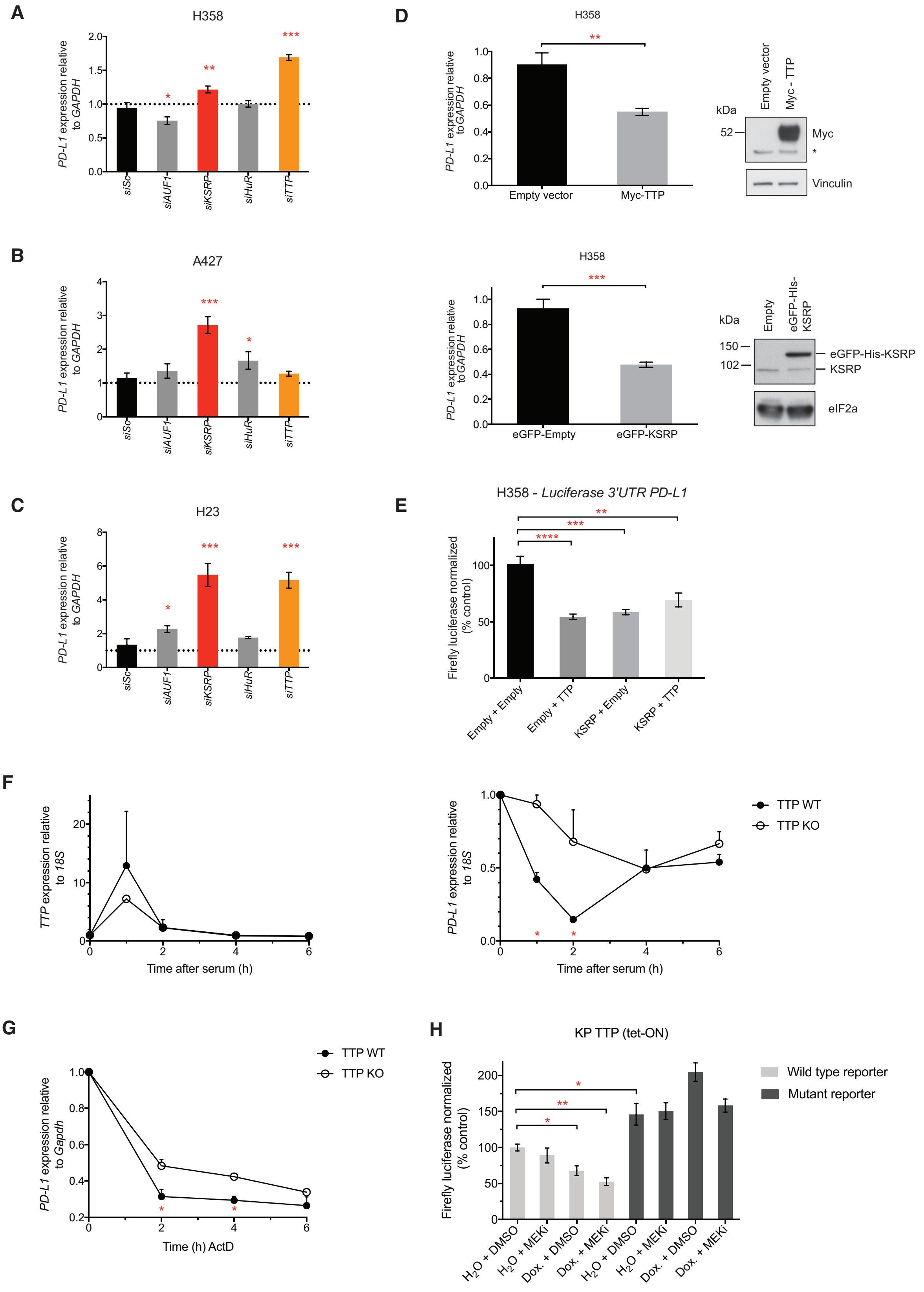

(legend on next page)

ER-HRAS^{G12V} MCF10A cells, or PMA in H358 cells, increased expression of the wild-type reporter, whereas the ATGTA mutant reporter was insensitive to these treatments (Figures 2E and 2F). In sum, these data suggest that AREs in the 3′ UTR of PD-L1 mRNA can mediate control of PD-L1 expression by RAS-MEK signaling.

AU-Rich Element Binding Proteins TTP and KSRP Are Negative Regulators of PD-L1 Expression

To assess which AU-rich element binding proteins (AUBPs) could mediate regulation of PD-L1 expression downstream of RAS signaling, we performed a selected siRNA screen of likely candidate genes, *AUF1*, *KSRP*, *HuR*, and *TTP* (also known as tristetraprolin or *ZFP36*), in three *RAS* mutant lung cancer cell lines (Figures 3A–3C). Knockdown efficiency was verified in each case by qPCR (Figures S3A–S3C). siRNA-mediated knockdown of KSRP and TTP most consistently increased PD-L1 mRNA expression across the cell line panel, with the exception of A427, where knock-down of TTP did not lead to significant increases in PD-L1 mRNA levels. Overexpression of KSRP or TTP was sufficient to significantly decrease PD-L1 expression (Figure 3D) and PD-L1 3′ UTR luciferase reporter expression in H358 cells (Figure 3E), corroborating our results from the siRNA screen and confirming that KSRP and TTP impart their negative regulation of PD-L1 expression through the 3′ UTR. Overexpression of TTP and KSRP together did not result in additive reductions in PD-L1 expression, suggesting that they may regulate PD-L1 through the same mechanism (Figure S3D). Notably, siRNA-mediated knockdown of TTP family members, BRF-1 and BRF-2, was incapable of increasing PD-L1 expression to the extent achieved by silencing TTP expression (Figures S3E and S3F). We confirmed that TTP protein expression was reduced following knock-down in H23 and H358 cells, but this was less clear in A427 cells, which express lower levels of TTP protein (Figure S3G). Deconvolution of siRNA pools targeting TTP showed that multiple siRNAs increased expression of PD-L1 mRNA in H23 and H358 cells (Figure S3H).

We further examined the regulation of PD-L1 mRNA by TTP by using TTP wild-type (WT) and TTP knock-out (KO) MEFs. In the TTP KO MEFs, TTP mRNA is expressed but no functional TTP protein can be made due to the introduction of a premature stop codon at the endogenous locus (Lai et al., 2006; Taylor et al., 1996). Acute activation of TTP expression with serum temporally coincided with a substantial and transient decrease in PD-L1 mRNA in TTP WT MEFs, but not in the TTP KO MEFs (Figure 3F), with PD-L1 levels recovering to near baseline at 6 hr after serum addition. Moreover, the total absence of functional TTP protein in the TTP KO MEFs increased the half-life of PD-L1 mRNA relative to TTP WT MEFs (Figure 3G).

Finally, we generated a KPB6 lung cancer cell line with a tetracycline-inducible TTP transgene (TTP tet-ON). As expected, inducible expression of TTP led to reductions in wild-type PD-L1 3′ UTR luciferase reporter expression, but not of the ATGTA mutant 3′ UTR reporter (Figure 3H). When combined with MEK inhibition, TTP expression more robustly suppressed expression of the wild-type reporter. In sum, these data provide evidence for the negative regulation of PD-L1 mRNA expression by the AUBPs KSRP and TTP.

RAS Regulates PD-L1 Expression through TTP

To further investigate whether MEK and TTP regulate PD-L1 via a shared pathway, we silenced TTP expression using siRNAs in the context of MEK inhibition. Knock-down of TTP was largely able to rescue the decrease in PD-L1 expression caused by MEK inhibition (Figure 4A). However, the knockdown of KSRP could not rescue this phenotype, despite profound silencing of expression (Figure S4A). Furthermore, MEK inhibition significantly increased TTP mRNA expression (Figure 4A), and chronic activation of oncogenic KRAS signaling significantly decreased TTP mRNA expression (Figure 4B).

Next, we tested whether the RAS pathway regulates the activity of TTP and/or KSRP protein. Crucially, we found that endogenous levels of TTP and KSRP both co-precipitated with PD-L1 mRNA in RNA immunoprecipitation (RNA-IP) reactions from KPB6 mouse lung cancer cells (Figure S4B). TTP also significantly bound to PD-L1 mRNA in H358 cells (Figure 4C). In all cases, the enrichment for the PD-L1 transcript was far greater than that of a control mRNA, GAPDH, which lacks AREs in the 3′ UTR (Figures 4C and S4C). MEK inhibition did not significantly alter the occupancy of TTP or KSRP on PD-L1 mRNA, consistent with RAS regulating the activity of the AUBP, rather than the occupancy on the target mRNA.

ERK has been shown to phosphorylate (Taylor et al., 1995) and negatively regulate TTP activity and expression (Bourcier et al., 2011; Deleault et al., 2008; Essafi-Benkhadir et al., 2007; Härdle et al., 2015). Inhibition of MEK decreased phosphorylation of TTP at PXSP (ERK target-site consensus) and RXXS/T (RSK/AKT target-site consensus) motifs (Figures 4D and 4E), confirming that TTP is regulated by phosphorylation downstream of MEK signaling in cancer cells. Mutation of two of the highest

Figure 3. AU-Rich Element Binding Proteins TTP and KSRP Are Negative Regulators of PD-L1 Expression

(A–C) qPCR analysis 48 hr after transfection with siRNAs targeting AU-rich element binding proteins (AU-BPs) relative to siScrambled (siSc) control. Mean ± SD of biological triplicates.

(D) qPCR and western blotting analysis of H358 cells 24 hr after transfection. qPCR data represent the mean ± SD of biological triplicates and are representative of two independent experiments. *, non-specific band.

(E) Normalized luciferase signal from the wild-type, PD-L1 3′ UTR reporter 24 hr after co-transfection with the indicated constructs. Mean ± SEM of two independent experiments.

(F) qPCR analysis after serum stimulation in serum-starved TTP WT or TTP KO MEFs. Mean ± SEM of two independent experiments.

(G) qPCR analysis of PD-L1 mRNA stability after the addition of actinomycin D (5 μg/mL) in TTP WT or TTP KO MEFs. Mean ± SEM of two independent experiments.

(H) Normalized luciferase signal in KPB6 TTP (tet-ON) cells wild-type (ATTTA x 6) or mutant (ATGTA x 6) PD-L1 3′ UTR reporters, 7 hr after treatment. Data represent the mean ± SEM of biological triplicates and are representative of two independent experiments.

Abbreviations and quantities: MEK inhibitor, GSK1120212, 25 nM; Dox., doxycycline 1 μg/mL. ****$p < 0.0001$, ***$p < 0.001$, **$p < 0.01$. Unpaired, two-tailed Student's t tests. See also Figure S3.

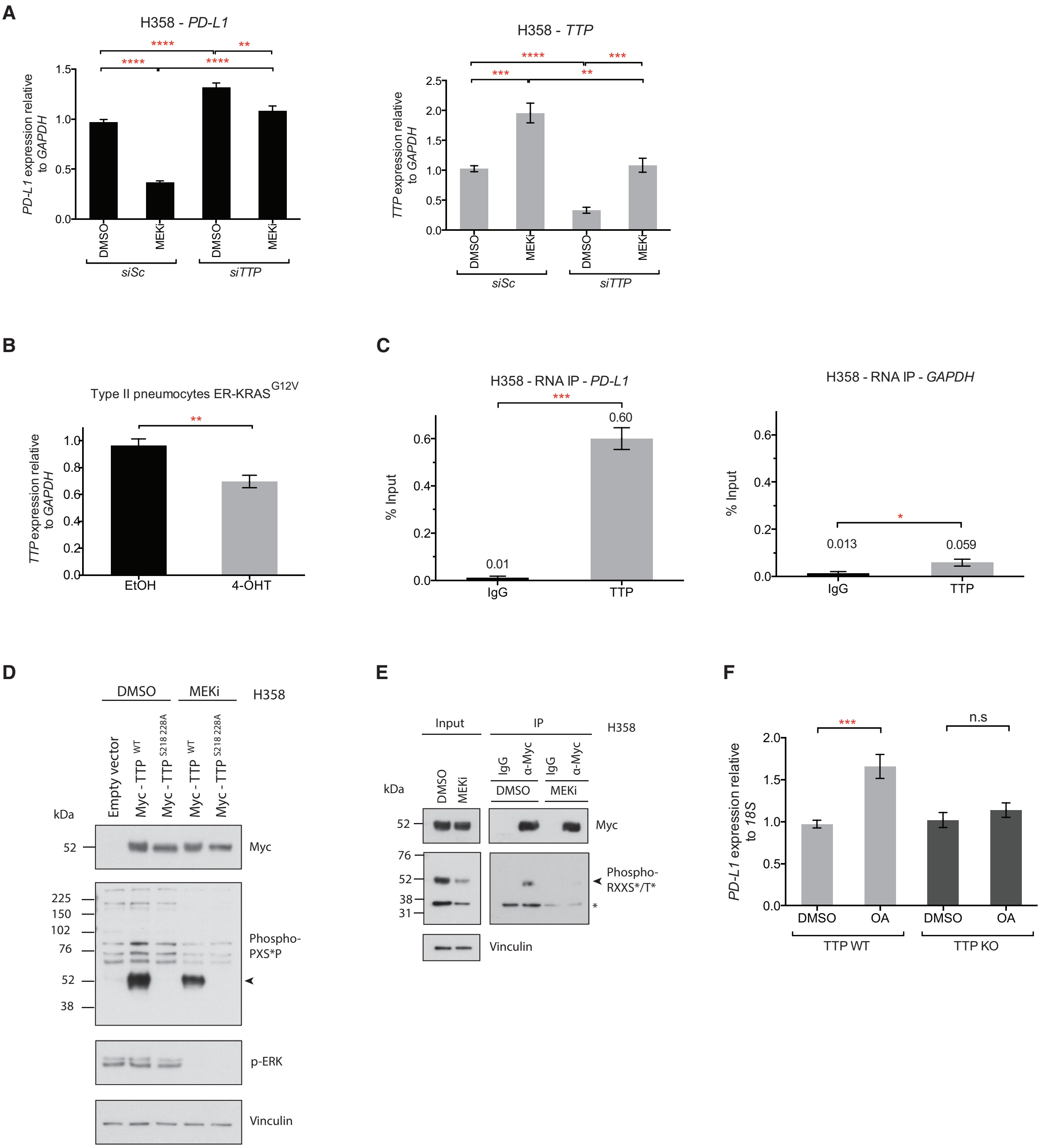

Figure 4. RAS Regulates PD-L1 Expression through TTP

(A) qPCR analysis of H358 cells following siRNA-mediated knock-down of TTP (24 hr) followed by MEK inhibition (24 hr). Mean ± SEM of two independent experiments.

(B) qPCR analysis of ER-KRAS^{G12V} type II pneumocytes treated for 24 hr in starvation medium. Mean ± SEM of three independent experiments.

(C) qPCR analysis of RNA-IP immunoprecipitates from H358 cells. Mean ± SEM from biological triplicates.

(D) Western blotting analysis of H358 cells expressing the indicated constructs. 6.5 hr post-transfection, cells were treated with DMSO or MEK inhibitor for an additional 16 hr. Arrow indicates Myc-TTP. Data are representative of two independent experiments.

(E) Western blotting analysis of immunoprecipitations from H358 cells transfected with Myc-TTP. 6.5 hr post-transfection, cells were treated with DMSO or MEK inhibitor for an additional 16 hr. Arrow indicates Myc-TTP; * indicates co-precipitating protein. Data are representative of two independent experiments.

(F) qPCR analysis of TTP WT or TTP KO MEFs treated with okadaic acid or DMSO for 2 hr. Mean ± SEM of two independent experiments.

Abbreviations and quantities: EtOH, ethanol vehicle; 4-OHT, 100 nM; okadaic acid, OA, 1 μM; MEK inhibitor, GSK1120212, 25 nM. ****$p < 0.0001$, ***$p < 0.001$, **$p < 0.01$. Unpaired, two-tailed Student's t tests. See also Figure S4.

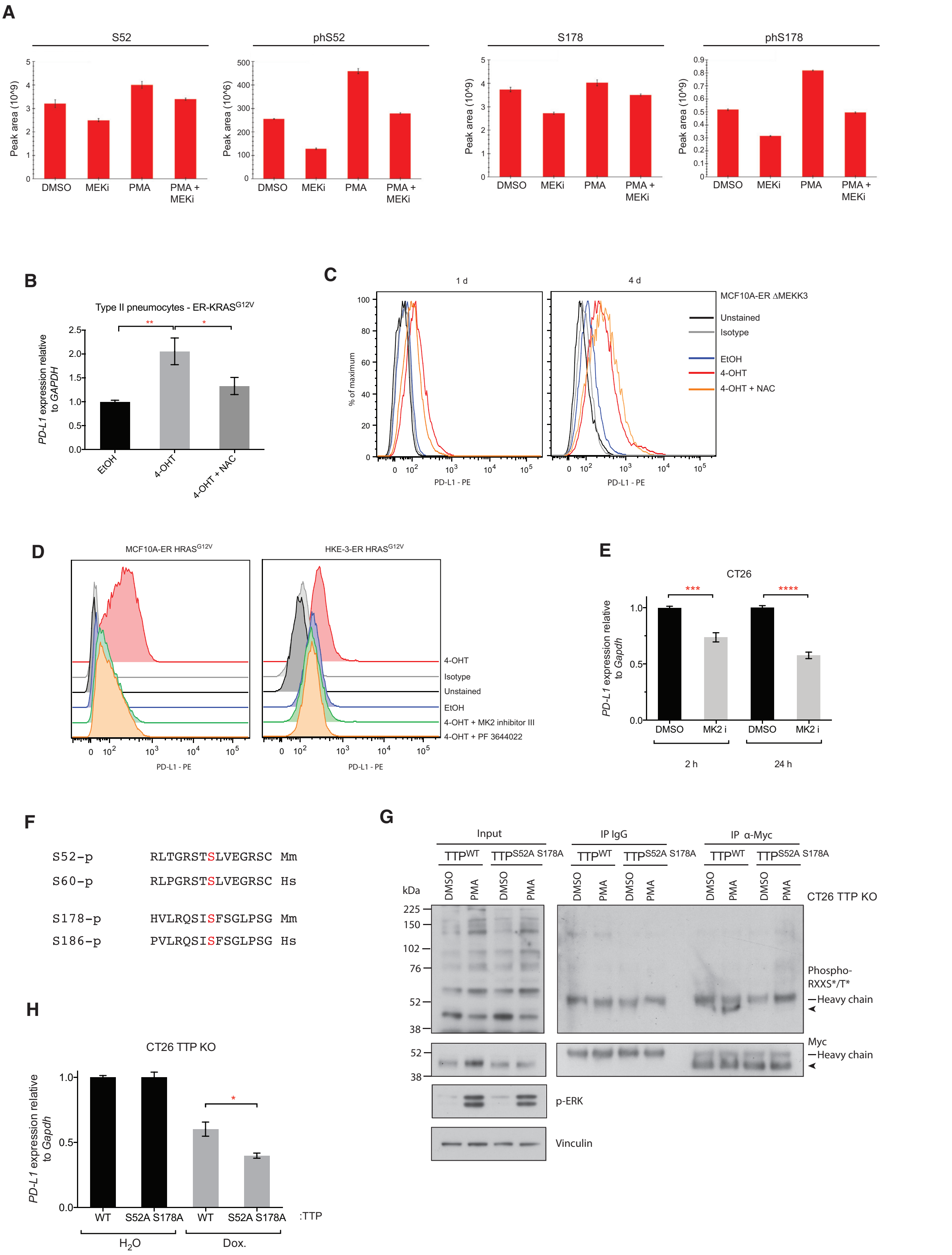

(legend on next page)

confidence predicted ERK-target residues on human TTP (S218 and S228) abrogated detection of TTP with the phospho-PXSP motif-specific antibody (Figure 4D), but the phosphosite mutant TTP (S218A 228A) did not show enhanced activity in reducing PD-L1 mRNA expression compared to wild-type TTP (data not shown), implying the involvement of other residues that are not readily detected with this antibody. Furthermore, although AKT signaling has been shown to regulate KSRP activity through phosphorylation of S193 (Díaz-Moreno et al., 2009), the KSRP S193A phosphosite mutant did not show enhanced activity in reducing PD-L1 mRNA expression compared to wild-type KSRP (Figure S4D).

Equally, the serine/threonine phosphatase PP2A has been implicated in positively regulating TTP function by reversing inhibitory phosphorylation events (Sun et al., 2007). Therefore, we tested whether inhibition of PP2A with okadaic acid (OA) would increase PD-L1 expression. OA rapidly increased PD-L1 mRNA expression in TTP WT MEFs, but not TTP KO MEFs (Figure 4F), demonstrating that PP2A activity decreases PD-L1 expression specifically through modulating TTP activity.

RAS-ROS-p38 Signaling Controls TTP Activity

To discover which residues are functionally important for regulating TTP activity downstream of RAS, we performed mass spectrometry on immunoprecipitated Myc-TTP after PMA, MEK inhibitor, or PMA and MEK inhibitor treatment. We used the *Kras* mutant, mouse colon carcinoma cell line CT26, based on its immunogenicity and sensitivity to anti-PD-L1 antibody therapy, making it suitable for downstream *in vivo* experiments. Most notably, mass spectrometry analysis revealed MEK-dependent phosphorylation of S52 and S178; PMA significantly enhanced phosphorylation of these residues, and this effect was reversed with MEK inhibition (Figures 5A and S5A and Table S1). Moreover, MEK inhibition alone was sufficient to reduce phosphorylation of these residues (Figure 5A).

S52 and S178 residues are crucial for the regulation of TTP activity through binding to 14-3-3 proteins following phosphorylation by MK2 (also known as MAPKAPK2) downstream of p38 (Chrestensen et al., 2004). Consequently, p38 signaling results in decreased TTP activity, partly through reducing the association with deadenylase machinery (Mahtani et al., 2001; Stoecklin et al., 2004). In parallel, phosphorylation of S52 and S178 stabilizes TTP protein (Brook et al., 2006), which is consistent with the observed increase in abundance of total TTP peptides detected in the PMA versus the MEK inhibitor-treated condition (Figure 5A).

We reasoned that oncogenic RAS might stimulate p38 signaling through promoting the MEK-dependent accumulation of reactive oxygen species (ROS) (Nicke et al., 2005) and thus inhibit TTP function. Indeed, oncogenic RAS signaling dramatically increased intracellular ROS in MCF10A cells, and ROS levels were distinctly correlated with the extent of PD-L1 induction (Figure S5B). Furthermore, the addition of the potent antioxidant N-acetyl-L-cysteine (NAC) largely reversed the induction of PD-L1 protein by RAS (Figures 5B and S5B), collectively suggesting that ROS induction by oncogenic RAS is functionally important in driving PD-L1 expression.

Specific activation of the p38 pathway using an inducible version of the upstream kinase MEKK3 (ΔMEKK3-ER) (Figures 5C and S5C; Garner et al., 2002) was sufficient to increase PD-L1 protein expression, albeit to a lesser extent than that achieved by RAS itself. Co-treatment with NAC was considerably less effective in reversing PD-L1 induction in this context, consistent with ROS operating upstream of p38 in this pathway (Figure 5C). Moreover, inhibition of MK2 strongly reversed RAS-induced PD-L1 expression in MCF10A and HKE-3 cells (Figure 5D) and PD-L1 expression in CT26 cells, which have endogenous levels of mutant KRAS (Figure 5E). We also observed reductions in expression of PD-L1 mRNA in several NSCLC cell lines with endogenous *KRAS* mutations following treatment with NAC, reduced glutathione, or MK2 inhibitor III (Figure S5D), although we noted some heterogeneity in response between the four cell lines tested.

To directly test the functional significance of the MK2 target residues downstream of MEK pathway activation, we generated TTP knock-out CT26 cell lines using CRISPR/Cas (to obviate functional contributions from endogenous TTP) and reconstituted these cells with either a wild-type (WT) or phosphosite mutant (S52A S178A), tetracycline-inducible TTP transgene. S52 and S178 of mouse TTP are highly conserved, with S52 conforming to the RXXS/T phosphosite motif (Figure 5F). Immunoprecipitation of Myc-tagged TTP following acute MAPK activation with PMA revealed phosphorylation of WT TTP, but not of the S52A S178A mutant protein at RXXS/T sites (Figure 5G),

Figure 5. RAS-ROS-p38 Signaling Controls TTP Activity

(A) Histograms represent peak areas from extracted ion chromatograms for non-phosphorylated and phosphorylated peptides corresponding to S52 and S178 phosphosites of mouse TTP. Myc-TTP was immunoprecipitated from CT26 Myc-TTP (tet-ON) cells 1 hr after the indicated treatment. Mean ± SD of technical triplicates. Representative of two independent biological experiments.

(B) qPCR analysis of ER-KRAS^{G12V} type II pneumocytes treated in starvation medium for 24 hr. Mean ± SEM of four independent experiments.

(C) Representative flow cytometry histograms of PD-L1 surface protein expression in MCF10A ER-ΔMEKK3 cells treated in starvation medium for 1 day or 4 days. Data are representative of two independent experiments.

(D) Flow cytometry analysis of PD-L1 surface protein expression on ER-HRAS^{G12V} MCF10A cells (24 hr) and ER-HRAS^{G12V} HKE-3 cells (48 hr) after treatment in starvation medium. Data are representative of biological duplicates.

(E) qPCR analysis of CT26 cells at 2 hr or 24 hr after MK2 inhibition with PF 3644022. Mean ± SEM of two independent experiments.

(F) Sequence alignments of the conserved phosphosites (highlighted red) targeted by MK2 in mouse (*Mm*) and human (*Hs*) TTP protein.

(G) Western blotting of immunoprecipitations from CT26 TTP KO cells harboring tet-ON, WT, or phospho mutant, Myc-TTP constructs. Cells were treated with dox. for 24 hr before the addition of PMA or DMSO for 1 hr. Arrow indicates Myc-TTP. Data are representative of two independent experiments.

(H) qPCR analysis of CT26 TTP KO cells harboring tet-ON, WT, or phospho mutant, Myc-TTP constructs, treated with dox or vehicle for 48 hr. Data represent the mean ± SEM of two independent experiments.

**$p < 0.005$, *$p < 0.05$. Unpaired, two-tailed Student's t test. Abbreviations and quantities: 4-OHT, 100 nM; NAC, N-acetyl-L-cysteine, 10 mM; PMA, 200 nM; MEK inhibitor, GSK1120212, 25 nM; MK2 inhibitor PF 3644022, 1 μM; MK2 inhibitor III, 1 μM; dox., doxycycline, 1 μg/mL. See also Figure S5.

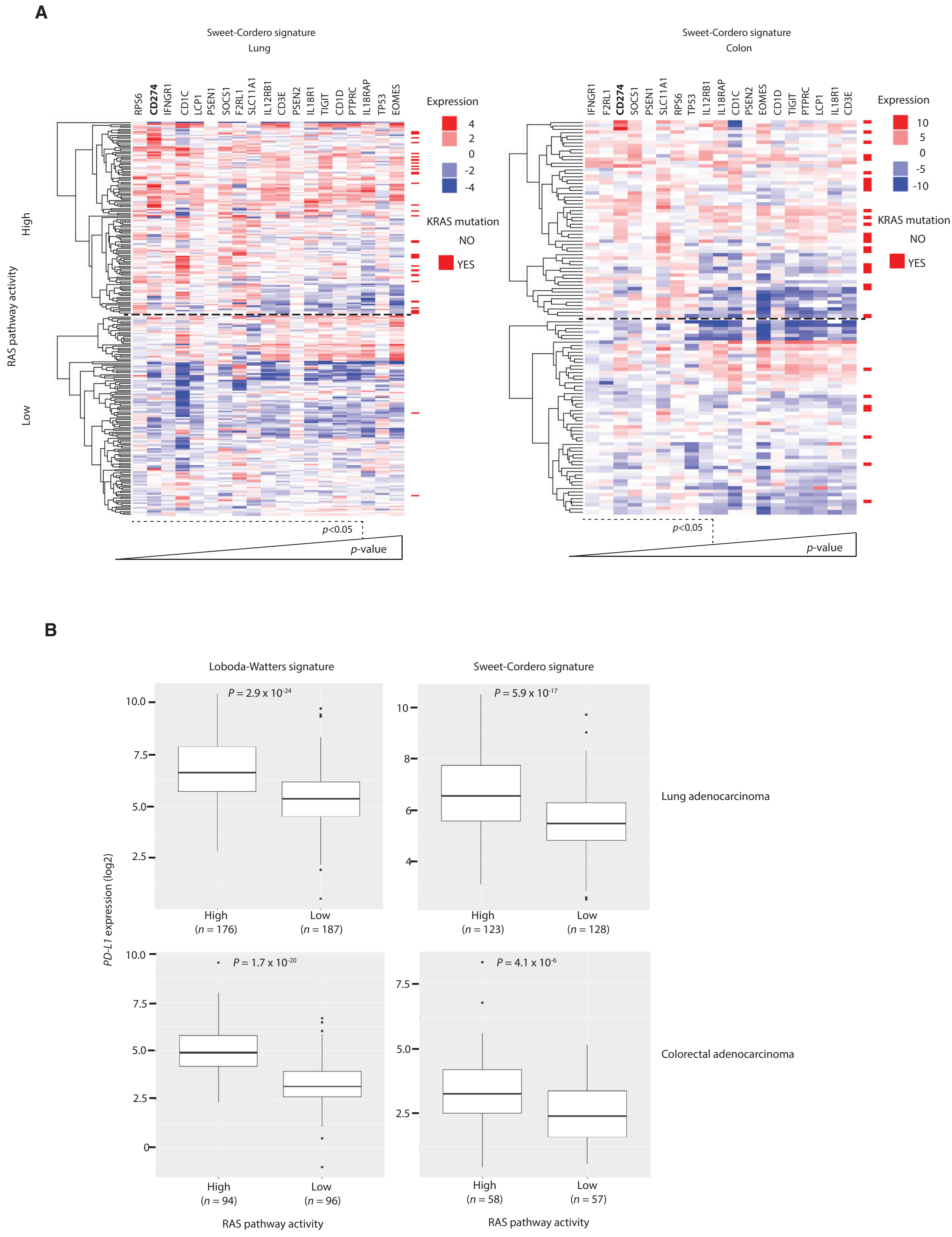

(legend on next page)

verifying our findings from mass spectrometry analysis. Crucially, the S52A S178A mutant TTP had significantly enhanced activity in reducing PD-L1 mRNA expression relative to WT TTP (Figures 5H and S5E). In sum, these results suggest that a RAS-ROS-p38 signaling axis contributes to PD-L1 upregulation through phosphorylation and inactivation of TTP.

RAS Pathway Activation Is Associated with PD-L1 Upregulation in Human Cancers

To further evaluate the role of oncogenic RAS signaling in regulating PD-L1 expression in cancer, we analyzed TCGA gene expression data from patient-derived lung adenocarcinoma (LUAD) or colon adenocarcinoma (COAD) samples. To account for the effects of alternative oncogenes that can activate downstream RAS effector pathways such as EGFR, BRAF, and ALK, we used two published gene expression signatures for RAS activation (Loboda et al., 2010; Sweet-Cordero et al., 2005) to segregate patient samples into "high" and "low" RAS pathway activity based on gene expression. As expected, annotation of *KRAS* mutation status revealed a strong enrichment for *KRAS* mutant samples in the high RAS activity cohorts in both signatures (Figures 6A and S6A). We compared the expression of T cell function-related genes between high and low RAS activity cohorts and found *CD274* (encoding PD-L1) expression to be significantly increased in the high RAS pathway activity samples in LUAD (1.42 log2-fold change) and COAD (1.17 log2-fold change) samples, using either signature (Figures 6A, 6B, and S6A). Stromal PD-L1 and tumor PD-L1 expression appear to have independent, suppressive effects on anti-tumor immunity (Lau et al., 2017), but we noted that the expression of the pan-leukocyte marker *PTPRC* (coding for CD45) and lymphocyte marker *CD3E* were only modestly increased in the high RAS pathway activity cohort, indicating that the differential in PD-L1 expression is not likely to be solely attributable to a higher degree of leukocyte infiltration in the tumor microenvironment (Figure 6A).

Of note, *IFNGR1* was also among the most significantly enriched transcripts in the high RAS pathway activity groups. To investigate the possibility that PD-L1 may be upregulated in RAS active tumors due to regulation by IFNGR1, we induced PD-L1 expression with RAS in ER-KRAS^{G12V} type II pneumocytes and concomitantly blocked IFNGR1 signaling using a depleting antibody for IFN-γ or with the JAK1/2 inhibitor ruxolitinib. Although both treatments effectively reduced responses to exogenous IFN-γ, PD-L1 induction by RAS was unaffected, suggesting independence from IFN-γ-IFNGR1 signaling (Figure 6SB).

To further explore the *in vivo* relevance of TTP regulation in human cancer, we compared TTP mRNA expression in normal tissue and tumor samples by using publically available datasets. TTP mRNA was strikingly downregulated in human lung and colon tumor samples compared to normal tissue (Figure S6C; Selamat et al., 2012; Skrzypczak et al., 2010), confirming that aberrant regulation of TTP expression is relevant in the human disease. Consistently, in FACS-sorted epithelial cells isolated from normal lung or matched tumor tissue from *Kras*$^{LSL-G12D/+}$; *Trp53*$^{F/F}$ (KP) mice, TTP mRNA expression was reduced in lung tumor tissue (Figure S6D). PD-L1 mRNA expression was generally higher in tumor tissue than in normal lung but not significantly increased; however, PD-L1 protein expression was significantly elevated, perhaps reflecting the contribution from AKT in promoting PD-L1 protein expression (Figure S6E).

Restoration of Tumor Cell TTP Expression Enhances Anti-tumor Immunity

Next, we set out to directly assess the functional importance of the regulation of PD-L1 expression by TTP in tumor progression. To this end, we generated a series of stable CT26 cell lines expressing Myc-tagged mouse TTP under a tetracycline-inducible promoter (TTP tet-ON), and in addition, constitutively expressing either empty vector or mouse *Cd274* cDNA lacking the 3′ UTR (PD-L1 Δ3′ UTR). TTP expression was induced upon addition of doxycycline in a dose-dependent manner (Figure 7A), resulting in decreased PD-L1 protein expression at the cell surface (Figure 7B). Overexpression of PD-L1 Δ3′ UTR rendered total PD-L1 levels effectively insensitive to TTP induction (Figure 7B). TTP transgene expression with doxycycline was also associated with a decrease in PD-L1 mRNA stability, which was comparable to that mediated by MEK inhibition in this system (Figure S7A).

To independently verify our findings in another cell line, we used MC38 tumor cells because they are known to exhibit sensitivity to PD-L1 modulation *in vivo* and show RAS pathway activation (Giannou et al., 2017). As expected, TTP was induced with doxycycline in MC38 (tet-ON) cells, leading to reductions in PD-L1 expression (Figures S7B and S7C).

Using these engineered cell lines, we performed subcutaneous transplantation experiments in mice and monitored tumor progression. Notably, the growth rates of the stable cell lines *in vitro* did not significantly differ with the overexpression of PD-L1 Δ3′ UTR cDNA or the induction of TTP transgene expression with doxycycline (Figure S7D and S7E). However, *in vivo*, doxycycline treatment significantly reduced CT26 and MC38 tumor growth in immune-competent, syngeneic mice (Figures 7C and 7D). Strikingly, the anti-tumor effects mediated by doxycycline treatment were absent in immunocompromised *nu/nu* mice harboring CT26 tumors (Figure 7E) and in mice treated with depleting antibodies against CD8 and CD4, implying an essential contribution from the adaptive immune system to this anti-tumor response (Figure 7F). CT26 tumor cells overexpressing PD-L1 Δ3′ UTR grew faster than the empty vector cells in BALB/c mice but had no growth advantage in *nu/nu* mice. Moreover, expression of PD-L1 Δ3′ UTR was able to rescue

Figure 6. RAS Pathway Activation Is Associated with PD-L1 Upregulation in Human Cancers

(A) Heat-maps showing fold change in expression of T cell function related genes between high and low RAS pathway activity cohorts of lung adenocarcinoma (LUAD) and colon adenocarcinoma (COAD) TCGA samples. *KRAS* mutation status (codons 12, 13, and 61) is indicated for each sample. Genes are ranked in order of significance. Wald test, DESeq2.

(B) Box-and-whisker plots comparing PD-L1 mRNA expression in RAS high versus low pathway activity cohorts in LUAD and COAD using two independent RAS gene expression signatures. Wald test, DESeq2.

See also Figure S6.

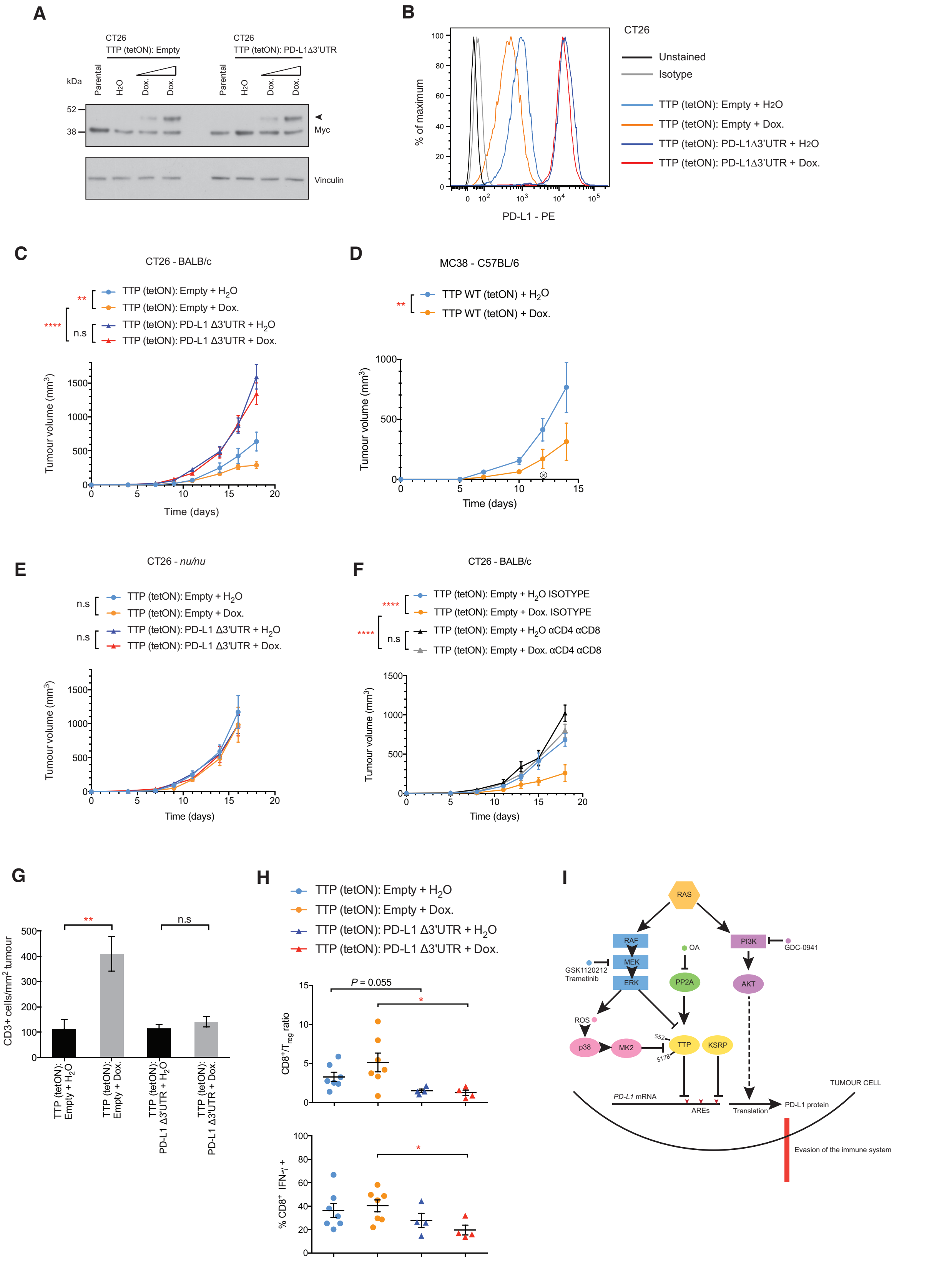

(legend on next page)

much of the growth inhibition mediated by doxycycline treatment in BALB/c mice, suggesting that suppression of tumor cell PD-L1 expression is an essential component of the anti-tumor effects mediated by TTP transgene induction (Figure 7C). As expected, CT26 cells expressing a *Cd274* cDNA with the full-length, wild-type 3′ UTR (PD-L1 WT 3′ UTR) had considerably lower expression of PD-L1 protein than the PD-L1 Δ3′ UTR cells, but still responded to TTP induction in terms of reductions in PD-L1 expression (Figure S7F) and control of tumor growth in immune-competent mice (Figure S7G).

Consistent with a heightened anti-tumor immune response, tumors derived from mice treated with doxycycline had a greater degree of $CD3^+$ lymphocyte infiltration than tumors from mice treated with vehicle, and this corresponding infiltration was abrogated in tumors derived from cells overexpressing PD-L1 Δ3′ UTR (Figures 7G and S7H). Moreover, we found higher $CD8^+$/Treg cell ratios in tumors expressing the TTP transgene and higher levels of IFN-γ production by $CD8^+$ tumor-infiltrating lymphocytes (TILs) derived from TTP-expressing tumors, versus PD-L1 Δ3′ UTR tumors expressing TTP (Figure 7H); however, we did not find significant differences in $CD4^+$ TIL populations (data not shown).

Collectively, these data highlight the functional importance of the regulation of PD-L1 expression by TTP in tumor progression and demonstrate that this novel regulatory pathway may be exploited for the treatment of *Ras* mutant cancers. These findings support a model whereby tumor-specific suppression of TTP can foster PD-L1 upregulation, and ultimately, tumor immunoresistance (Figures 7I and S7I).

DISCUSSION

In this report, we demonstrate that oncogenic RAS signaling can increase tumor cell-intrinsic PD-L1 expression, implying that mutant RAS oncogenes can directly contribute to the evasion of immune destruction in cancer. We revealed that RAS-MEK signaling controlled expression of PD-L1, at least in part, by modulating the stability of the transcript. We showed that the mouse and human PD-L1 mRNAs were labile transcripts containing functional AU-rich elements (AREs) in the 3′ UTR that permitted regulation of PD-L1 expression by RAS. Our data provide a potential explanation for the genomic structural variations in the *CD274* 3′ UTR observed in human cancer (Kataoka et al., 2016). The simultaneous loss of regulation by miRNAs and AREs is likely to contribute to the high overexpression observed in tumors with complete loss of the 3′ UTR. In addition, we provide a molecular basis for the tendency of *KRAS* mutant NSCLCs to be positive for PD-L1 expression (D'Incecco et al., 2015; Dong et al., 2017; Li et al., 2017; Yang et al., 2017), implying that PD-1-PD-L1 blockade may prove more successful in *RAS* mutant patients that also harbor a sufficient number of tumor antigens.

We identify TTP as a principle AU-rich element binding protein responsible for negatively regulating PD-L1 expression, consistent with a previous report identifying PD-L1 mRNA as one of a number of TTP targets in an RNA immunoprecipitation, microarray-based screen in mouse macrophages (Stoecklin et al., 2008). Mechanistically, MEK inhibition reduced PD-L1 mRNA stability, coinciding with an increase in TTP expression and reduction in phosphorylation of TTP at ERK and RSK/AKT consensus motifs. Conversely, activation of RAS and the associated ROS accumulation led to enhanced TTP phosphorylation, notably by MK2 at key inhibitory sites.

TTP transgene expression restrained tumor growth in CT26 and MC38 tumor transplantation models. This anti-tumor effect is predominantly non-cell autonomous, dependent on the adaptive immune system and suppression of tumor cell PD-L1 expression. We noted only minor reductions in tumor growth rates following TTP transgene induction in cells overexpressing PD-L1 Δ3′ UTR. TTP has been reported to have cell-autonomous tumor-suppressive roles (Rounbehler et al., 2012) and non-cell-autonomous anti-tumor effects through targeting VEGF and COX-2 mRNAs (Cha et al., 2011; Essafi-Benkhadir et al., 2007), which may contribute to some of these ostensibly PD-L1-independent effects, the magnitude of which are likely to be determined by the level of TTP overexpression in each system.

Our data extend the molecular understanding of the regulation of PD-L1 expression in cancer and highlight druggable targets to enhance anti-tumor immunity in tumors that are wild-type for the *CD274* 3′ UTR. We provide evidence that pharmacological targeting of RAS, or RAS effector proteins, may elicit non-cell-autonomous anti-tumor effects in *RAS* mutant tumors. Recently, MEK inhibitors and PD-1 pathway blockade were shown to combine strongly in a mouse model of *Ras* mutant colon

Figure 7. Restoration of Tumor Cell TTP Expression Enhances Anti-tumor Immunity

(A) Western blotting analysis of CT26 Myc-TTP tet-ON cells expressing either empty vector or mouse *Cd274* cDNA lacking the 3′ UTR (PD-L1 Δ3′ UTR), 24 hr after treatment (Dox., 0.1 μg/mL or 1 μg/mL). Arrow indicates Myc-TTP. Data are representative of two independent experiments.

(B) Representative flow cytometry histograms of PD-L1 surface protein expression in CT26 stable cells lines in (A), 72 hr after treatment (Dox., 1 μg/mL). Data are representative of three independent experiments.

(C) Tumor growth curves for CT26-derived cell lines subcutaneously transplanted into BALB/c mice (n = 8 per group).

(D) Tumor growth curves for MC38-derived cell lines subcutaneously transplanted into C57BL/6 mice (n = 6 per group). X denotes the loss of a doxycycline-treated mouse.

(E) Tumor growth curves for CT26-derived cell lines subcutaneously transplanted into *nu/nu* mice (n = 6 per group).

(F) Tumor growth curves for CT26-derived cell lines subcutaneously transplanted into BALB/c mice (n = 4–5 per group).

For (C)–(F), data represent the mean ± SEM from individual experiments. $^{**}p < 0.01$, $^{****}p < 0.0001$, n.s., not significant; two-way ANOVA.

(G) Histological analysis of subcutaneous tumors at the end-point from the experiment described in (C), with quantification of $CD3^+$ cells in 5 fields of view per mouse with 5–6 mice per group. Mean ± SEM. $^{**}p < 0.01$; unpaired, two-tailed Student's t test.

(H) Quantification of $CD8^+$/Treg cell ratios and $CD8^+$ IFN-γ^+ cells from flow cytometry analysis of tumors after 18–20 days of growth. Each data point represents data from an individual mouse; mean ± SEM. $^{*}p < 0.05$; unpaired, two-tailed Student's t test. Data are pooled from two independent experiments.

(I) Proposed molecular model. Signaling nodes that influence anti-tumor immunity and are amenable to inhibition with drugs used in this study are highlighted. S52 and S178 represent MK2 target sites and numbering corresponds to mouse TTP. OA, okadaic acid.

See also Figure S7.

carcinoma (Ebert et al., 2016; Liu et al., 2015). We anticipate that our findings will inform the development of effective combination therapies with immune checkpoint blockade in cancer.

STAR★METHODS

Detailed methods are provided in the online version of this paper and include the following:

- KEY RESOURCES TABLE
- CONTACT FOR REAGENT AND RESOURCE SHARING
- EXPERIMENTAL MODEL AND SUBJECT DETAILS
  - Cell Lines
  - *In vivo* studies
- METHOD DETAILS
  - *In vivo* studies
  - Transfections
  - Cloning, plasmids and stable cell lines
  - Flow cytometry
  - Immunoprecipitation
  - Immunohistochemistry
  - CRISPR/Cas
  - Bioinformatics
  - Mass Spectrometry
  - Western blotting
  - Luciferase assays
  - Quantitative real-time PCR (qPCR)
  - RNA-immunoprecipitation
- QUANTIFICATION AND STATISTICAL ANALYSIS

SUPPLEMENTAL INFORMATION

Supplemental Information includes seven figures and two tables and can be found with this article online at https://doi.org/10.1016/j.immuni.2017.11.016.

AUTHOR CONTRIBUTIONS

M.A.C. and J.D. designed the study, interpreted the results, and wrote the manuscript. M.A.C., S.C.T., and S.R. performed the biochemical experiments, D.Z. and C.M. assisted with *in vivo* studies, M.M.-A. provided reagents and conceptual advice, B.S.-D. and E.N. performed histopathological studies, S.C.T. and P.E. performed bioinformatics analyses, K.B. and A.P.S. performed mass spectrometry analyses, and W.S.L. and P.J.B. provided TTP KO and WT MEFs. All authors contributed to manuscript revision and review.

ACKNOWLEDGMENTS

We thank Charles Swanton and Caetano Reis e Sousa for helpful discussion, David Hancock and Febe Van Maldegem for critical reading of the manuscript, and science technology platforms at the Francis Crick Institute including Biological Resources, FACS, Equipment Park, Experimental Histopathology, and Cell Services. We thank Lynn McGregor and Kevan Shokat for providing ARS853. This work was supported by the Francis Crick Institute, which receives its core funding from Cancer Research UK (FC001070), the UK Medical Research Council (FC001070), and the Wellcome Trust (FC001070), and by funding to J.D. from the European Research Council Advanced Grant RASTARGET and a Wellcome Trust Senior Investigator Award (103799/Z/14/Z). This work was also supported in part by the Intramural Research Program of the National Institute of Environmental Health Sciences, NIH (W.S.L. and P.J.B.). S.C.T. was supported by a fellowship from the European Commission Marie Skłodowska-Curie Actions programme, iGEMMdev.

Received: October 14, 2016
Revised: June 6, 2017
Accepted: November 20, 2017
Published: December 12, 2017

REFERENCES

Akbay, E.A., Koyama, S., Carretero, J., Altabef, A., Tchaicha, J.H., Christensen, C.L., Mikse, O.R., Cherniack, A.D., Beauchamp, E.M., Pugh, T.J., et al. (2013). Activation of the PD-1 pathway contributes to immune escape in EGFR-driven lung tumors. Cancer Discov. *3*, 1355–1363.

Berthon, C., Driss, V., Liu, J., Kuranda, K., Leleu, X., Jouy, N., Hetuin, D., and Quesnel, B. (2010). In acute myeloid leukemia, B7-H1 (PD-L1) protection of blasts from cytotoxic T cells is induced by TLR ligands and interferon-gamma and can be reversed using MEK inhibitors. Cancer Immunol. Immunother. *59*, 1839–1849.

Borghaei, H., Paz-Ares, L., Horn, L., Spigel, D.R., Steins, M., Ready, N.E., Chow, L.Q., Vokes, E.E., Felip, E., Holgado, E., et al. (2015). Nivolumab versus docetaxel in advanced nonsquamous non-small-cell lung cancer. N. Engl. J. Med. *373*, 1627–1639.

Bourcier, C., Griseri, P., Grépin, R., Bertolotto, C., Mazure, N., and Pagès, G. (2011). Constitutive ERK activity induces downregulation of tristetraprolin, a major protein controlling interleukin8/CXCL8 mRNA stability in melanoma cells. Am. J. Physiol. Cell Physiol. *301*, C609–C618.

Brahmer, J., Reckamp, K.L., Baas, P., Crinò, L., Eberhardt, W.E., Poddubskaya, E., Antonia, S., Pluzanski, A., Vokes, E.E., Holgado, E., et al. (2015). Nivolumab versus docetaxel in advanced squamous-cell non-small-cell lung cancer. N. Engl. J. Med. *373*, 123–135.

Brook, M., Tchen, C.R., Santalucia, T., McIlrath, J., Arthur, J.S., Saklatvala, J., and Clark, A.R. (2006). Posttranslational regulation of tristetraprolin subcellular localization and protein stability by p38 mitogen-activated protein kinase and extracellular signal-regulated kinase pathways. Mol. Cell. Biol. *26*, 2408–2418.

Casey, S.C., Tong, L., Li, Y., Do, R., Walz, S., Fitzgerald, K.N., Gouw, A.M., Baylot, V., Gütgemann, I., Eilers, M., and Felsher, D.W. (2016). MYC regulates the antitumor immune response through CD47 and PD-L1. Science *352*, 227–231.

Cha, H.J., Lee, H.H., Chae, S.W., Cho, W.J., Kim, Y.M., Choi, H.J., Choi, D.H., Jung, S.W., Min, Y.J., Lee, B.J., et al. (2011). Tristetraprolin downregulates the expression of both VEGF and COX-2 in human colon cancer. Hepatogastroenterology *58*, 790–795.

Chrestensen, C.A., Schroeder, M.J., Shabanowitz, J., Hunt, D.F., Pelo, J.W., Worthington, M.T., and Sturgill, T.W. (2004). MAPKAP kinase 2 phosphorylates tristetraprolin on in vivo sites including Ser178, a site required for 14-3-3 binding. J. Biol. Chem. *279*, 10176–10184.

D'Incecco, A., Andreozzi, M., Ludovini, V., Rossi, E., Capodanno, A., Landi, L., Tibaldi, C., Minuti, G., Salvini, J., Coppi, E., et al. (2015). PD-1 and PD-L1 expression in molecularly selected non-small-cell lung cancer patients. Br. J. Cancer *112*, 95–102.

Deleault, K.M., Skinner, S.J., and Brooks, S.A. (2008). Tristetraprolin regulates TNF TNF-alpha mRNA stability via a proteasome dependent mechanism involving the combined action of the ERK and p38 pathways. Mol. Immunol. *45*, 13–24.

Díaz-Moreno, I., Hollingworth, D., Frenkiel, T.A., Kelly, G., Martin, S., Howell, S., García-Mayoral, M., Gherzi, R., Briata, P., and Ramos, A. (2009). Phosphorylation-mediated unfolding of a KH domain regulates KSRP localization via 14-3-3 binding. Nat. Struct. Mol. Biol. *16*, 238–246.

Dong, Z.Y., Zhong, W.Z., Zhang, X.C., Su, J., Xie, Z., Liu, S.Y., Tu, H.Y., Chen, H.J., Sun, Y.L., Zhou, Q., et al. (2017). Potential predictive value of TP53 and KRAS mutation status for response to PD-1 blockade immunotherapy in lung adenocarcinoma. Clin. Cancer Res. *23*, 3012–3024.

Du, L., Schageman, J.J., Subauste, M.C., Saber, B., Hammond, S.M., Prudkin, L., Wistuba, I.I., Ji, L., Roth, J.A., Minna, J.D., and Pertsemlidis, A. (2009). miR-93, miR-98, and miR-197 regulate expression of tumor suppressor gene FUS1. Mol. Cancer Res. *7*, 1234–1243.

Ebert, P.J.R., Cheung, J., Yang, Y., McNamara, E., Hong, R., Moskalenko, M., Gould, S.E., Maecker, H., Irving, B.A., Kim, J.M., et al. (2016). MAP kinase inhibition promotes T cell and anti-tumor activity in combination with PD-L1 checkpoint blockade. Immunity *44*, 609–621.

El-Jawhari, J.J., El-Sherbiny, Y.M., Scott, G.B., Morgan, R.S., Prestwich, R., Bowles, P.A., Blair, G.E., Tanaka, T., Rabbitts, T.H., Meade, J.L., and Cook, G.P. (2014). Blocking oncogenic RAS enhances tumour cell surface MHC class I expression but does not alter susceptibility to cytotoxic lymphocytes. Mol. Immunol. *58*, 160–168.

Essafi-Benkhadir, K., Onesto, C., Stebe, E., Moroni, C., and Pagès, G. (2007). Tristetraprolin inhibits Ras-dependent tumor vascularization by inducing vascular endothelial growth factor mRNA degradation. Mol. Biol. Cell *18*, 4648–4658.

Garner, A.P., Weston, C.R., Todd, D.E., Balmanno, K., and Cook, S.J. (2002). Delta MEKK3:ER* activation induces a p38 alpha/beta 2-dependent cell cycle arrest at the G2 checkpoint. Oncogene *21*, 8089–8104.

Garon, E.B., Rizvi, N.A., Hui, R., Leighl, N., Balmanoukian, A.S., Eder, J.P., Patnaik, A., Aggarwal, C., Gubens, M., Horn, L., et al.; KEYNOTE-001 Investigators (2015). Pembrolizumab for the treatment of non-small-cell lung cancer. N. Engl. J. Med. *372*, 2018–2028.

Giannou, A.D., Marazioti, A., Kanellakis, N.I., Giopanou, I., Lilis, I., Zazara, D.E., Ntaliarda, G., Kati, D., Armenis, V., Giotopoulou, G.A., et al. (2017). NRAS destines tumor cells to the lungs. EMBO Mol. Med. *9*, 672–686.

Gubin, M.M., Zhang, X., Schuster, H., Caron, E., Ward, J.P., Noguchi, T., Ivanova, Y., Hundal, J., Arthur, C.D., Krebber, W.J., et al. (2014). Checkpoint blockade cancer immunotherapy targets tumour-specific mutant antigens. Nature *515*, 577–581.

Hall, M.P., Huang, S., and Black, D.L. (2004). Differentiation-induced colocalization of the KH-type splicing regulatory protein with polypyrimidine tract binding protein and the c-src pre-mRNA. Mol. Biol. Cell *15*, 774–786.

Härdle, L., Bachmann, M., Bollmann, F., Pautz, A., Schmid, T., Eberhardt, W., Kleinert, H., Pfeilschifter, J., and Mühl, H. (2015). Tristetraprolin regulation of interleukin-22 production. Sci. Rep. *5*, 15112.

Herbst, R.S., Soria, J.C., Kowanetz, M., Fine, G.D., Hamid, O., Gordon, M.S., Sosman, J.A., McDermott, D.F., Powderly, J.D., Gettinger, S.N., et al. (2014). Predictive correlates of response to the anti-PD-L1 antibody MPDL3280A in cancer patients. Nature *515*, 563–567.

Jiang, X., Zhou, J., Giobbie-Hurder, A., Wargo, J., and Hodi, F.S. (2013). The activation of MAPK in melanoma cells resistant to BRAF inhibition promotes PD-L1 expression that is reversible by MEK and PI3K inhibition. Clin. Cancer Res. *19*, 598–609.

Kataoka, K., Shiraishi, Y., Takeda, Y., Sakata, S., Matsumoto, M., Nagano, S., Maeda, T., Nagata, Y., Kitanaka, A., Mizuno, S., et al. (2016). Aberrant PD-L1 expression through 3′-UTR disruption in multiple cancers. Nature *534*, 402–406.

Kemp, S.J., Thorley, A.J., Gorelik, J., Seckl, M.J., O'Hare, M.J., Arcaro, A., Korchev, Y., Goldstraw, P., and Tetley, T.D. (2008). Immortalization of human alveolar epithelial cells to investigate nanoparticle uptake. Am. J. Respir. Cell Mol. Biol. *39*, 591–597.

Lai, W.S., Parker, J.S., Grissom, S.F., Stumpo, D.J., and Blackshear, P.J. (2006). Novel mRNA targets for tristetraprolin (TTP) identified by global analysis of stabilized transcripts in TTP-deficient fibroblasts. Mol. Cell. Biol. *26*, 9196–9208.

Lau, J., Cheung, J., Navarro, A., Lianoglou, S., Haley, B., Totpal, K., Sanders, L., Koeppen, H., Caplazi, P., McBride, J., et al. (2017). Tumour and host cell PD-L1 is required to mediate suppression of anti-tumour immunity in mice. Nat. Commun. *8*, 14572.

Le, D.T., Uram, J.N., Wang, H., Bartlett, B.R., Kemberling, H., Eyring, A.D., Skora, A.D., Luber, B.S., Azad, N.S., Laheru, D., et al. (2015). PD-1 blockade in tumors with mismatch-repair deficiency. N. Engl. J. Med. *372*, 2509–2520.

Li, D., Zhu, X., Wang, H., and Li, N. (2017). Association between PD-L1 expression and driven gene status in NSCLC: A meta-analysis. Eur. J. Surg. Oncol. *43*, 1372–1379.

Linnemann, C., van Buuren, M.M., Bies, L., Verdegaal, E.M.E., Schotte, R., Calis, J.J.A., Behjati, S., Velds, A., Hilkmann, H., Atmioui, D.E., et al. (2015). High-throughput epitope discovery reveals frequent recognition of neo-antigens by CD4+ T cells in human melanoma. Nat. Med. *21*, 81–85.

Lito, P., Solomon, M., Li, L.S., Hansen, R., and Rosen, N. (2016). Allele-specific inhibitors inactivate mutant KRAS G12C by a trapping mechanism. Science *351*, 604–608.

Liu, L., Mayes, P.A., Eastman, S., Shi, H., Yadavilli, S., Zhang, T., Yang, J., Seestaller-Wehr, L., Zhang, S.Y., Hopson, C., et al. (2015). The BRAF and MEK inhibitors dabrafenib and trametinib: effects on immune function and in combination with immunomodulatory antibodies targeting PD-1, PD-L1, and CTLA-4. Clin. Cancer Res. *21*, 1639–1651.

Loboda, A., Nebozhyn, M., Klinghoffer, R., Frazier, J., Chastain, M., Arthur, W., Roberts, B., Zhang, T., Chenard, M., Haines, B., et al. (2010). A gene expression signature of RAS pathway dependence predicts response to PI3K and RAS pathway inhibitors and expands the population of RAS pathway activated tumors. BMC Med. Genomics *3*, 26.

Lykke-Andersen, J., and Wagner, E. (2005). Recruitment and activation of mRNA decay enzymes by two ARE-mediated decay activation domains in the proteins TTP and BRF-1. Genes Dev. *19*, 351–361.

Mahtani, K.R., Brook, M., Dean, J.L., Sully, G., Saklatvala, J., and Clark, A.R. (2001). Mitogen-activated protein kinase p38 controls the expression and posttranslational modification of tristetraprolin, a regulator of tumor necrosis factor alpha mRNA stability. Mol. Cell. Biol. *21*, 6461–6469.

Molina-Arcas, M., Hancock, D.C., Sheridan, C., Kumar, M.S., and Downward, J. (2013). Coordinate direct input of both KRAS and IGF1 receptor to activation of PI3 kinase in KRAS-mutant lung cancer. Cancer Discov. *3*, 548–563.

Nicke, B., Bastien, J., Khanna, S.J., Warne, P.H., Cowling, V., Cook, S.J., Peters, G., Delpuech, O., Schulze, A., Berns, K., et al. (2005). Involvement of MINK, a Ste20 family kinase, in Ras oncogene-induced growth arrest in human ovarian surface epithelial cells. Mol. Cell *20*, 673–685.

Pardoll, D.M. (2012). The blockade of immune checkpoints in cancer immunotherapy. Nat. Rev. Cancer *12*, 252–264.

Parsa, A.T., Waldron, J.S., Panner, A., Crane, C.A., Parney, I.F., Barry, J.J., Cachola, K.E., Murray, J.C., Tihan, T., Jensen, M.C., et al. (2007). Loss of tumor suppressor PTEN function increases B7-H1 expression and immunoresistance in glioma. Nat. Med. *13*, 84–88.

Patricelli, M.P., Janes, M.R., Li, L.S., Hansen, R., Peters, U., Kessler, L.V., Chen, Y., Kucharski, J.M., Feng, J., Ely, T., et al. (2016). Selective inhibition of oncogenic KRAS output with small molecules targeting the inactive state. Cancer Discov. *6*, 316–329.

Rajagopalan, L.E., Burkholder, J.K., Turner, J., Culp, J., Yang, N.S., and Malter, J.S. (1995). Granulocyte-macrophage colony-stimulating factor mRNA stabilization enhances transgenic expression in normal cells and tissues. Blood *86*, 2551–2558.

Rizvi, N.A., Hellmann, M.D., Snyder, A., Kvistborg, P., Makarov, V., Havel, J.J., Lee, W., Yuan, J., Wong, P., Ho, T.S., et al. (2015). Cancer immunology. Mutational landscape determines sensitivity to PD-1 blockade in non-small cell lung cancer. Science *348*, 124–128.

Rounbehler, R.J., Fallahi, M., Yang, C., Steeves, M.A., Li, W., Doherty, J.R., Schaub, F.X., Sanduja, S., Dixon, D.A., Blackshear, P.J., and Cleveland, J.L. (2012). Tristetraprolin impairs myc-induced lymphoma and abolishes the malignant state. Cell *150*, 563–574.

Selamat, S.A., Chung, B.S., Girard, L., Zhang, W., Zhang, Y., Campan, M., Siegmund, K.D., Koss, M.N., Hagen, J.A., Lam, W.L., et al. (2012). Genome-scale analysis of DNA methylation in lung adenocarcinoma and integration with mRNA expression. Genome Res. *22*, 1197–1211.

Skrzypczak, M., Goryca, K., Rubel, T., Paziewska, A., Mikula, M., Jarosz, D., Pachlewski, J., Oledzki, J., and Ostrowski, J. (2010). Modeling oncogenic signaling in colon tumors by multidirectional analyses of microarray data directed for maximization of analytical reliability. PLoS ONE *5*, 5.

Stoecklin, G., Stubbs, T., Kedersha, N., Wax, S., Rigby, W.F., Blackwell, T.K., and Anderson, P. (2004). MK2-induced tristetraprolin:14-3-3 complexes

prevent stress granule association and ARE-mRNA decay. EMBO J. *23*, 1313–1324.

Stoecklin, G., Tenenbaum, S.A., Mayo, T., Chittur, S.V., George, A.D., Baroni, T.E., Blackshear, P.J., and Anderson, P. (2008). Genome-wide analysis identifies interleukin-10 mRNA as target of tristetraprolin. J. Biol. Chem. *283*, 11689–11699.

Sumimoto, H., Takano, A., Teramoto, K., and Daigo, Y. (2016). RAS-mitogen-activated protein kinase signal is required for enhanced PD-L1 expression in human lung cancers. PLoS ONE *11*, e0166626.

Sun, L., Stoecklin, G., Van Way, S., Hinkovska-Galcheva, V., Guo, R.F., Anderson, P., and Shanley, T.P. (2007). Tristetraprolin (TTP)-14-3-3 complex formation protects TTP from dephosphorylation by protein phosphatase 2a and stabilizes tumor necrosis factor-alpha mRNA. J. Biol. Chem. *282*, 3766–3777.

Sweet-Cordero, A., Mukherjee, S., Subramanian, A., You, H., Roix, J.J., Ladd-Acosta, C., Mesirov, J., Golub, T.R., and Jacks, T. (2005). An oncogenic KRAS2 expression signature identified by cross-species gene-expression analysis. Nat. Genet. *37*, 48–55.

Taylor, G.A., Thompson, M.J., Lai, W.S., and Blackshear, P.J. (1995). Phosphorylation of tristetraprolin, a potential zinc finger transcription factor, by mitogen stimulation in intact cells and by mitogen-activated protein kinase in vitro. J. Biol. Chem. *270*, 13341–13347.

Taylor, G.A., Carballo, E., Lee, D.M., Lai, W.S., Thompson, M.J., Patel, D.D., Schenkman, D.I., Gilkeson, G.S., Broxmeyer, H.E., Haynes, B.F., and Blackshear, P.J. (1996). A pathogenetic role for TNF alpha in the syndrome of cachexia, arthritis, and autoimmunity resulting from tristetraprolin (TTP) deficiency. Immunity *4*, 445–454.

Topalian, S.L., Hodi, F.S., Brahmer, J.R., Gettinger, S.N., Smith, D.C., McDermott, D.F., Powderly, J.D., Carvajal, R.D., Sosman, J.A., Atkins, M.B., et al. (2012). Safety, activity, and immune correlates of anti-PD-1 antibody in cancer. N. Engl. J. Med. *366*, 2443–2454.

Yang, N.S., Wang, J.H., and Turner, J. (2004). Molecular strategies for improving cytokine transgene expression in normal and malignant tissues. Gene Ther. *11*, 100–108.

Yang, H., Chen, H., Luo, S., Li, L., Zhou, S., Shen, R., Lin, H., and Xie, X. (2017). The correlation between programmed death-ligand 1 expression and driver gene mutations in NSCLC. Oncotarget *8*, 23517–23528.

Zubiaga, A.M., Belasco, J.G., and Greenberg, M.E. (1995). The nonamer UUAUUUAUU is the key AU-rich sequence motif that mediates mRNA degradation. Mol. Cell. Biol. *15*, 2219–2230.

STAR★METHODS

KEY RESOURCES TABLE

REAGENT or RESOURCE	SOURCE	IDENTIFIER
Antibodies		
p-ERK	Cell Signaling Technology	Cat# 9101
p-AKT (S473)	Cell Signaling Technology	Cat# 9271
PD-L1 (anti-human)	eBioscience	Cat# 12-5983-42
Isotype control	eBioscience	Cat# 9012-4714-025
PD-L1 (anti-mouse)	eBioscience	Cat# 14-5982-82
Isotype control	eBioscience	Cat# 12-4321-41
CD31	eBioscience	Cat# 11-0311-81
TTP	Santa Cruz	Cat# sc-8458
TTP endogenous	Merck Millipore	Cat# ABE285
KSRP	Cambridge Bioscience	Cat# A302-021A
KSRP	Cell Signaling Technology	Cat# 5398S
Myc (9E10)	Francis Crick Institute Cell Services	N/A
ERK	Cell Signaling Technology	Cat# 9107
AKT	Cell Signaling Technology	Cat# 2920
p-PXSP	Cell Signaling Technology	Cat# 2325
p-RXXS/T	Cell Signaling Technology	Cat# 9611
p-p38 (T180/Y182)	Cell Signaling Technology	Cat# 9211
CD3	Abcam	Cat# ab134096
IFN-γ XMG1.2	eBioscience	Cat# 12-7311-41
Foxp3 FJK16s	eBioscience	Cat# 72-5775-40
CD45 30-F11	BioLegend	Cat# 103129
CD4 GK1.5	Francis Crick Institute Cell Services	N/A
CD4 GK1.5	eBioscience	Cat# 25-0041-81
CD4 RM4-5	eBioscience	Cat# 11-0042-81
CD8 53-6.7	BD Biosciences	Cat# 563786
CD8 2.43	Francis Crick Institute Cell Services	N/A
CD3 17A2	BioLegend	Cat# 100204
CD45	eBioscience	Cat# 11-0451-82
IFN-γ NIB42	eBioscience	Cat# 14-7318-81
Bacterial and Virus Strains		
Adenovirus for expression of Cre recombinase	Gene Transfer Vector Core. University of Iowa.	N/A
XL10-Gold Ultracompetent Cells	Agilent Technologies	Cat# 200314
Chemicals, Peptides, and Recombinant Proteins		
Lipofectamine 2000	ThermoFisher	Cat# 11668027
DharmaFECT 1	Dharmacon	Cat# T-2001-01
Trametinib	Selleckchem	Cat# S2673
ARS853	A gift from the Shokat Laboratory	N/A
GDC-0941	Selleckchem	Cat# S1065
4-OHT	Sigma-Aldrich	Cat# H7904
PMA	Sigma-Aldrich	Cat# P1585
Actinomycin D	Sigma-Aldrich	Cat# A1410
N-Acetyl-L-cysteine	Sigma-Aldrich	Cat# A9165
Glutathione reduced ethyl ester	Sigma-Aldrich	Cat# G1404
PF-3644022 hydrate	Sigma-Aldrich	Cat# PZ0188

(*Continued on next page*)

Continued

REAGENT or RESOURCE	SOURCE	IDENTIFIER
MK2 inhibitor III	Merck Millipore	Cat# 475864
Doxycycline hyclate	Sigma-Aldrich	Cat# D9891
Ionomycin	Sigma-Aldrich	Cat# I0634
GolgiPlug	BD Bioscience	Cat# BDB555029
Human Interferon gamma	Biolegend	Cat# 570206
Ruxolitinib	Selleckchem	Cat# S1378
DAPI	ThermoFisher	Cat# D1306
Fixable viability dye eFluor 780	eBioscience	Cat# 65-0865-14
H_2DCFDA	ThermoFisher	Cat# C6827
Critical Commercial Assays		
Dual-Luciferase Reporter Assay	Promega	Cat# E1910
Magna-RIP Kit	Merck Millipore	Cat# 17-700
Anti-Mouse/Rat Foxp3 Staining Set PE	eBioscience	Cat# 72-5775-40
QuikChange II XL Site-Directed Mutagenesis Kit	Agilent Technologies	Cat# 200521
RNeasy Mini Kit	QIAGEN	Cat# 74104
Dynabeads Protein G for Immunoprecipitation	ThermoFisher	Cat# 10003D
SYBR Green Fast Master Mix	ThermoFisher	Cat# A25742
Experimental Models: Cell Lines		
H358	ATCC	N/A
A427	Francis Crick Institute Cell Services	N/A
H1792	ATCC	N/A
KPB6	Sergio Quezada Laboratory	N/A
Type II pneumocytes	Olivier Pardo, Michael Seckl (Imperial College, London) and (Molina-Arcas et al., 2013)	N/A
SW837	Francis Crick Institute Cell Services	N/A
H23	Francis Crick Institute Cell Services	N/A
293FT	Francis Crick Institute Cell Services	N/A
TTP KO and TTP WT MEFs	Perry Blackshear Laboratory	N/A
MCF10A	Molina-Arcas et al., 2013	N/A
CT26	Francis Crick Institute Cell Services	N/A
A549	Francis Crick Institute Cell Services	N/A
MC38	George Kassiotis Laboratory	N/A
Experimental Models: Organisms/Strains		
C57BL/6	The Francis Crick Institute Biological Resources Unit; Originally The Jackson Laboratory	Stock Number #000664
BALB/c	The Francis Crick Institute Biological Resources Unit; Originally The Jackson Laboratory	Stock Number #001026
Kras$^{LSL\text{-}G12D/+}$/*Trp53*$^{Flox/Flox}$ (B6.129-*Kras*^{tm4Tyj}/Nci and FVB.129P2-*Trp53*^{tm1Brn}/Nci)	The Francis Crick Institute Biological Resources Unit; Originally Mouse Models of Human Cancer Consortium	Strain Number# 01XJ6; 01XC2
Nu/nu (Foxn1^{nu})	The Francis Crick Institute Biological Resources Unit; Originally ICRF Stock	N/A
Oligonucleotides		
siGENOME x4 TTP	Dharmacon	Cat# MU-010789-02-0002
siGENOME x4 AUF1	Dharmacon	Cat# MU-004079-01-0002
siGENOME x4 KSRP	Dharmacon	Cat# MU-009490-01-0002
siGENOME x4 HuR	Dharmacon	Cat# MU-003773-04-0002
siGENOME x4 BRF1	Dharmacon	Cat# MU-011816-00-0002
siGENOME x4 BRF2	Dharmacon	Cat# MU-013605-02-0002
siScramble Control	Dharmacon	Cat# D-001810-10-05

(*Continued on next page*)

Continued		
REAGENT or RESOURCE	SOURCE	IDENTIFIER
Recombinant DNA		
peGFPC1-6XHis-FL-KSRP	A gift from Douglas Black	Addgene plasmid # 23001
PD-L1 + wild type 3′UTR mouse ORF clone NM_021893.3	Creative Biogene	N/A
pUNO-mcs	InvivoGen	Cat# puno1-mcs
pTRIPZ	Dharmacon	Cat# RHS4740
pcDNA3.1	ThermoFisher	Cat# V79020
pGL3 Control	Promega	Cat# E1741
pGL3 Basic	Promega	Cat# E1751
pRL-TK Control	Promega	Cat# E2241
Software and Algorithms		
FlowJo	Tree Star	N/A
Prism 7	GraphPad	N/A
Skyline v.3.5.0.9319	McCoss Lab Software	N/A

CONTACT FOR REAGENT AND RESOURCE SHARING

Further information and requests for resources and reagents should be directed to and will be fulfilled by the Lead Contact, Julian Downward (julian.downward@crick.ac.uk).

EXPERIMENTAL MODEL AND SUBJECT DETAILS

Cell Lines

Specific culture conditions and origin of all the cell lines used in this study are listed in the Key Resources Table and Table S2. Cell lines were authenticated by STR profiling by Cell Services at the Francis Crick Institute. Cells and antibodies used for *in vivo* studies were independently tested for common rodent pathogens and were certified as pathogen-free.

In vivo studies

All studies were performed under a UK Home Office approved project license and in accordance with institutional welfare guidelines. For tumor studies, we used 8-10 week old BALB/c or *nu/nu* (*Foxn1*^{nu}) mice (for CT26 cells) or 16-17 week old C57BL/6 mice (for MC38 cells). Sex-matched mice were randomly assigned into experimental groups before tumor cell injection. Group sizes are indicated in the figure legends.

For autochthonous tumor formation, *Kras*$^{LSL\text{-}G12D/+}$; *Trp53*$^{F/F}$ mice were sourced from the Mouse Models of Human Cancer Consortium (B6.129-*Kras*^{tm4Tyj}/Nci and FVB.129P2-*Trp53*^{tm1Brn}/Nci) and were backcrossed to C57BL/6 for 6 generations. Lung tumors were initiated using intratracheal intubation of 1x10^6 pfu adenovirus expressing Cre-recombinase (Gene Transfer Vector Core) in mice between 6-12 weeks of age. Lung tumor or normal lung tissue was analyzed 12 weeks after infection.

METHOD DETAILS

In vivo studies

Mice received 1x10^5 cells in PBS by subcutaneous injection into the left flank. Mice were treated with water or doxycycline by oral gavage (50 mg/kg) on day three after cell injection and then daily, with a two-day break every five days of treatment. For CD4$^+$ and CD8$^+$ cell depletion experiments, mice received 300 μg of GK1.5 and 300 μg of 2.43 monoclonal antibodies or rat IgG2b isotype control by i.p. administration three days before tumor cell engraftment and then twice weekly for the duration of the experiment. Depletion of CD8$^+$ and CD4$^+$ T cells was verified by flow cytometry using detection antibodies recognizing distinct epitopes from the depletion antibodies. Tumors were measured using callipers and volume was estimated using the formula: width^2 x length x 0.5, where length is the longest dimension and width is the corresponding perpendicular dimension.

Transfections

For RNA interference, cells were reverse-transfected with a final concentration of 50 nM siGENOME siRNA pools or ON-TARGETplus Non-targeting pool (“SiScrambled” control) or 25 nM for single deconvoluted siRNAs, and DharmaFECT 1 transfection reagent (Dharmacon; GE Healthcare) in 96 well plates. For transfection with TTP or KSRP constructs, cells were seeded in a 12 well plate and the following day transfected using Lipofectamine 2000 (Life Technologies).

Cloning, plasmids and stable cell lines

peGFPC1-6XHis-FL-KSRP was a gift from Douglas Black (Addgene plasmid # 23001)(Hall et al., 2004) and the S193A mutant was generated by site-directed mutagenesis (QuikChange II; Agilent Technologies). Full length human TTP was cloned from H358 genomic DNA into pcDNA3-MycX2 generating two N-terminal Myc tags. The S218 S228A TTP human and S52A S178A mouse double mutant constructs were generated by site-directed mutagenesis (QuikChange II; Agilent Technologies).

For the human PD-L1 (*CD274* gene) we refer to GRCh38:CM000671.2. For human PD-L1 mRNA we refer to NM_014143. For mouse PD-L1 (*Cd274* gene) we refer to GRCm38:CM001012.2. For mouse PD-L1 mRNA we refer to NM_021893. For the 3′UTR luciferase reporter constructs, the full length human *CD274* 3′UTR was cloned from H358 genomic DNA into the TOPO-TA vector (Life Technologies). The six most 3′ ATTTA pentamers (including the three most highly conserved, as shown in Figure 2D) were mutated to ATGTA (QuikChange Multi-site; Agilent). Wild-type and mutant fragments were subcloned into the Xba1, BamH1 site of pGL3-Control (Promega) to generate the reporter constructs.

CT26 cells were transfected with linearized pUNO empty and pUNO-mouse *Cd274* Δ3′UTR plasmids (InvivoGen) before selection with blasticidin, and for *Cd274* Δ3′UTR cells (‘PD-L1 Δ3′UTR’), subsequent FACS sorting of PD-L1 high, blasticidin-resistant cells. PD-L1 with the wild-type 3′UTR was subcloned from pcDNA3.1 mouse ORF clone NM_021893.3 (Creative Biogene) into the pUNO vector, linearized and transfected into CT26 cells to generate a stable cell line following selection with blasticidin and sorting for PD-L1 high cells.

For the lentiviral pTRIPZ constructs, full-length mouse TTP (*Zfp36*) was cloned from KPB6 genomic DNA into pcDNA3-MycX2 generating two N-terminal Myc tags. MycX2-TTP was subsequently subcloned into the Age1-Mlu1 site of pTRIPZ-empty (GE Healthcare), resulting in the final TTP (tet-ON) construct, without the TurboRFP or shRNAmir-related elements of the parental pTRIPZ plasmid. Lentiviral particles were produced by co-transfection of 293FT cells with pTRIPZ-TTP, psPAX2 and pMD2.G plasmids and the infected CT26 or MC38 target cells were selected with puromycin to establish stable cell lines.

For *CD274* promoter reporter constructs, pGL3-Basic (Promega) served as a negative control and pGL3-Control (Promega) served as a positive control for firefly luciferase expression. The indicated fragments of the human *CD274* promoter region were cloned from H358 genomic DNA into the MluI – XhoI site of pGL3-Basic. In addition, the putative enhancer site in intron 1 of the human *CD274* gene was cloned into the BamH1 – SalI site (downstream of the firefly luciferase ORF) of the 1 kb *CD274* promoter pGL3-Basic reporter construct, as shown in Figure S2A. Sequencing of these constructs and comparison to the GRCh38 assembly revealed two documented SNPs in the putative enhancer region fragment: rs4742097 and rs2282055.

Flow cytometry

Lung tissue was harvested in ice-cold PBS before mincing and then enzymatic digestion in Liberase TM and Liberase TH (both 75 μg/ml final; Roche) or collagenase I (1 mg/ml) with DNaseI (25 μg/ml final; Sigma) in HBSS (GIBCO) for 45 min at 37°C. After washing in DMEM + 10% FCS, cells were filtered through 70 μm filters (BD Bioscience) and then washed in FACS buffer (PBS supplemented with 2 mM EDTA and 0.5% BSA v/v final). Samples were then treated with Red Blood Cell Lysis Buffer (QIAGEN), washed in FACS buffer, filtered again and resuspended with FcR blocking reagent (BD Bioscience) before antibody staining of cell surface antigens in FACS buffer. For unfixed cells, samples were washed twice in FACS buffer and resuspended in DAPI (1 μg/ml final; eBioscience) immediately before analysis on LSRII or LSRFortessa (BD Biosciences) cell analysers. Intracellular staining for Foxp3 and IFN-γ was performed on fixed cells using the Foxp3 Staining Set (eBiosicence) according to manufacturer’s instructions. $CD4^+$ Tregs were defined by Foxp3 positivity. For IFN-γ staining, cells were stimulated for 4 h *ex-vivo* with PMA (20 ng/ml) and ionomycin (1 μg/ml) in the presence of GolgiPlug (BD Biosciences). Dead cells were excluded using the fixable viability dye eFluor 780 (eBioscience).

For FACS analysis of cell lines, cells were harvested with trypsin, washed in media and filtered before antibody staining in FACS buffer. Samples were washed twice in FACS buffer and resuspended in DAPI (1 μg/ml final; eBioscience) immediately before analysis. For the detection of intracellular ROS, adherent cells were washed in PBS before staining in 5 μM H_2DCFDA for 20 min in PBS at 37°C. Cells were then harvested by trypsinisation and prepared for flow cytometry as described above.

Immunoprecipitation

For each immunoprecipitation reaction, 25 μl slurry of Dynabeads (Life Technologies) were coupled with 3 μg of anti-Myc antibody (9E10; in-house) or normal mouse IgG. For Figure 5H, cross-linking was performed using DSS following manufacturer’s instructions (ThermoFisher). Beads were washed in Lysis Buffer (20 mM Tris-HCl, pH 7.4, 137.5 mM NaCl, 10% glycerol, 1% Triton X-100) and incubated overnight with rotation at 4°C with cleared cell lysates prepared in Lysis Buffer supplemented with protease and phosphatase inhibitor cocktails (Calbiochem). Beads were washed three times with IP Wash Buffer (modified Lysis Buffer: 0.1% Triton X-100, final), before elution with LDS Sample Buffer (Life Technologies).

Immunohistochemistry

Tissue was prepared for histology by incubation in 10% NBF for 24 h followed by 70% ethanol for a further 24 h before embedding in paraffin. For CD3 staining, sections were boiled in sodium citrate buffer (pH6) for 15 min and incubated for 1 h in anti-CD3 antibody

(ab134096; Abcam), followed by biotinylated secondary antibody and HRP/DAB detection. Tumors from *nu/nu* mice served as a negative control for CD3 staining. Hematoxylin and eosin staining was performed using standard methods.

CRISPR/Cas

The CRISPR/Cas genome editing was performed on CT26 cells using a U6gRNA-Cas9-2A-GFP construct targeting mouse *Zfp36* with a gRNA sequence GTCATGGCTCATCGACTGGAGG (Sigma, MM0000323992). Following plasmid transfection, single GFP-positive cells were selected by FACS for expansion in culture. Transfection with Cas9-2A-GFP alone served as a negative control. KO of functional TTP was confirmed by western blotting and complete *Zfp36* allele disruption was confirmed by TOPO-TA cloning followed by sequencing.

Bioinformatics

Using two published RAS activation gene expression signatures (Loboda et al., 2010; Sweet-Cordero et al., 2005), we identified high and low RAS pathway activity LUAD TCGA RNASeq samples. We determined high and low RAS pathway activity using GSEA (GeneSetTest, Bioconductor) against genes ranked by their log2 normalized counts scaled across all tumor samples. Only the upregulated genes from the signatures were used in the GSEA. Samples with a significant GSEA association (FDR < 0.05) of a RAS signature to the upper portion of the rank were assigned as having high RAS activity. Those with a significant association to the lower portion of the rank were assigned as having low RAS activity. Once assigned, we identified RAS-dependent gene expression changes between the high and low RAS activity groups by standard RNASeq analysis methods (DESeq2, FDR < 0.05). A short-list of “T cell Function” related genes was generated from gene ontology annotation based on the nanoString Technologies nCounter Human PanCancer Immune Profiling Panel.

Mass Spectrometry

Gel bands were excised and subjected to digestion with trypsin. Tryptic peptides were analyzed by LC-MS using Ultimate 3000 uHPLC system connected to a Q-Exactive mass spectrometer (Thermo Fisher Scientific) and acquired in data-dependent mode (DDA) for identification and in targeted SIM/PRM mode for quantification. A SIM isolation list was setup for the following peptides: STSLVEGR (m/z 424.7272, 2+, non phos), STSLVEGR (m/z 464.7104, 2+, phos S52), QSISFSGLPSGR (m/z 618.3276, 2+, non phos) and QSISFSGLPSGR (m/z 658.3057, 2+, phos S178). For SIM/PRM scans, MS1 peaks were acquired at resolution of 70,000 (at m/z 200) and scan time (1x256 ms); MS2 fragment ion resolution was 17,500 (at m/z 200) scan time (64x4 ms); and SIM/PRM cycle time was (1280 ms). For identification and generation of spectral libraries, the resulting DDA data was searched against a mouse Uniprot database containing common contaminants (UniProt_KB2012_08_taxonomy_mouse_10090_canonical_with_contaminants.fasta) as well as a custom database containing the Myc-tagged mouse Zfp36 sequence using the Andromeda search engine and MaxQuant (version 1.3.0.5). For MaxQuant, a false discovery rate of 0.1% was used to generate protein, peptide and site identification tables. The targeted mass spectrometry raw data was uploaded into Skyline (version 3.5.0.9319) for identification, quantification and further statistical analyses.

Western blotting

Western blotting was performed using standard methods. Primary antibodies used are listed in the Key Resources Table. Secondary antibodies were conjugated to horseradish peroxidase (GE Healthcare).

Luciferase assays

H358, ER-HRAS^{G12V} MCF10A and KP (tetON) cells were plated in 96 well plates and the following day co-transfected with pRL-TK control and pGL3-3′UTR PD-L1 luciferase constructs using Lipofectamine 2000 (Life Technologies). 24 h after transfection, PMA (200 nM; Sigma), doxycycline (1 μg/ml; Sigma) or MEK inhibitor GSK1120212 (25 nM; Selleckchem) was added, and 6-7 h later the Dual-Luciferase Reporter Assay (Promega) was performed. For ER-HRAS^{G12V} MCF10A, 24 h after transfection cells were serum-starved overnight, and then treated with 4-OHT (100 nM) for 24 h before the Dual-Luciferase Reporter Assay (Promega) was performed.

Quantitative real-time PCR (qPCR)

RNA was extracted using RNeasy Mini Kit (QIAGEN), cDNA was generated using SuperScript VILO or SuperScript II Reverse Transcriptase (Life Technologies) and qPCR reactions were carried out using QuantiTect Primer Assays (QIAGEN) and SYBR Green reagents (Life Technologies). Gene expression changes relative to the stated housekeeping gene were calculated using the $\Delta\Delta$CT method.

RNA-immunoprecipitation

RNA-immunoprecipitation (RNA-IP) reactions were carried out using Magna-RIP RNA-IP Kit (Millipore) with IgG control, anti-TTP or anti-KSRP antibodies according to the manufacturer’s instructions, except for the exclusion of EDTA from lysis and wash buffers, as

TTP is a zinc-finger protein. Total RNA was isolated and qPCR was carried out using methods specified in the above section, except using the % input method to calculate RNA enrichment.

QUANTIFICATION AND STATISTICAL ANALYSIS

Statistical tests, *p*-values, replicates and the definition of center and dispersion are indicated in the figures and figure legends. Unless otherwise stated in the figure legend, we used an unpaired, two-tailed Student's t test, where statistical significance was defined by $p < 0.05$. Statistical analyses were carried out in GraphPad Prism 7.

Immunity, Volume *47*

Supplemental Information

Oncogenic RAS Signaling Promotes Tumor Immunoresistance by Stabilizing PD-L1 mRNA

Matthew A. Coelho, Sophie de Carné Trécesson, Sareena Rana, Davide Zecchin, Christopher Moore, Miriam Molina-Arcas, Philip East, Bradley Spencer-Dene, Emma Nye, Karin Barnouin, Ambrosius P. Snijders, Wi S. Lai, Perry J. Blackshear, and Julian Downward

Supplemental Figure Legends

Figure S1, related to Figure 1. Cell-intrinsic Upregulation of PD-L1 through Oncogenic RAS Signalling

(**A**) qPCR analysis of PD-L1 mRNA expression at 6 h and 24 h after addition of 4-OHT or EtOH vehicle in ER-HRAS^{G12V} MCF10A cells. Mean ± SEM of two independent experiments. ****P<0.0001; unpaired, two-tailed Student's t-tests.

(**B**) Flow cytometry analysis of PD-L1 surface protein expression at 24 h and four days after addition of 4-OHT or EtOH vehicle in ER-HRAS^{G12V} MCF10A cells and four days in ER-HRAS^{G12V} HKE-3 cells.

(**C**) Flow cytometry analysis of PD-L1 surface protein four days after addition of 4-OHT or EtOH vehicle to parental MCF10A and HKE-3 cells.

(**D**) Western blotting analysis of ER-KRAS^{G12V} type II pneumocytes treated with 4-OHT, MEK inhibitor or PI3K inhibitor in starvation medium for 24 h.

(**E**) qPCR analysis of PD-L1 expression in H23 and H1792 cells 24 h after addition of MEK inhibitor or PI3K inhibitor or the combination. Mean ± SEM of three (H23) or two (H1792) independent experiments. **P<0.005, *P<0.05; unpaired, two-tailed Student's t-tests.

(**F**) qPCR analysis of PD-L1 expression in H358 and H23 cells 24 h after addition of the ERK1/2 inhibitor SCH772984 (500 nM). Mean ± SD of biological duplicates. **P<0.01; unpaired, two-tailed Student's t-tests.

(**G**) qPCR analysis of *PD-L1* expression in H1792 cells treated with PMA for 3 h following a 30 min pre-treatment with DMSO or MEK inhibitor. Data represent the mean ± SEM of two independent experiments. ****P<0.0001; unpaired, two-tailed Student's t-test.

(**H**) Surface expression of PD-L1 was measured by flow cytometry. MFI values are adjusted for the isotype control in each condition. Mean ± SEM of biological duplicates.

(**I**) qPCR analysis of transcripts encoding antigen processing and presentation machinery in ER-KRAS^{G12V} type II pneumocytes simulated with 4-OHT for 24

h in starvation medium. Data represent the mean ± SEM of four independent experiments. **P<0.01; paired, two-tailed Student's *t*-test.

MFI, Mean Fluorescence Intensity. 4-OHT, 100 nM. IFN-γ, 20 ng/ml. MEK inhibitor GSK1120212, 25 nM. PI3K inhibitor GDC-0941, 500 nM. PMA, 200 nM.

Figure S2, relating to Figure 2. RAS Signalling Increases PD-L1 mRNA Stability through AU-rich Elements in the 3'UTR

(**A**) Normalised luciferase signal from the indicated human *CD274* promoter region reporter constructs in H358 cells treated for 6.5 h with medium only, PMA (200 nM) or IFN-γ (20 ng/ml). Numbering corresponds to the GRCh38 assembly. Data are representative of two independent experiments. ** $P<0.005$, two-way ANOVA.

(**B**) Stability of murine PD-L1 mRNA measured by qPCR after the addition of actinomycin D (5 µg/ml) and DMSO or PI3K inhibitor GDC-0941 (500 nM). KPB6 cells were pre-treated with DMSO or PI3K inhibitor for 30 min before actinomycin D addition. Data represent the mean ± SEM and are normalised to the 0 h time point when actinomycin D was added, and are representative of two independent experiments.

(**C**) Stability of murine Tusc2 mRNA (left panel) and Ptgs2 mRNA (right panel) measured by qPCR after the addition of actinomycin D (5 µg/ml) and DMSO or MEK inhibitor GSK1120212 (25 nM). KPB6 cells were pre-treated with DMSO or MEK inhibitor for 30 min before actinomycin D addition. Data represent the mean ± SEM and are normalised to the 0 h time point when actinomycin D was added.

Figure S3, relating to Figure 3. AU-rich element Binding Proteins TTP and KSRP are Negative Regulators of PD-L1 Expression

(**A-C**) qPCR analysis of knockdown efficiency 48 h after siRNA transfections, relative to siScrambled control. Data represent the mean ± SD of triplicates.

(**D**) qPCR analysis of PD-L1 expression 24 h after transfection with the indicated constructs. Data represent the mean ± SEM of two independent experiments. *****P*<0.0001; ***P*<0.01; unpaired, two-tailed Student's *t*-test.

(**E**) qPCR analysis of PD-L1 expression in H23 cells 48 h after transfection with siRNAs targeting AU-rich binding proteins (AU-BPs), relative to siScrambled (siSc) control. Data represent the mean ± SEM of two independent experiments.

(**F**) qPCR analysis of knock-down efficiency in H23 cells 48 h after siRNA transfections, relative to siScrambled control. Data represent the mean ± SEM of two independent experiments.

(**G**) Western blotting analysis of TTP expression in H23, A427 and H358 cells 48 h after siRNA transfection with siRNA pools against TTP relative to siScrambled. Overexpression of Myc-TTP serves as a positive control for immunodetection.

(**H**) qPCR analysis of PD-L1 and TTP expression in H23 and H358 cells 48 h after siRNA transfection with siRNA pools or single siRNAs against TTP relative to siScrambled. Data represent the mean ± SEM of biological duplicates.

Figure S4, relating to Figure 4. RAS Regulates PD-L1 Expression through TTP

(**A**) qPCR analysis of PD-L1 and KSRP expression in H358 cells following siRNA mediated knock-down of KSRP (24 h) followed by MEK inhibition (24 h) with GSK1120212 (25 nM). Data represent the mean ± SEM of two independent experiments. $^{****}P<0.0001$; $^{***}P<0.001$; $^{**}P<0.005$; $^{*}P<0.05$; n.s; not significant.

(**B**) qPCR analysis of PD-L1 mRNA from RNA immunoprecipitates using IgG control or anti-TTP antibody, or anti-KSRP antibody, using KPB6 cells pre-treated with DMSO or MEK inhibitor GSK1120212 for 5.5 h (25 nM). Data represent the mean ± SD of biological triplicate IPs.

(**C**) qPCR analysis of Gapdh mRNA (control, lacking AU-rich elements) from RNA immunoprecipitates using IgG control or anti-TTP antibody, or anti-KSRP antibody, using KPB6 cells pre-treated with DMSO or MEK inhibitor GSK1120212 for 5.5 h (25 nM). Data represent the mean ± SD of biological triplicate IPs.

(**D**) qPCR analysis of PD-L1 expression in H358 cells 24 after transfection with empty, wild-type KSRP or phospho-mutant $KSRP^{S193A}$ constructs. Data represent the mean ± SEM of two independent experiments. $^{****}P<0.0001$; $^{***}P<0.001$; NS, not significant.

Unless otherwise stated, data were compared using unpaired, two-tailed Student's *t*-tests.

Figure S5, relating to Figure 5. RAS-ROS-p38 Signalling Controls TTP Activity

(**A**) MS/MS spectra for phosphopeptides STphSLVEGR (S52) and QSIphSFSGLPSGR (S178). -98 indicates the loss of H_3PO_4.

(**B**) Flow cytometry analysis of PD-L1 surface protein, and intracellular ROS measured by staining with H_2DCFDA, in MCF10A ER-HRASG12V cells treated with 4-OHT or vehicle ± NAC (10 mM) for 24 h. The same dataset is represented as a dot-plot and a histogram and data are representative of two independent experiments.

(**C**) Western blotting analysis of MCF10A cells harbouring an inducible version of the kinase domain of MEKK3 (ER-ΔMEKK3), 24 h after the addition of 4-OHT (100 nM) or vehicle.

(**D**) qPCR analysis of PD-L1 mRNA expression 24 h after treatment with NAC (10 mM), reduced glutathione (1 mM) or MK2 inhibitor III (1 µM). Data represent the mean ± SEM of two independent experiments. ***P*< 0.01, ****P*<0.005, *****P*<0.001; two-tailed Student's *t*-tests comparing to DMSO control condition.

(**E**) Western blotting analysis of CT26 TTP KO cells harbouring doxycycline-inducible WT or phospho-mutant Myc-TTP constructs, treated with doxycycline or vehicle for 24 h. Arrow indicates Myc-TTP.

Figure S6, relating to Figure 6. RAS Pathway Activation is Associated with PD-L1 Upregulation in Human Cancers

(**A**) Heat maps showing LUAD and COAD samples from the TCGA dataset clustered into RAS high or low pathway activity groups using RNA sequencing expression data and published RAS activity gene expression signatures (Loboda et al., 2010; Sweet-Cordero et al., 2005). *KRAS* mutation status (codons 12, 13 and 61) is shown for each sample.

(**B**) qPCR analysis of PD-L1 and IFNGR1 expression in ER-KRAS^{G12V} type II pneumocytes 24 h after treatment with vehicle, 4-OHT (100 nM), or 4-OHT with IFN-γ blocking antibody (10 µg/ml) or with ruxolitinib (500 nM). Mean ± SEM of two independent experiments. The panel on the right shows a qPCR analysis of PD-L1 expression in ER-KRAS^{G12V} type II pneumocytes following a 30 h treatment with IFN-γ (20 ng/ml) and IFN-γ with IFN-γ blocking antibody (10 µg/ml) or with ruxolitinib (500 nM) to verify blocking of IFN-γ – IFGR1 signalling.

(**C**) TTP mRNA expression in human patient lung and colon normal tissue versus adenocarcinoma, from publically available datasets (Selamat et al., 2012; Skrzypczak et al., 2010). Wilcoxon signed-rank test.

(**D**) qPCR analysis of PD-L1 and TTP expression in FACS purified CD45$^-$ CD31$^-$DAPI$^-$EpCAM$^+$ cells derived from lung tumours or matched normal adjacent lung tissue from *Kras*$^{LSL\text{-}G12D/+}$; *Trp53*$^{F/F}$ mice. Each point represents data from an individual mouse and is normalised to the matched normal lung tissue. ****P*<0.0005; unpaired, two-tailed Student's *t*-tests.

(**E**) Flow cytometry analysis of PD-L1 expression on CD45-CD31-DAPI- cells derived from macroscopically dissected lung tumours or normal adjacent lung tissue from *Kras*$^{LSL\text{-}G12D/+}$; *Trp53*$^{F/F}$ mice. Each point represents data from an individual mouse and is normalised to the matched normal lung tissue. Data are pooled from two independent experiments. MFI; Mean Fluorescence Intensity. **P*<0.05; unpaired, two-tailed Student's *t*-test.

Figure S7, relating to Figure 7. Restoration of Tumour Cell TTP Expression Enhances Anti-tumour Immunity

(**A**) Stability of murine PD-L1 mRNA measured by qPCR analysis. CT26 TTP (tetON) cells were pretreated with doxycycline (Dox.; 1 µg/ml) or vehicle for 16 h and then MEK inhibitor (GSK1120212, trametinib; 25 nM) for an additional 30 min before actinomycin D (ActD; 10 µg/ml) was added. Data are normalised to time 0 h when ActD was added and represent the mean ± SEM of two independent experiments.

(**B**) Western blotting analysis of stable MC38 cell lines expressing Myc-tagged, mouse TTP under a tetracycline-inducible promoter (TTP tet-ON). Cells were treated with doxycycline (Dox., 0.1 µg/ml or 1 µg/ml) or vehicle in starvation medium for 24 h before analysis. Arrow indicates Myc-TTP.

(**C**) qPCR analysis of PD-L1 expression from stable MC38 (TTP tet-ON) cell lines treated with doxycycline (Dox., 1 µg/ml) or vehicle in starvation medium for 40 h before analysis. Data represent the mean ± SEM of biological duplicates.

(**D**) Confluency was measured using IncuCyte for CT26 stable derivative cell lines treated with the indicated concentrations of doxycycline or vehicle at t = 0 h. Data represent the mean ± SD of biological triplicates and are representative of two independent experiments.

(**E**) Confluency was measured using IncuCyte for MC38 stable derivative cell lines treated with the indicated concentrations of doxycycline or vehicle at t = 0 h. Data represent the mean ± SD of biological triplicates.

(**F**) Representative histograms from flow cytometry analysis of PD-L1 surface expression in TTP (tetON) CT26 stable cells lines expressing endogenous PD-L1, PD-L1 + wild-type 3'UTR cDNA, or PD-L1 Δ3'UTR cDNA, after treatment with doxycycline (Dox., 1 µg/ml) or vehicle for 72 h. Data are representative of two independent experiments and also form part of Figure 7B.

(**G**) Tumour growth curves for the indicated CT26-derived cell lines shown in Figure S7C, subcutaneously transplanted into BALB/c mice (*n* = 5 WT 3'UTR + Dox and Δ3'UTR + H_2O; *n* = 4 WT 3'UTR + H_2O and Δ3'UTR + Dox; *n* = 3

empty + H_2O and empty + Dox). Vehicle or doxycycline (Dox., 50 mg/kg) was administered by oral gavage and commenced from day three after tumour cell injection. Mean ± SEM. ****P<0.0001, ***P<0.001, *P<0.05; two-way ANOVA.
(**H**) Representative histological analysis of CD3+ cells in subcutaneous tumours at the end-point from the experiment described in Figure 7C, and quantified in Figure 7G. Scale bar is 500 μm.
(**I**) Proposed molecular model. Oncogenic RAS activity leads to hyperphosphorylation, whereas PP2A activity promotes hypophosphorylation of TTP (Bourcier et al., 2011; Deleault et al., 2008; Essafi-Benkhadir et al., 2007; Hardle et al., 2015; Rahman et al., 2015; Sun et al., 2007), constituting a rapid switch controlling TTP activity. Low TTP expression and activity in tumour cells represents a permissive context for PD-L1 expression and immune evasion.

Table S1, relating to Figure 5 and Figure S5. TTP phosphopeptides. Identified mouse TTP phosphopeptides from MS analyses pooled from two independent biological experiments. Identifications are 1 % FDR controlled. PEP indicates the probability that the identification is incorrect. Phosphosite assignment probabilities are indicated in parenthesis.

PEP	Score	Position	Modified sequence	Phospho (STY) Probabilities	Number of Phospho (STY)
0.01167	100	52	_STS(ph)LVEGR_	STS(1)LVEGR	1
1.41E-10	70	80	_PGPELS(ph)PSPT(ph)SPTATPTTSSR_	PGPELS(0.998)PS(0.006)PT(0.477)S(0.43)PT(0.068)AT(0.011)PT(0.002)T(0.002)S(0.002)S(0.003)R	2
1.41E-10	69	82	_PGPELSPS(ph)PTSPTATPTTSSR_	PGPELS(0.036)PS(0.911)PT(0.04)S(0.009)PT(0.002)AT(0.001)PTTSSR	1
1.07E-52	186	85	_PGPELSPSPTS(ph)PTATPTTSSR_	PGPELSPSPT(0.128)S(0.857)PT(0.013)ATPTTSSR	1
0.0404	74	105	_TYS(ph)ESGRCR_	TYS(1)ESGRCR	1
6.90E-08	130	178	_QSIS(ph)FSGLPSGR_	QSIS(1)FSGLPSGR	1
1.50E-22	96	189	_RSS(ph)PPPPGFS(ph)GPSLSSCSFSPSSSPPPPGDLPLSPSAFSAAPGTPVTR_	RS(0.17)S(0.767)PPPPGFS(0.195)GPS(0.146)LS(0.131)S(0.124)CS(0.111)FS(0.1)PS(0.09)S(0.085)S(0.081)PPPPGDLPLSPSAFSAAPGTPVTR	2
1.50E-22	96	196	_RSSPPPPGFS(ph)GPS(ph)LSSCSFSPSSSPPPPGDLPLSPSAFSAAPGTPVTR_	RS(0.051)S(0.25)PPPPGFS(0.367)GPS(0.173)LS(0.169)S(0.167)CS(0.166)FS(0.165)PS(0.165)S(0.164)S(0.164)PPPPGDLPLSPSAFSAAPGTPVTR	2
1.50E-22	96	199	_RSS(ph)PPPPGFS(ph)GPS(ph)LSSCSFSPSSSPPPPGDLPLSPSAFSAAPGTPVTR_	RS(0.072)S(0.368)PPPPGFS(0.279)GPS(0.303)LS(0.3)S(0.297)CS(0.29)FS(0.282)PS(0.274)S(0.27)S(0.265)PPPPGDLPLSPSAFSAPGTPVTR	2
9.68E-15	69	201	_RSSPPPPGFS(ph)GPS(ph)LSSCSFSPSSSPPPPGDLPLSPSAFSAAPGTPVTR_	RS(0.013)S(0.177)PPPPGFS(0.202)GPS(0.201)LS(0.201)S(0.201)CS(0.201)FS(0.201)PS(0.201)S(0.201)S(0.201)PPPPGDLPLSPSAFSAAPGTPVTR	2
9.68E-15	69	202	_RSSPPPPGFS(ph)GPS(ph)LSSCSFSPSSSPPPPGDLPLSPSAFSAAPGTPVTR_	RS(0.013)S(0.177)PPPPGFS(0.202)GPS(0.201)LS(0.201)S(0.201)CS(0.201)FS(0.201)PS(0.201)S(0.201)S(0.201)PPPPGDLPLSPSAFSAAPGTPVTR	2
9.68E-15	69	204	_RSSPPPPGFS(ph)GPS(ph)LSSCSFSPSSSPPPPGDLPLSPSAFSAAPGTPVTR_	RS(0.013)S(0.177)PPPPGFS(0.202)GPS(0.201)LS(0.201)S(0.201)CS(0.201)FS(0.201)PS(0.201)S(0.201)S(0.201)PPPPGDLPLSPSAFSAAPGTPVTR	2
9.68E-15	69	206	_RSSPPPPGFS(ph)GPS(ph)LSSCSFSPSSSPPPPGDLPLSPSAFSAAPGTPVTR_	RS(0.013)S(0.177)PPPPGFS(0.202)GPS(0.201)LS(0.201)S(0.201)CS(0.201)FS(0.201)PS(0.201)S(0.201)S(0.201)PPPPGDLPLSPSAFSAAPGTPVTR	2
9.68E-15	69	208	_RSSPPPPGFS(ph)GPS(ph)LSSCSFSPSSSPPPPGDLPLSPSAFSAAPGTPVTR_	RS(0.013)S(0.177)PPPPGFS(0.202)GPS(0.201)LS(0.201)S(0.201)CS(0.201)FS(0.201)PS(0.201)S(0.201)S(0.201)PPPPGDLPLSPSAFSAAPGTPVTR	2
9.68E-15	69	209	_RSSPPPPGFS(ph)GPS(ph)LSSCSFSPSSSPPPPGDLPLSPSAFSAAPGTPVTR_	RS(0.013)S(0.177)PPPPGFS(0.202)GPS(0.201)LS(0.201)S(0.201)CS(0.201)FS(0.201)PS(0.201)S(0.201)S(0.201)PPPPGDLPLSPSAFSAAPGTPVTR	2
9.68E-15	69	210	_RSSPPPPGFS(ph)GPS(ph)LSSCSFSPSSSPPPPGDLPLSPSAFSAAPGTPVTR_	RS(0.013)S(0.177)PPPPGFS(0.202)GPS(0.201)LS(0.201)S(0.201)CS(0.201)FS(0.201)PS(0.201)S(0.201)S(0.201)PPPPGDLPLSPSAFSAAPGTPVTR	2
0.00722	63	248	_S(ph)TTPSTIWGPLGGLAR_	S(0.333)T(0.333)T(0.333)PSTIWGPLGGLAR	1
0.00722	63	249	_S(ph)TTPSTIWGPLGGLAR_	S(0.333)T(0.333)T(0.333)PSTIWGPLGGLAR	1
1.93E-20	158	250	_STT(ph)PSTIWGPLGGLAR_	ST(0.003)T(0.997)PSTIWGPLGGLAR	1
6.07E-19	89	264	_S(ph)PSAHSLGSDPDDYASSGSSLGGSDSPVFEAGVFGPPQTPAPPR_	S(0.446)PS(0.412)AHS(0.087)LGS(0.022)DPDDY(0.004)AS(0.005)S(0.005)GS(0.005)S(0.005)LGGS(0.005)DS(0.005)PVFEAGVFGPPQTPAPPR	1
6.07E-19	89	266	_S(ph)PSAHSLGSDPDDYASSGSSLGGSDSPVFEAGVFGPPQTPAPPR_	S(0.41)PS(0.41)AHS(0.165)LGS(0.006)DPDDY(0.001)AS(0.001)S(0.001)GS(0.001)S(0.001)LGGS(0.001)DS(0.001)PVFEAGVFGPPQTPAPPR	1
5.37E-14	83	269	_SPSAHS(ph)LGSDPDDYASSGSSLGGSDSPVFEAGVFGPPQTPAPPR_	S(0.272)PS(0.272)AHS(0.447)LGS(0.004)DPDDY(0.001)AS(0.001)S(0.001)GS(0.001)S(0.001)LGGS(0.001)DS(0.001)PVFEAGVFGPPQTPAPPR	1
1.50E-07	59	272	_SPSAHSLGS(ph)DPDDYASSGSSLGGSDSPVFEAGVFGPPQTPAPPR_	S(0.019)PS(0.019)AHS(0.078)LGS(0.343)DPDDY(0.073)AS(0.078)S(0.078)GS(0.078)S(0.078)LGGS(0.078)DS(0.078)PVFEAGVFGPPQTPAPPR	1
0.0015	41	279	_SPSAHSLGS(ph)DPDDYASSGSSLGGSDSPVFEAGVFGPPQTPAPPR_	S(0.044)PS(0.044)AHS(0.044)LGS(0.109)DPDDY(0.103)AS(0.109)S(0.109)GS(0.109)S(0.109)LGGS(0.109)DS(0.109)PVFEAGVFGPPQTPAPPR	1
0.0015	41	280	_SPSAHSLGS(ph)DPDDYASSGSSLGGSDSPVFEAGVFGPPQTPAPPR_	S(0.044)PS(0.044)AHS(0.044)LGS(0.109)DPDDY(0.103)AS(0.109)S(0.109)GS(0.109)S(0.109)LGGS(0.109)DS(0.109)PVFEAGVFGPPQTPAPPR	1
0.0015	41	282	_SPSAHSLGS(ph)DPDDYASSGSSLGGSDSPVFEAGVFGPPQTPAPPR_	S(0.044)PS(0.044)AHS(0.044)LGS(0.109)DPDDY(0.103)AS(0.109)S(0.109)GS(0.109)S(0.109)LGGS(0.109)DS(0.109)PVFEAGVFGPPQTPAPPR	1
0.0015	41	283	_SPSAHSLGS(ph)DPDDYASSGSSLGGSDSPVFEAGVFGPPQTPAPPR_	S(0.044)PS(0.044)AHS(0.044)LGS(0.109)DPDDY(0.103)AS(0.109)S(0.109)GS(0.109)S(0.109)LGGS(0.109)DS(0.109)PVFEAGVFGPPQTPAPPR	1
0.0015	41	287	_SPSAHSLGS(ph)DPDDYASSGSSLGGSDSPVFEAGVFGPPQTPAPPR_	S(0.044)PS(0.044)AHS(0.044)LGS(0.109)DPDDY(0.103)AS(0.109)S(0.109)GS(0.109)S(0.109)LGGS(0.109)DS(0.109)PVFEAGVFGPPQTPAPPR	1
0.0015	41	289	_SPSAHSLGS(ph)DPDDYASSGSSLGGSDSPVFEAGVFGPPQTPAPPR_	S(0.044)PS(0.044)AHS(0.044)LGS(0.109)DPDDY(0.103)AS(0.109)S(0.109)GS(0.109)S(0.109)LGGS(0.109)DS(0.109)PVFEAGVFGPPQTPAPPR	1

Table S2, relating to STAR Methods, Method Details. Cell lines and growth conditions.

Cell line	Normal medium	Starvation medium
H358	RPMI + 10 % FCS	N/A
A427	RPMI + 10 % FCS	N/A
H1792	RPMI + 10 % FCS	N/A
KPB6	DMEM + 10 % FCS	N/A
Type II pneumocytes	DCCM-1 + 10 % FCS	+ 0.5 % FCS for qPCR and FACS<br>+ 0 % FCS for mRNA half-life analysis
SW837	DMEM + 10 % FCS	N/A
H23	RPMI + 10 % FCS	N/A
293FT	DMEM + 10 % FCS	N/A
TTP KO and TTP WT MEFs	DMEM + 10 % FCS	+ 0.5 % FCS
MCF10A	F12:DMEM mix (1:1) and 5 % horse serum, 20 ng/ml EGF, 10 μg/ml insulin, 100 ng/ml cholera toxin, 0.5 μg/ml hydrocortisone	+ 5 % horse serum
CT26	RPMI + 10 % FCS	N/A
A549	DMEM + 10 % FCS	N/A
MC38	RPMI + 10 % FCS	+ 0.5 % FCS

N/A, not applicable.

Figure S1

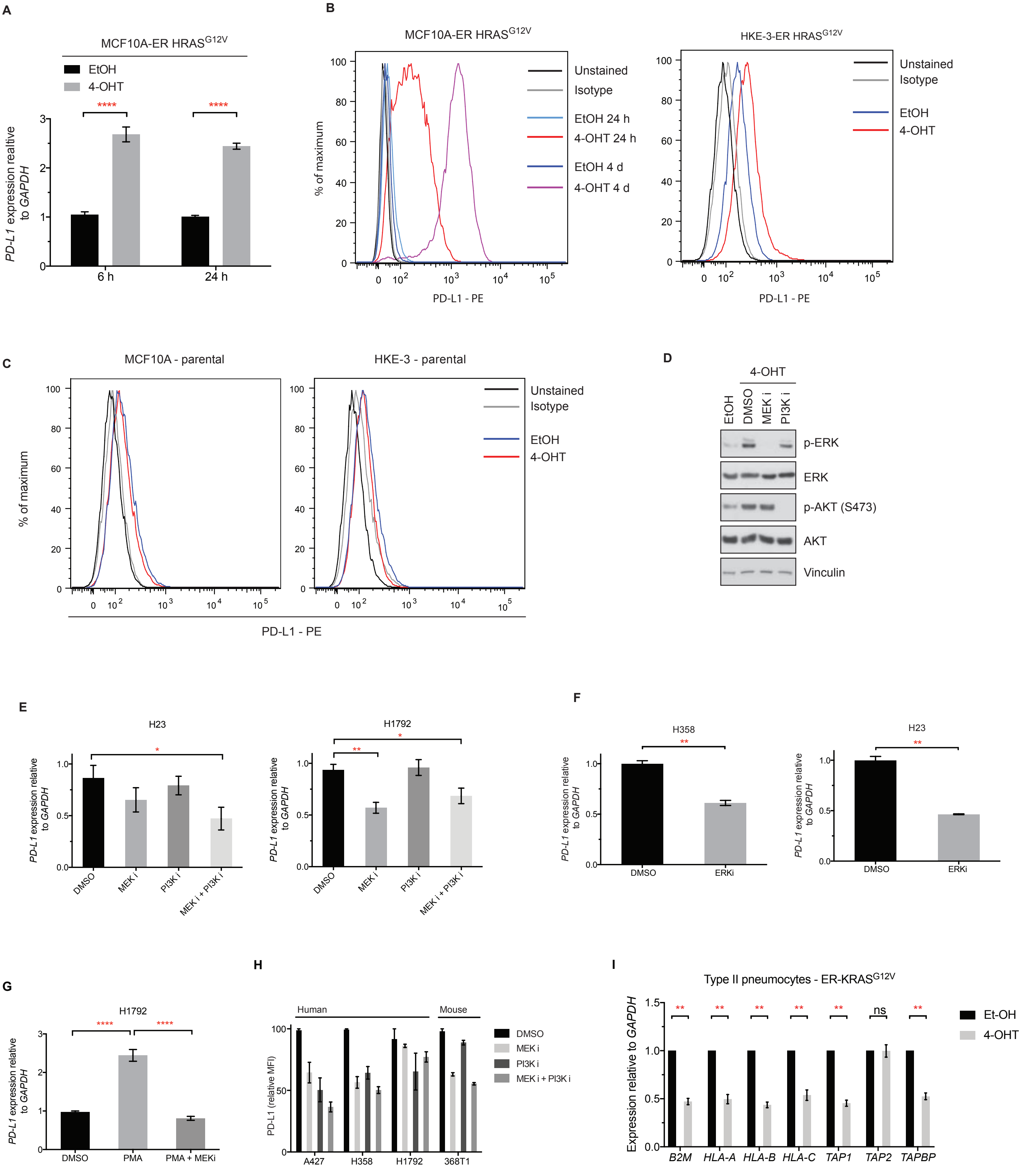

A
MCF10A-ER HRASG12V
EtOH
4-OHT
****
****
PD-L1 expression realtive to GAPDH
6 h
24 h
B
MCF10A-ER HRASG12V
Unstained
Isotype
EtOH 24 h
4-OHT 24 h
EtOH 4 d
4-OHT 4 d
% of maximum
PD-L1 - PE
HKE-3-ER HRASG12V
Unstained
Isotype
EtOH
4-OHT
PD-L1 - PE
C
MCF10A - parental
HKE-3 - parental
Unstained
Isotype
EtOH
4-OHT
% of maximum
PD-L1 - PE
D
4-OHT
EtOH
DMSO
MEK i
PI3K i
p-ERK
ERK
p-AKT (S473)
AKT
Vinculin
E
H23
*
PD-L1 expression relative to GAPDH
DMSO
MEK i
PI3K i
MEK i + PI3K i
H1792
**
*
F
H358
**
PD-L1 expression relative to GAPDH
DMSO
ERKi
H23
**
G
H1792
****
****
PD-L1 expression relative to GAPDH
DMSO
PMA
PMA + MEKi
H
Human
Mouse
DMSO
MEK i
PI3K i
MEK i + PI3K i
PD-L1 (relative MFI)
A427
H358
H1792
368T1
I
Type II pneumocytes - ER-KRASG12V
Expression relative to GAPDH
**
ns
Et-OH
4-OHT
B2M
HLA-A
HLA-B
HLA-C
TAP1
TAP2
TAPBP

Figure S2

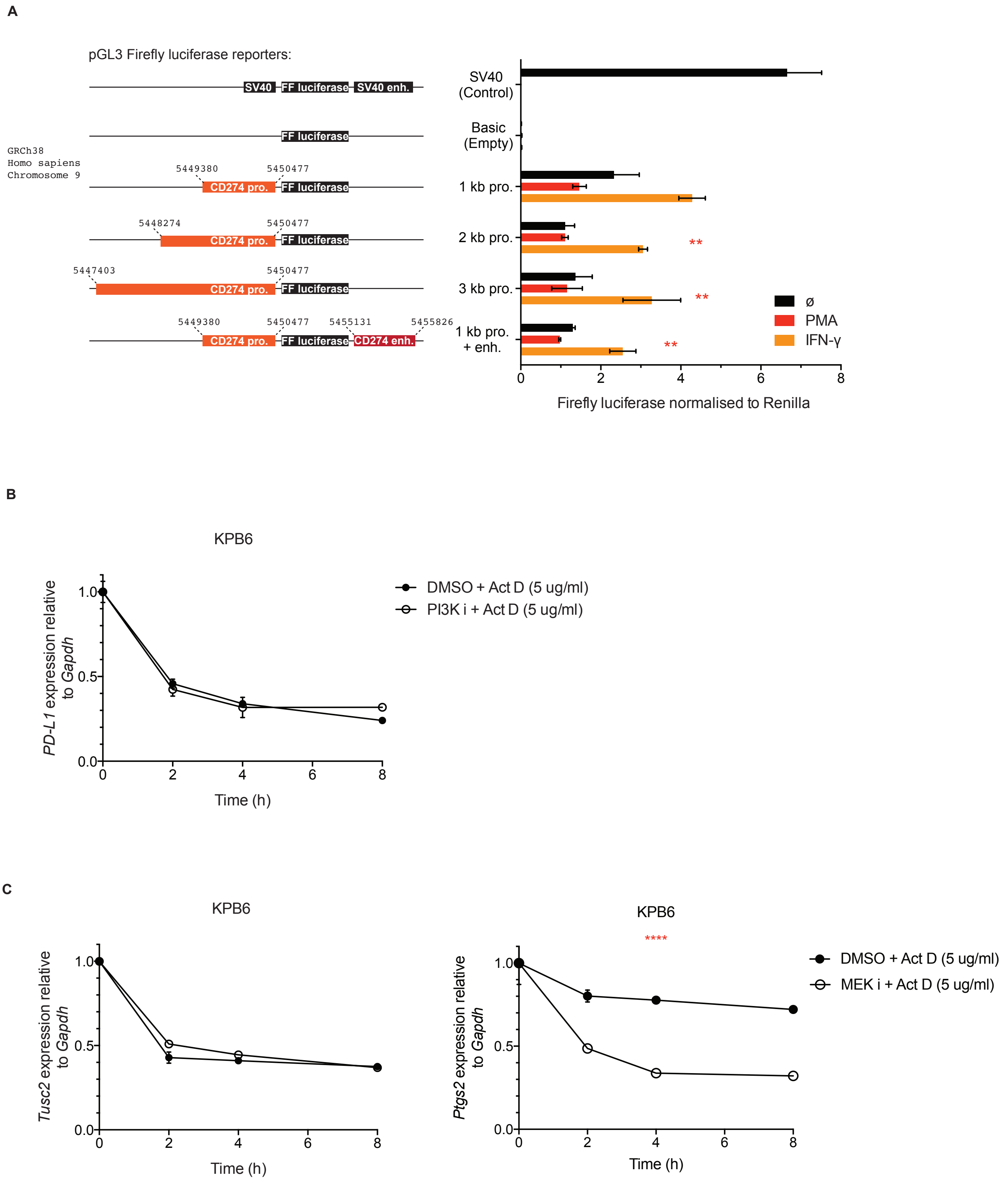
A
pGL3 Firefly luciferase reporters:
SV40
FF luciferase
SV40 enh.
GRCh38
Homo sapiens
Chromosome 9
5449380
5450477
CD274 pro.
5448274
5447403
5455131
5455826
CD274 enh.
SV40 (Control)
Basic (Empty)
1 kb pro.
2 kb pro.
3 kb pro.
1 kb pro. + enh.
**
ø
PMA
IFN-γ
0
2
4
6
8
Firefly luciferase normalised to Renilla
B
KPB6
PD-L1 expression relative to Gapdh
1.0
0.5
0.0
Time (h)
DMSO + Act D (5 ug/ml)
PI3K i + Act D (5 ug/ml)
C
KPB6
Tusc2 expression relative to Gapdh
Ptgs2 expression relative to Gapdh
****
MEK i + Act D (5 ug/ml)

Figure S3

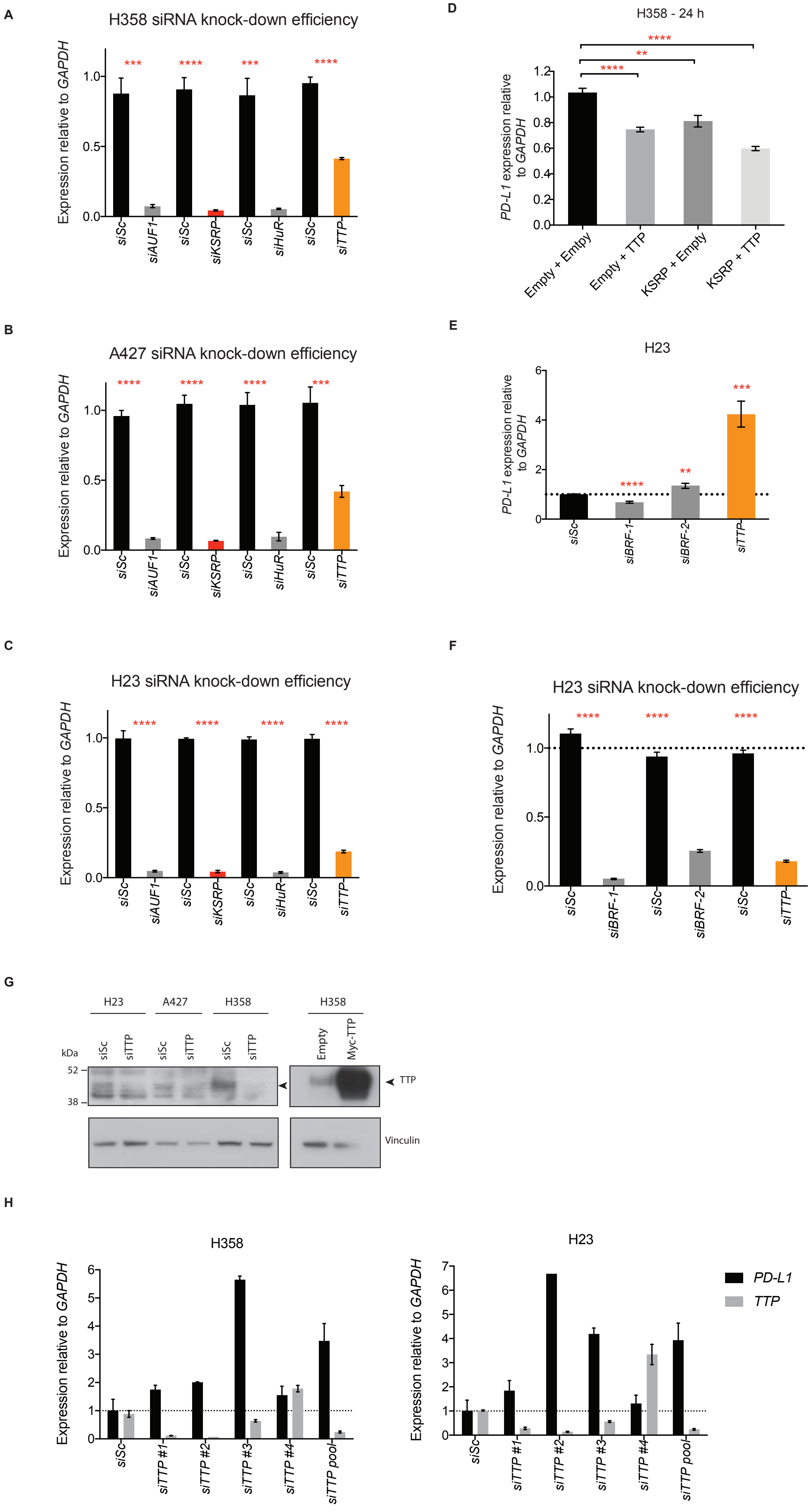

A
H358 siRNA knock-down efficiency
Expression relative to GAPDH
1.0
0.5
0.0
***
****
***
****
siSc
siAUF1
siSc
siKSRP
siSc
siHuR
siSc
siTTP
B
A427 siRNA knock-down efficiency
Expression relative to GAPDH
1.0
0.5
0.0
****
****
****
***
siSc
siAUF1
siSc
siKSRP
siSc
siHuR
siSc
siTTP
C
H23 siRNA knock-down efficiency
Expression relative to GAPDH
1.0
0.5
0.0
****
****
****
****
siSc
siAUF1
siSc
siKSRP
siSc
siHuR
siSc
siTTP
D
H358 - 24 h
PD-L1 expression relative to GAPDH
1.2
1.0
0.8
0.6
0.4
0.2
0.0
****
**
****
Empty + Empty
Empty + TTP
KSRP + Empty
KSRP + TTP
E
H23
PD-L1 expression relative to GAPDH
6
4
2
0
****
**
***
siSc
siBRF-1
siBRF-2
siTTP
F
H23 siRNA knock-down efficiency
Expression relative to GAPDH
1.0
0.5
0.0
****
****
****
siSc
siBRF-1
siSc
siBRF-2
siSc
siTTP
G
H23
A427
H358
H358
siSc
siTTP
siSc
siTTP
siSc
siTTP
Empty
Myc-TTP
kDa
52
38
TTP
Vinculin
H
H358
Expression relative to GAPDH
6
5
4
3
2
1
0
siSc
siTTP #1
siTTP #2
siTTP #3
siTTP #4
siTTP pool
H23
Expression relative to GAPDH
7
6
5
4
3
2
1
0
siSc
siTTP #1
siTTP #2
siTTP #3
siTTP #4
siTTP pool
PD-L1
TTP

Figure S4

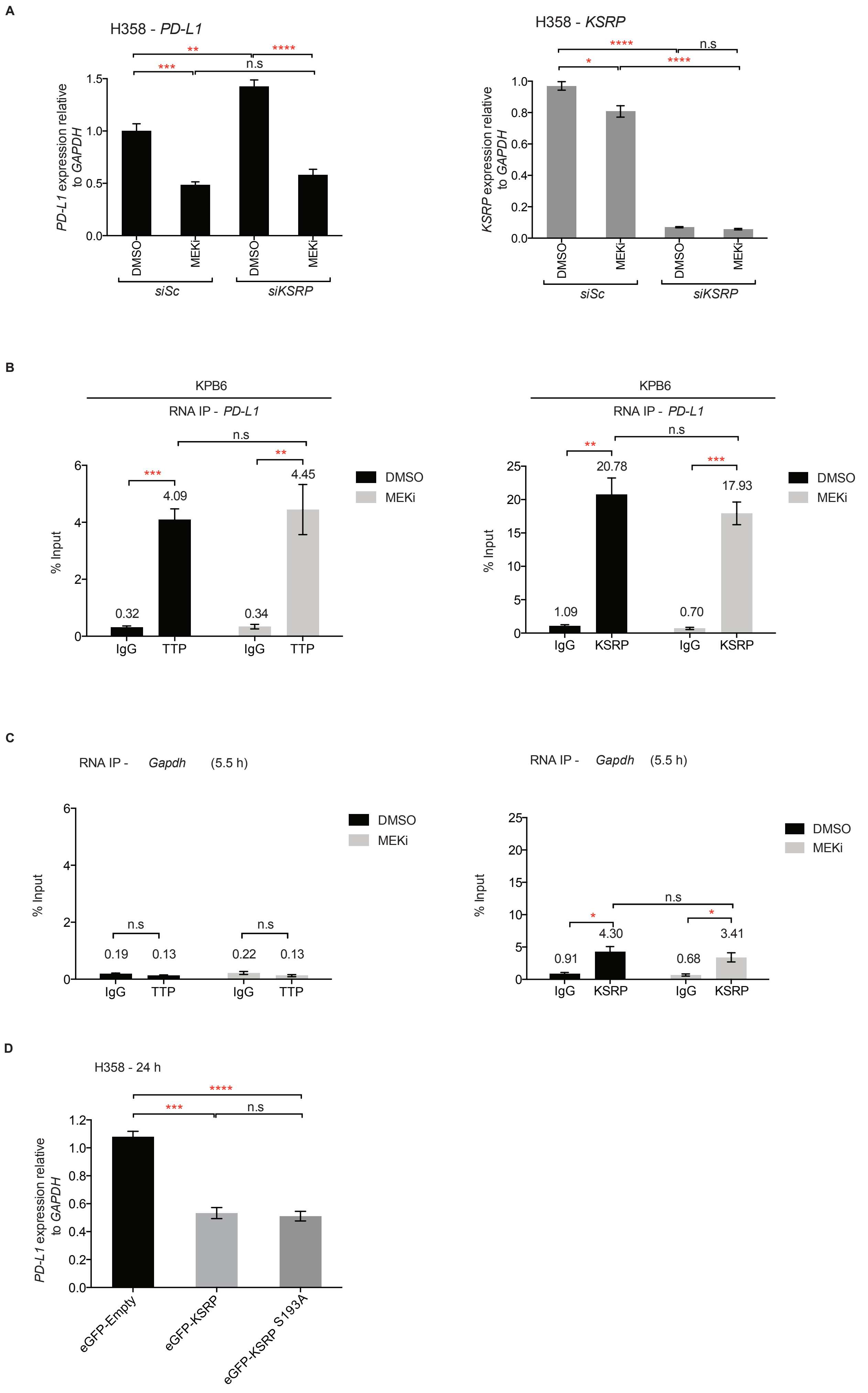
A
H358 - *PD-L1*
PD-L1 expression relative to *GAPDH*
1.5
1.0
0.5
0.0
DMSO
MEKi
DMSO
MEKi
siSc
siKSRP
**
****
***
n.s
H358 - *KSRP*
KSRP expression relative to *GAPDH*
1.0
0.8
0.6
0.4
0.2
0.0
DMSO
MEKi
DMSO
MEKi
siSc
siKSRP
****
n.s
*
****
B
KPB6
RNA IP - *PD-L1*
% Input
6
4
2
0
n.s
**
***
4.09
4.45
0.32
0.34
IgG
TTP
IgG
TTP
DMSO
MEKi
KPB6
RNA IP - *PD-L1*
% Input
25
20
15
10
5
0
n.s
**
***
20.78
17.93
1.09
0.70
IgG
KSRP
IgG
KSRP
DMSO
MEKi
C
RNA IP - *Gapdh* (5.5 h)
% Input
6
4
2
0
n.s
n.s
0.19
0.13
0.22
0.13
IgG
TTP
IgG
TTP
DMSO
MEKi
RNA IP - *Gapdh* (5.5 h)
% Input
25
20
15
10
5
0
n.s
*
*
4.30
3.41
0.91
0.68
IgG
KSRP
IgG
KSRP
DMSO
MEKi
D
H358 - 24 h
PD-L1 expression relative to *GAPDH*
****
***
n.s
1.2
1.0
0.8
0.6
0.4
0.2
0.0
eGFP-Empty
eGFP-KSRP
eGFP-KSRP S193A

Figure S5

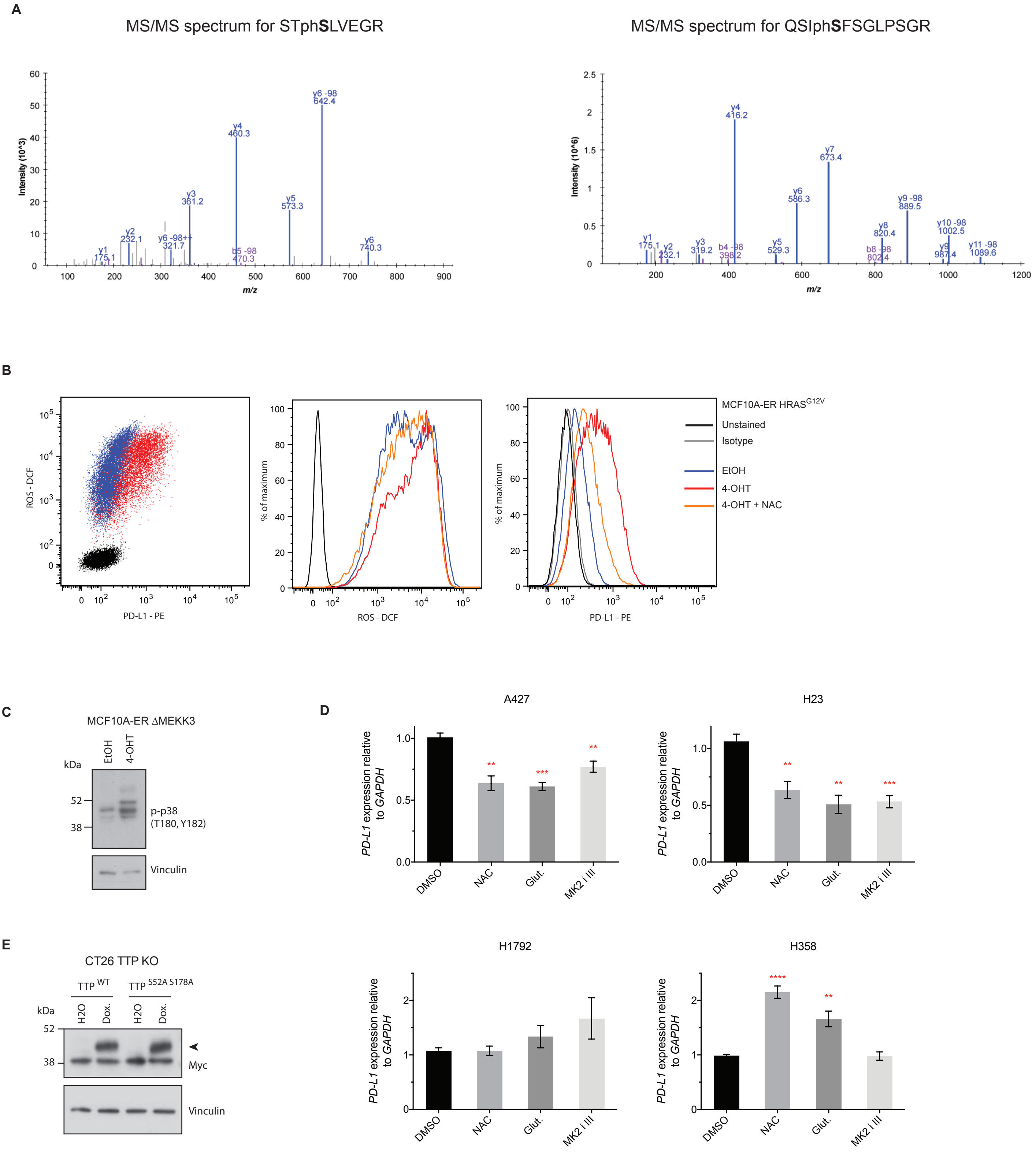
A
MS/MS spectrum for STphSLVEGR
MS/MS spectrum for QSIphSFSGLPSGR
Intensity (10^3)
Intensity (10^6)
m/z
y6 -98
642.4
y4
460.3
y3
361.2
y5
573.3
y6
740.3
y2
232.1
y6 -98++
321.7
y1
175.1
b5 -98
470.3
y4
416.2
y7
673.4
y6
586.3
y9 -98
889.5
y8
820.4
y10 -98
1002.5
y11 -98
1089.6
y9
987.4
b8 -98
802.4
y5
529.3
b4 -98
398.2
y3
319.2
y2
232.1
y1
175.1
B
ROS - DCF
PD-L1 - PE
% of maximum
MCF10A-ER HRASG12V
Unstained
Isotype
EtOH
4-OHT
4-OHT + NAC
C
MCF10A-ER ΔMEKK3
kDa
EtOH
4-OHT
52
38
p-p38
(T180, Y182)
Vinculin
D
A427
H23
H1792
H358
PD-L1 expression relative to GAPDH
DMSO
NAC
Glut.
MK2 i III
E
CT26 TTP KO
TTP WT
TTP S52A S178A
H2O
Dox.
Myc
Vinculin

Figure S6

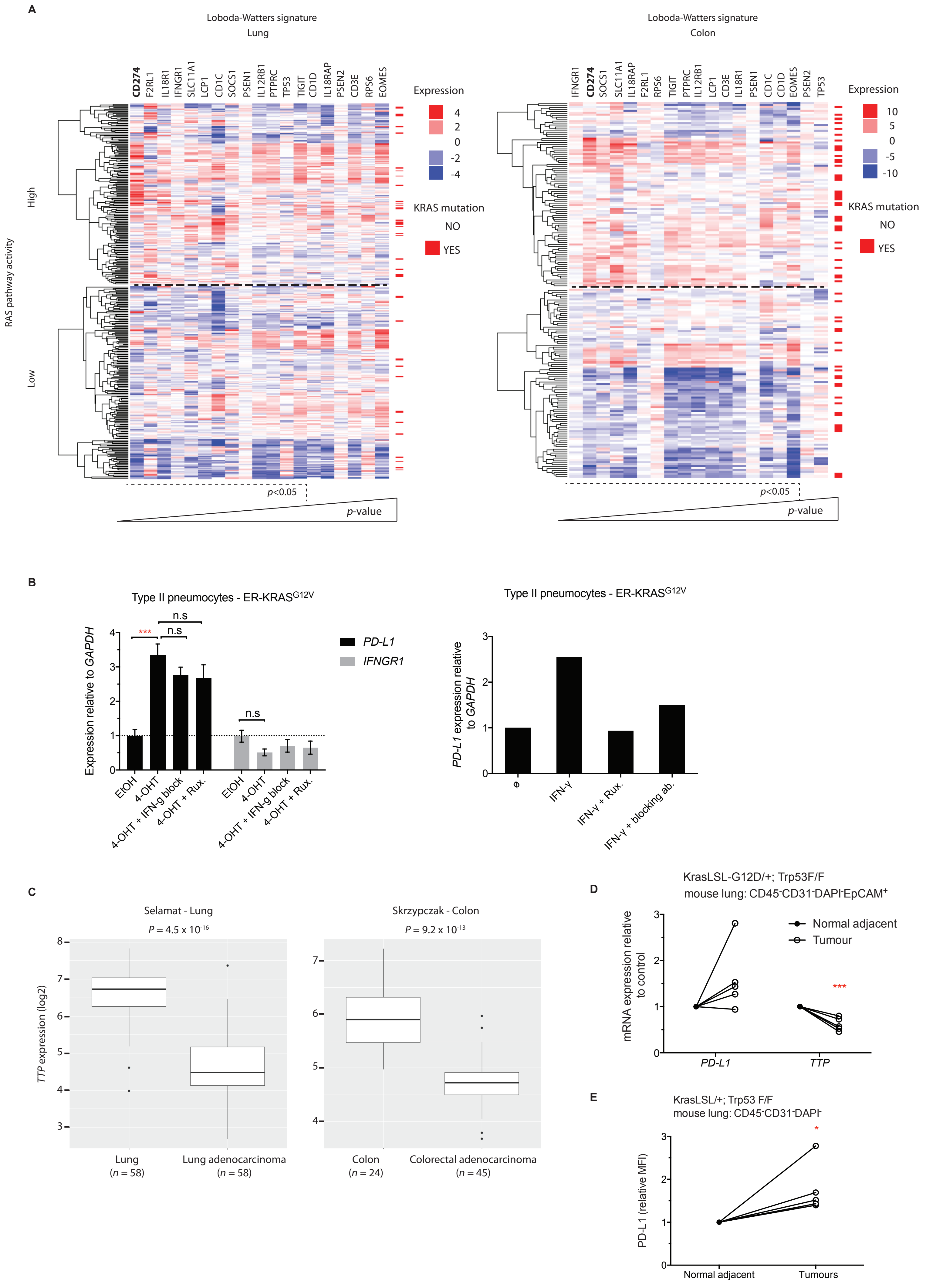

A
Loboda-Watters signature
Lung
CD274
F2RL1
IL18R1
IFNGR1
SLC11A1
LCP1
CD1C
SOCS1
PSEN1
IL12RB1
PTPRC
TP53
TIGIT
CD1D
IL18RAP
PSEN2
CD3E
RPS6
EOMES
Expression
4
2
0
-2
-4
KRAS mutation
NO
YES
RAS pathway activity
High
Low
p<0.05
p-value
Loboda-Watters signature
Colon
IFNGR1
CD274
SOCS1
SLC11A1
IL18RAP
F2RL1
RPS6
TIGIT
PTPRC
IL12RB1
LCP1
CD3E
IL18R1
PSEN1
CD1C
CD1D
EOMES
PSEN2
TP53
Expression
10
5
0
-5
-10
KRAS mutation
NO
YES
p<0.05
p-value
B
Type II pneumocytes - ER-KRASG12V
Expression relative to GAPDH
***
n.s
n.s
n.s
PD-L1
IFNGR1
EtOH
4-OHT
4-OHT + IFN-g block
4-OHT + Rux.
Type II pneumocytes - ER-KRASG12V
PD-L1 expression relative to GAPDH
ø
IFN-γ
IFN-γ + Rux.
IFN-γ + blocking ab.
C
Selamat - Lung
P = 4.5 x 10-16
TTP expression (log2)
Lung (n = 58)
Lung adenocarcinoma (n = 58)
Skrzypczak - Colon
P = 9.2 x 10-13
Colon (n = 24)
Colorectal adenocarcinoma (n = 45)
D
KrasLSL-G12D/+; Trp53F/F
mouse lung: CD45-CD31-DAPI-EpCAM+
mRNA expression relative to control
Normal adjacent
Tumour
***
PD-L1
TTP
E
KrasLSL/+; Trp53 F/F
mouse lung: CD45-CD31-DAPI-
*
PD-L1 (relative MFI)
Normal adjacent
Tumours

Figure S7

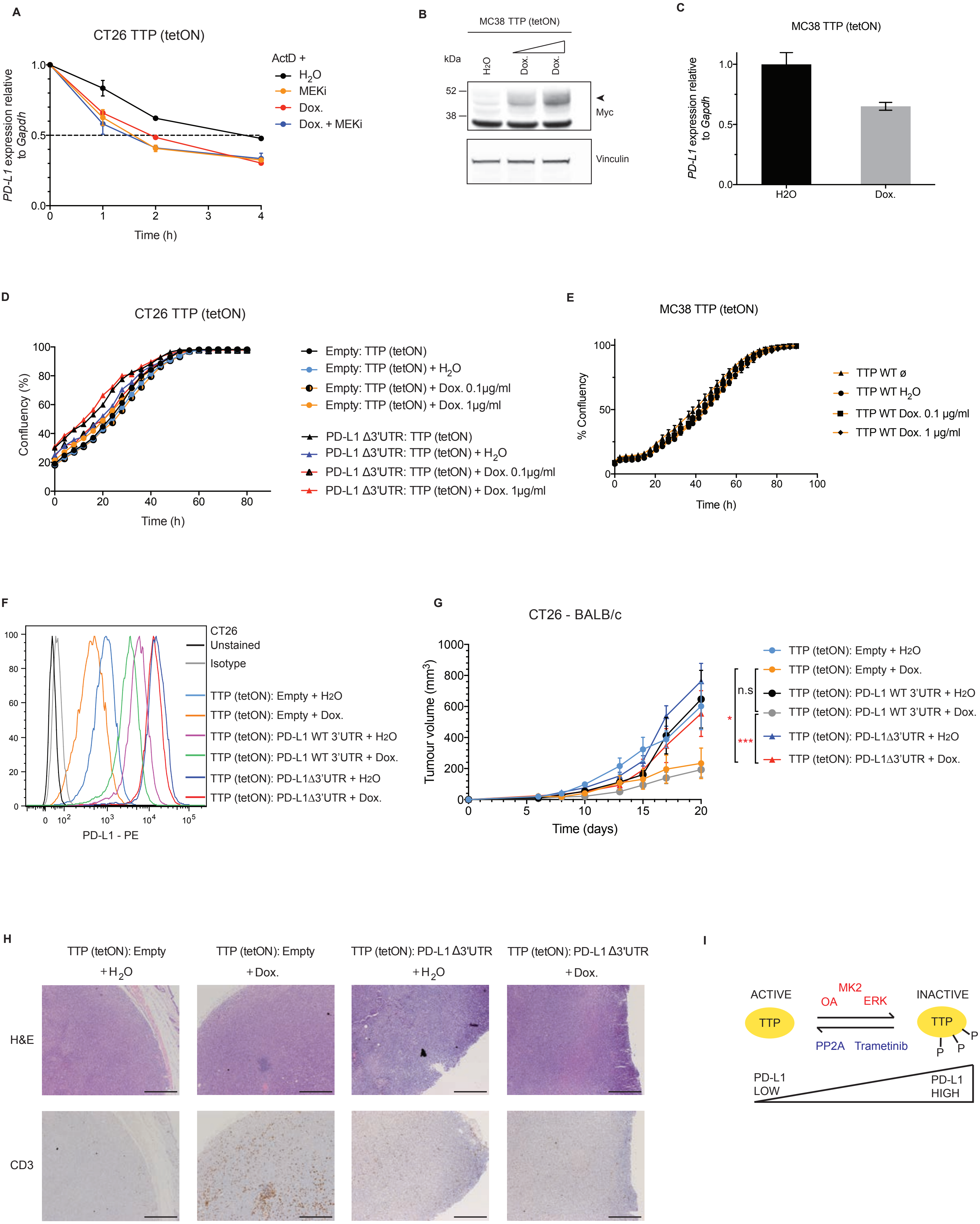
A
CT26 TTP (tetON)
PD-L1 expression relative to Gapdh
Time (h)
ActD +
H2O
MEKi
Dox.
Dox. + MEKi
B
MC38 TTP (tetON)
kDa
H2O
Dox.
Dox.
52
38
Myc
Vinculin
C
MC38 TTP (tetON)
PD-L1 expression relative to Gapdh
H2O
Dox.
D
CT26 TTP (tetON)
Confluency (%)
Time (h)
Empty: TTP (tetON)
Empty: TTP (tetON) + H2O
Empty: TTP (tetON) + Dox. 0.1µg/ml
Empty: TTP (tetON) + Dox. 1µg/ml
PD-L1 Δ3'UTR: TTP (tetON)
PD-L1 Δ3'UTR: TTP (tetON) + H2O
PD-L1 Δ3'UTR: TTP (tetON) + Dox. 0.1µg/ml
PD-L1 Δ3'UTR: TTP (tetON) + Dox. 1µg/ml
E
MC38 TTP (tetON)
% Confluency
Time (h)
TTP WT ø
TTP WT H2O
TTP WT Dox. 0.1 µg/ml
TTP WT Dox. 1 µg/ml
F
% of maximum
PD-L1 - PE
CT26
Unstained
Isotype
TTP (tetON): Empty + H2O
TTP (tetON): Empty + Dox.
TTP (tetON): PD-L1 WT 3'UTR + H2O
TTP (tetON): PD-L1 WT 3'UTR + Dox.
TTP (tetON): PD-L1Δ3'UTR + H2O
TTP (tetON): PD-L1Δ3'UTR + Dox.
G
CT26 - BALB/c
Tumour volume (mm3)
Time (days)
n.s
*
***
TTP (tetON): Empty + H2O
TTP (tetON): Empty + Dox.
TTP (tetON): PD-L1 WT 3'UTR + H2O
TTP (tetON): PD-L1 WT 3'UTR + Dox.
TTP (tetON): PD-L1Δ3'UTR + H2O
TTP (tetON): PD-L1Δ3'UTR + Dox.
H
TTP (tetON): Empty +H2O
TTP (tetON): Empty +Dox.
TTP (tetON): PD-L1 Δ3'UTR +H2O
TTP (tetON): PD-L1 Δ3'UTR +Dox.
H&E
CD3
I
ACTIVE
INACTIVE
TTP
TTP
MK2
OA
ERK
PP2A
Trametinib
P
P
P
PD-L1 LOW
PD-L1 HIGH
